# Wnt-pathway inhibitors with selective activity against triple-negative breast cancer: From thienopyrimidine to quinazoline inhibitors

**DOI:** 10.3389/fphar.2022.1045102

**Published:** 2022-10-28

**Authors:** Cédric Boudou, Luce Mattio, Alexey Koval, Valentin Soulard, Vladimir L. Katanaev

**Affiliations:** ^1^ Department of Cell Physiology and Metabolism, Translational Research Centre in Oncohaematology, Faculty of Medicine, University of Geneva, Geneva, Switzerland; ^2^ Institute of Life Sciences and Biomedicine, Far Eastern Federal University, Vladivostok, Russia

**Keywords:** Wnt signaling, triple-negative breast cancer, β-catenin, cancer survival, medicinal chemistry, structure activity relationship, thienopyrimidine, quinazoline

## Abstract

The Wnt-pathway has a critical role in development and tissue homeostasis and has attracted increased attention to develop anticancer drugs due to its aberrant activation in many cancers. In this study, we identified a novel small molecule series with a thienopyrimidine scaffold acting as a downstream inhibitor of the β-catenin-dependent Wnt-pathway. This novel chemotype was investigated using Wnt-dependent triple-negative breast cancer (TNBC) cell lines. Structure activity relationship (SAR) exploration led to identification of low micromolar compounds such as **5a**, **5d**, **5e** and a novel series with quinazoline scaffold such as **9d**. Further investigation showed translation of activity to inhibit cancer survival of HCC1395 and MDA-MB-468 TNBC cell lines without affecting a non-cancerous breast epithelial cell line MCF10a. This anti-proliferative effect was synergistic to docetaxel treatment. Collectively, we identified novel chemotypes acting as a downstream inhibitor of β-catenin-dependent Wnt-pathway that could expand therapeutic options to manage TNBC.

## 1 Introduction

The β-catenin-dependent Wnt-pathway regulates various physiological processes, from embryonic development to tissue homeostasis, and regeneration in adults (Parsons et al., 2021). However, aberrant activation of the Wnt-pathway plays a critical role in cancer cell proliferation, survival, and metastasis as well as in maintenance of cancer stem cells. This overactivation is predominantly driven by mutations, such as loss-of-function mutations for negative regulators like APC, AXIN1 or AXIN2, gain-of-function mutations for β-catenin (CTNNB1) or TCF, and eventually dysregulation of Wnt receptor abundance through mutations of RNF43, ZNRF3 or RSPO (Bugter et al., 2021; Parsons et al., 2021). In addition, several cancers have been linked to overexpression of the Wnt-pathway components, such as Wnts, their receptors (FZDs and LRP5/6), or Porcupine—the enzyme regulating Wnt secretion and activity (Stewart, 2014; Zhan et al., 2017; Shaw et al., 2019a; Wang et al., 2021). As a result of such aberrations, β-catenin accumulates in cell nucleus and promotes transcription of oncogenic target genes.

**FIGURE 1:**
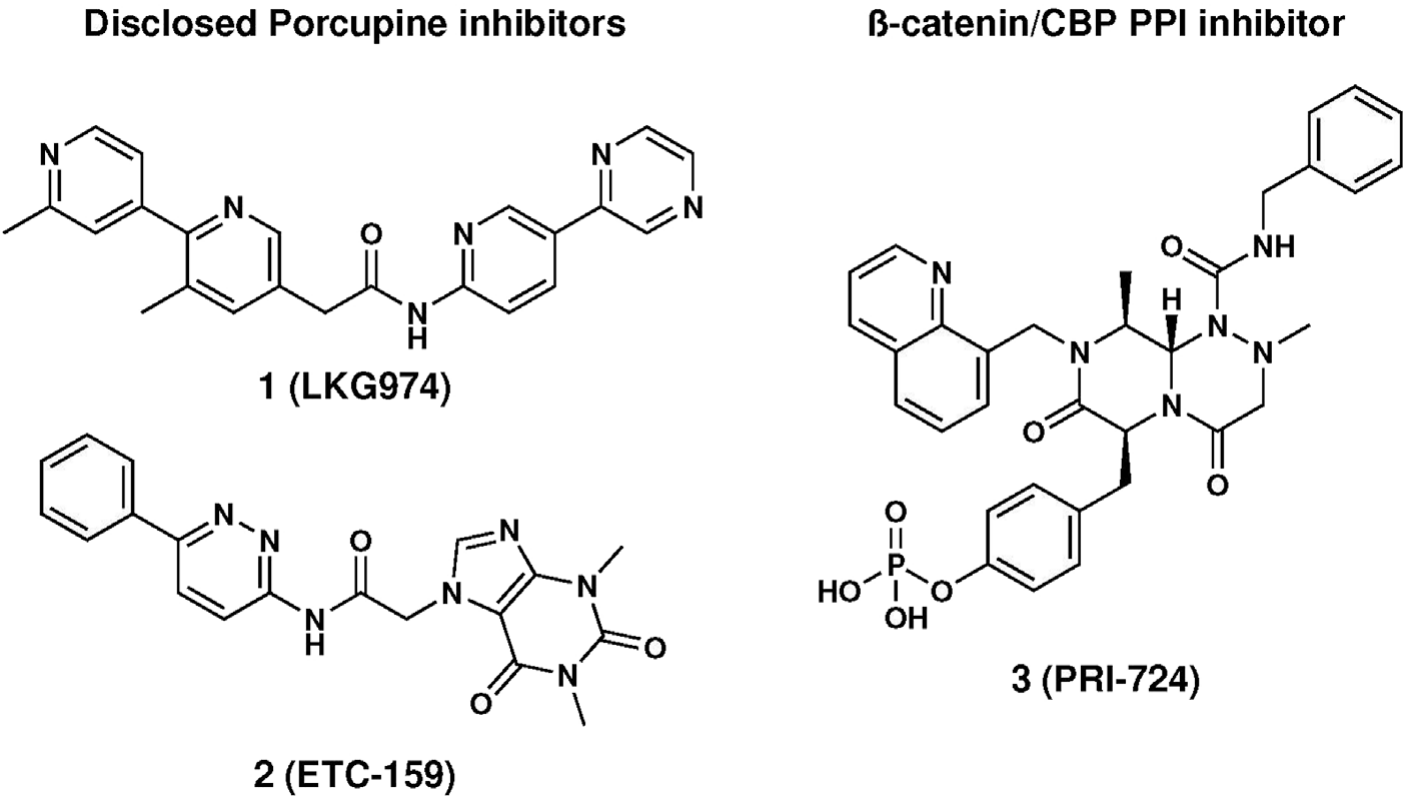
Structure of disclosed Wnt-inhibitors.

Since the discovery of the first WNT family member in 1982, Wnt signaling has attracted increased attention in the drug discovery field. Targeting the β-catenin-dependent pathway has been the focus of multiple drug discovery programs both in academia and industry, but only a few have reached clinical trials (Blagodatski et al., 2014; Liu et al., 2021). Among them, there are five small molecules acting as Porcupine inhibitors (*e.g.,* **1** and **2**, in Figure 1), which are profiled in phase 1/2 clinical trials for different cancer indications (Flanagan et al., 2022), while CWP232291, PRI-724, and SM08502 target downstream components of the Wnt-pathway (Shaw et al., 2019a). CWP232291 is a peptidomimetic targeting Sam68, an RNA-binding protein, and is currently profiled on acute myeloid leukaemia in phase 1/2. PRI-724 (**3**, in Figure 1) affecting interaction between β-catenin and the transcription co-activator CBP, has been evaluated in clinical trials against multiple cancers and cirrhosis. Finally, SM08502, acting through CDC-like kinase, reduces the Wnt-pathway-related gene expression and is profiled in phase 1 (Tam et al., 2020).

Our research has been aimed at discovering novel Wnt-pathway inhibitors (Koval and Katanaev, 2012; Katanaev et al., 2021; Larasati et al., 2022). We have previously developed a High-Throughput Screening (HTS) assay based on a triple-negative breast cancer (TNBC) cell line BT-20 that could be adapted to any β-catenin-dependent cancer cell line. In this assay, BT-20 cells are stably transfected with the Wnt-responsive TopFlash reporter, and activated by the in-house purified Wnt3a (Shaw et al., 2019b). A pilot screening of a commercial library of 1,000 compounds led to the identification of FSA as selective inhibitor of the Wnt signaling and cancer cell proliferation, *in vitro* and *in vivo* (Katanaev and Koval, 2021). This assay has also been used to drive the drug design of clofazimine derivatives acting as downstream inhibitors of the Wnt-pathway (Koval et al., 2021). In continuation of our efforts to identify novel chemotypes acting as β-catenin-dependent Wnt inhibitors and potential anticancer drugs, we here report a novel chemical series leading to compounds endowed with submicromolar activity in cancer cell lines, inhibiting the β-catenin-dependent Wnt signaling at downstream levels of the pathway.

## 2 Results

### 2.1 4a acts downstream of GSK3β in the β-catenin-dependent Wnt-pathway

The advantage of our platform lies in its ability to discriminate between inhibitors of the β-catenin-dependent Wnt signaling acting at upstream vs. downstream levels of the pathway, by stimulating the pathway at the different levels either with Wnt3a or with a GSK3β inhibitor, like CHIR99021 (Shaw et al., 2019b). Both stimulations abolish formation of the destruction complex, a multiprotein complex consisting of Axin, adenomatous polyposis *coli* (APC), glycogen synthase kinase three beta (GSK3β), and casein kinase one alpha (CK1α). Upon inactivation of the complex, β-catenin accumulates in the cytoplasm and translocates into the nucleus where it interacts with transcription factors. These downstream events are quantified by a Luciferase transcription-based readout assay (TopFlash assay).

**FIGURE 2:**
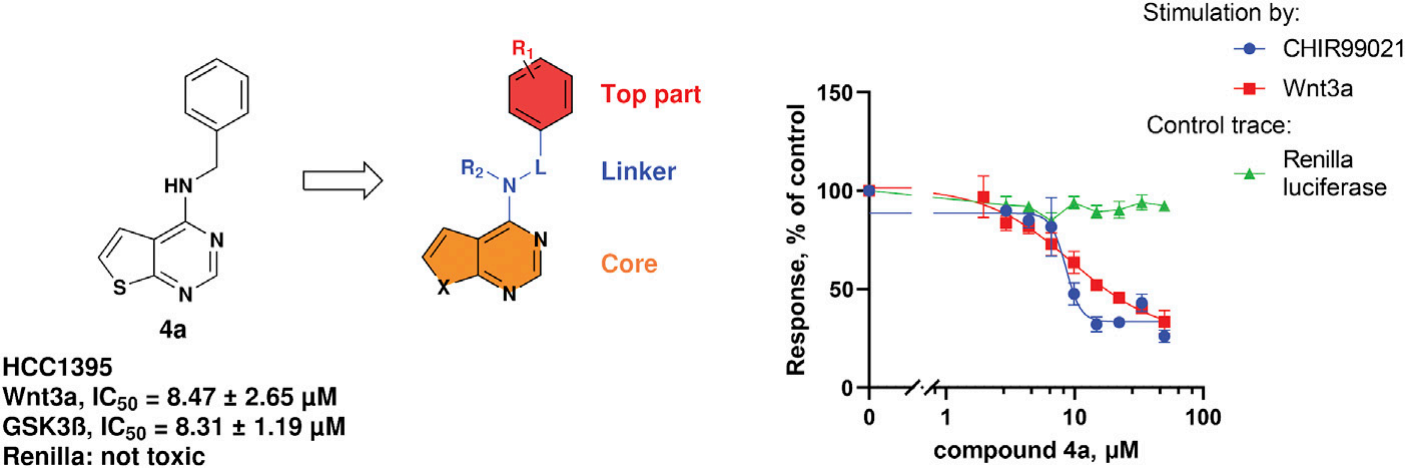
Structure of compound **4a**, area of exploration for library synthesis, and concentration-dependent inhibition of the TopFlash readout.

During our investigation toward novel Wnt-inhibitors, we identified a thienopyrimidine **4a** as a β-catenin-dependent Wnt-pathway inhibitor with micromolar potency in the TNBC cell line HCC1395. Both means of the pathway activation—at the upstream levels with Wnt3a and at the downstream levels with a GSK3β inhibitor CHIR99021—revealed a similar potency of **4a** to inhibit the signaling, respectively 8.31 µM and 8.47 µM (Figure 2). This result led to the conclusion that **4a** acts downstream of GSK3β in the Wnt-pathway.

We considered **4a** as a good starting point for a drug discovery exploration. The compound has no cytotoxic activity (measured by Renilla luciferase expressed under the Wnt-independent CMV promoter, Figure 2) and has a good potency with respect to its low molecular weight (241 g/mol). The simplicity of the scaffold combined with synthetic accessibility allows building a library of compounds and performing structure activity relationship (SAR) studies of the three main parts of the molecule: the top part, the linker and the core (Figure 2).

### 2.2 Chemistry

**SCHEME 1:**
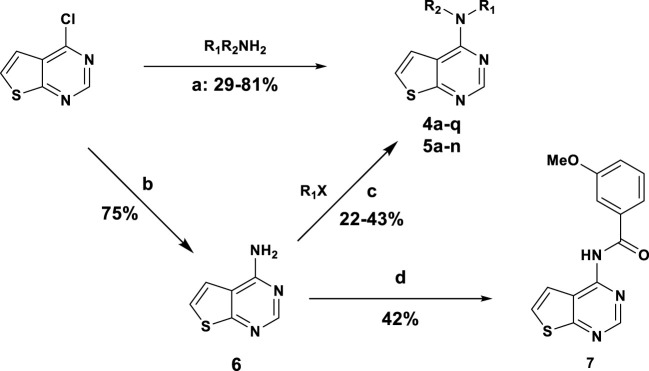
General synthesis of Thieno [2,3-d]pyrimidine derivatives: **(A)** DIPEA, DMF, 100°C, 4 h; **(B)** NH_4_OH, 90°C 12 h; **(C)** NaH 60%, THF, 0°C to rt, 12 h; **(D)** 3-methoxybenzenesulfonyl chloride, Et_3_N, DCM, rt, 24 h.

The synthesis of thieno [2,3-d]pyrimidine derivatives is illustrated in Scheme 1. Commercially available 4-chlorothieno [2,3-d]pyrimidine was subjected to aromatic substitution with different primary and secondary amines to afford the benzyl derivatives (**4a-e, 4g, 4i-l** and **4o-q**) and some of the linker replacement **5a-n** in the 29%–81% yield. In some cases, formation of side products did not permit purification of the final compounds, or the corresponding amines were not available. Therefore, as an alternative approach, 4-chlorothieno [2,3-d]pyrimidine was converted into 4-aminothieno [2,3-d]pyrimidine (compound **6**) with ammonium hydroxide in the 75% yield without purification. This second building block was reacted with differently substituted benzyl bromides or benzoyl chloride to afford compounds **4f**, **4k, 4m**, and **7** in moderate yields. With these synthetic approaches, it was possible to obtain a series of analogues of **4a** modified in the top part and in the linker portions (Figure 2).

**SCHEME 2:**
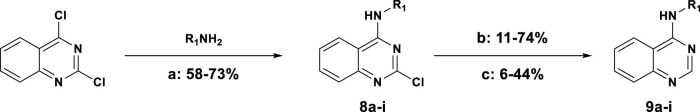
General synthesis of Quinazoline core: **(A)** DIPEA, CH_3_CN, rt, 12 h; **(B)** 10% Pd/C, ammonium formate, EtOH, reflux, 12 h; **(C)** 1) Zn, TMEDA, AcOH, MeOH, 40°C–45°C, 12 h, 2) 2-mercaptonicotinic acid, rt, 30 min.

To perform core modifications, the thienopyrimidine nucleus was replaced with a series of heterocycles. For the quinazoline core, the synthetic approach used was similar to the one used for the thienopyrimidine derivatives. Commercially available 2,4-dichloroquinazoline was subjected to the nucleophilic aromatic substitution with different primary amines to afford the intermediates **8a-i** in moderate to good yields (Scheme 2). Interestingly, although both C2 and C4 positions are electron-deficient, the reaction is regioselective toward the C4 position (Mohamed and Rao, 2015). In the second step, chlorine in position C2 was removed using reductive conditions. For intermediates **8a-f, 8f** and **8h-i**, ammonium formate in presence of palladium on charcoal at reflux was used to afford the expected compounds in the 11%–74% yield. For compounds **8e** and **8g**, bearing chlorine on the aromatic ring in the top part, a regioselective reduction of the chlorine in C2 of the quinazoline derivatives had to be performed. Therefore, compounds **9e** and **9g** were obtained in milder conditions with zinc, *N,N,N′,N′*-tetramethyl ethylenediamine, acetic acid in methanol at 45°C overnight.

Preparation of pyrimidines (**11** and **12**), isoquinoline, **13** and quinoline **16** derivatives followed similar synthetic strategies and are described in the Supplementary Material.

### 2.3 SAR investigation

The synthesized compounds were used to explore SAR of three defined areas of investigation (Figure 2). All compounds were tested in the HCC1395 TNBC cell line using the GSK3β inhibitor to induce β-catenin signaling since **4a** acted downstream of GSK3β (Figure 2). Both inhibition of β-catenin signaling measured by the TopFlash reporter and cytotoxicity measured by the Renilla reporter were assessed.

SAR of benzyl substitution is depicted in Table 1. Overall, *ortho* substituents were not tolerated or resulted in toxicity, like the 2-fluoro derivative **4e**. The only exception is the 2-methoxy analogue **4b**, which showed a 3-fold improvement in the activity (IC_50_ = 2.79 µM) compared to the unsubstituted benzyl derivative **4a** (IC_50_ = 8.31 µM), although with a partial inhibition of 40.7% compared to 72% of the parental compound. In the *meta* position, electron withdrawing groups were not tolerated (compounds **4f**, **4i**), except for compound **4i** bearing chlorine, which resulted in the activity similar to that of **4a**. However, potency was modestly improved by introducing electron donating groups, such as methoxy (**4c**) or methyl (**4n**) groups. In the *para* position, electron donating (methoxy, **4d**) or withdrawing (cyano, **4m**) groups abrogated the activity, whereas fluorine **4g**, chlorine **4j** and methyl **4o** groups showed a 2-fold improvement in the activity compared to **4a**. Therefore, derivatives combining the substituents which gave the best results in the inhibitory activity were designed and synthesized. Pleasingly, compounds **4p** and **4q**, bearing a methoxy group in the *ortho* position and fluoro or chloro in the *para* position, respectively, resulted in the IC_50_ of 2.59 µM and 1.33 µM.

**TABLE 1:** SAR exploration of the benzyl top part of thieno[2,3-d]pyrimidine.

Compound	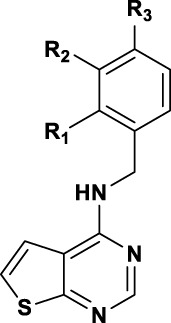			HCC1395 TopFlash^a^		HCC1395 Cytotoxicity^b^	
	R_1_	R_2_	R_3_	IC_50_ (µM)	Efficacy (%)	IC_50_ (µM)	Efficacy (%)
4a	H	H	H	8.31 ± 1.19	72.5 ± 7.9	>50	
4b	OMe	H	H	2.79 ± 0.67	40.7 ± 3.1	>50	
4c	H	OMe	H	4.96 ± 1.65	58.6 ± 22.8	>50	
4d	H	H	OMe	>50		ND	
4e	F	H	H			7.45 ± 2.08	
4f	H	F	H	>50		ND	
4g	H	H	F	4.15 ± 0.91	78.6 ± 29.8	>50	
4h	Cl	H	H			8.61 ± 4.38	67.1 ± 7.8
4i	H	Cl	H	8.40 ± 5.01	56.2 ± 4.0	>50	
4j	H	H	Cl	3.19 ± 1.64	74.6 ± 12.0	>50	
4k	CN	H	H	>50		ND	
4l	H	CN	H			10.04 ± 3.36	32.2 ± 10.5
4m	H	H	CN	>50		ND	
4n	H	Me	H	3.76 ± 1.90	52.9 ± 6.8	>50	
4o	H	H	Me	4.61 ± 1.91	72.7 ± 19.5	>50	
4p	H	OMe	Cl	2.59 ± 1.08	71.4 ± 11.1	4.04	41.8
4q	H	OMe	Cl	1.33 ± 0.07	68.5 ± 2.9	>50	

a Stimulation of the Wnt-pathway with CHIR99021 (GSK3β inhibitor) and inhibition of the Wnt-pathway is measured by the TopFlash assay.

b Cytotoxic activity measured by Renilla luciferase.

To investigate contribution of the linker between the thienopyrimidine core and the aromatic rings, we initially probed the influence of an additional methyl group, preparing a series of 1-phenylethylamine analogues (Table 2). Except for the 3-methoxy analogue **5b**, which was inactive, the other derivatives resulted in 2 to 7-fold more potent compounds. In particular, **5d** (4-chloro), **5e** (4-methyl) and **5a** (2-methoxy) showed submicromolar activities of 0.6, 0.63, and 0.77 µM, respectively. Unfortunately, this modification of the linker resulted in partial inhibition (<50% efficacy). Polar groups such as an amide linker (Liu et al., 2021) were not tolerated (Table 3). Alkylation of the amino group provided different results with benzyl or 2-phenylethan-1-amine top parts. Addition of ethyl group on compound **4c** resulted in toxicity at 8.30 µM with compound **5i**, whereas addition of a methyl group on compound **5g** resulted in the similar potency with compound **5j** (IC_50_ = 2.04 µM, 100% efficacy) (Table 3). This loss in potency could derive from different SAR between benzyl and 2-phenylethan-1-amine. Then, we investigated the impact of the linker length (Table 3) and found that compounds without a linker between the core and the top part, like **5f**, or bearing a three-carbon chain (**5h**) were inactive. On the other side, a two-carbons linker (**5g)** was three times more potent than **4a** with 89% of inhibition (IC_50_ = 2.64 and 8.31 µM respectively). Attracted by the potency of **5g**, we decided to introduce several in-house available substituted 2-phenylethan-1-amines. As for the benzyl derivatives, chlorine substitution was not tolerated in the *ortho* position (**5k**, IC_50_ = 42.87 µM), but resulted in good potency in the *meta* (**5l**, IC_50_ = 3.07 µM) and *para* (**5l**, IC_50_ = 1.79 µM) positions. Unlike the benzyl analogue **4c**, the methoxy group in the *meta* position was not tolerated (**5n**).

**TABLE 2:** SAR exploration of the 1-phenylethylamine top part of thieno[2,3-d]pyrimidine.

Compound	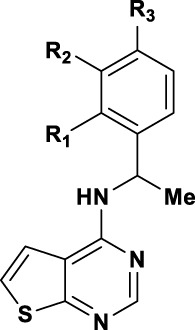			HCC1395 TopFlash^a^		HCC1395 Cytotoxicity^b^	
	R_1_	R_2_	R_3_	IC_50_ (µM)	Efficacy (%)	IC_50_ (µM)	Efficacy (%)
5a	OMe	H	H	0.77 ± 0.44	46.09 ± 3.44	>50	
5b	H	OMe	H	>50			
5c	H	H	F	2.10 ± 1.64	46.93 ± 8.41	>50	
5d	H	H	Cl	0.60 ± 0.33	55.42 ± 24.26	23.98 ± 4.98	100 ± 0
5e	H	H	Me	0.63 ± 0.21	46.98 ± 9.67	>50	

a Stimulation of the Wnt-pathway with CHIR99021 (GSK3β inhibitor) and inhibition of the Wnt-pathway is measured by the TopFlash assay.

b Cytotoxic activity measured by Renilla luciferase.

**TABLE 3:** SAR exploration of the linker part of thieno[2,3-d]pyrimidine.

Comp-ound	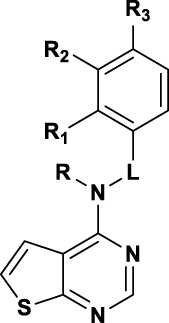					HCC1395 TopFlash^a^		HCC1395 Cytotoxicity^b^	
	R	L	R_1_	R_2_	R_3_	IC_50_ (µM)	Efficacy (%)	IC_50_ (µM)	Efficacy (%)
5f	H	-	H	H	H	>50		ND	
4a	H	-CH_2_-	H	H	H	8.31 ± 1.19	72.5 ± 7.9	>50	
5g	H	-(CH_2_)_2_-	H	H	H	2.64 ± 1.22	86.9 ± 6.1	>50	
5h	H	-(CH_2_)_3_-	H	H	H	>50		>50	
4c	H	-CH_2_-	H	OMe	H	4.96 ± 1.65	58.6 ± 22.8	>50	
7	H	-C(O)-	H	OMe	H	>50		ND	
5i	Et	-CH_2_-	H	OMe	H			8.30 ± 3.79	64.1 ± 9.3
5j	Me	-(CH_2_)_2_-	H	H	H	2.04 ± 2.55	100 ± 0	>50	
5k	H	-(CH_2_)_2_-	Cl	H	H	42.87 ± 50.17	93.2 ± 10.6	>50	
5l	H	-(CH_2_)_2_-	H	Cl	H	3.07 ± 2.39	77.5 ± 16.3	>50	
5m	H	-(CH_2_)_2_-	H	H	Cl	1.79 ± 1.45	82.0 ± 21.1	>50	
5n	H	-(CH_2_)_2_-	H	OMe	H	>50		ND	

a Stimulation of the Wnt-pathway with CHIR99021 (GSK3β inhibitor) and inhibition of the Wnt-pathway is measured by the TopFlash assay.

b Cytotoxic activity measured by Renilla luciferase.

We finally evaluated replacement of the core and, without surprise, the bioisosteric replacement of thieno [2,3-*d*]pyrimidine by quinazoline was well tolerated (Table 4). Other modifications of the bottom part led to drastic loss of potency (Table S1). Substituted quinazolines with 2-chloroquinazoline (**8a**), 7-dimethylthieno [3,2-d]pyrimidine (**10**) and single heteroaromatics 2-chloropyrimidine (**11**) or pyrimidine (**12**) were no longer active. Moreover, we observed that both nitrogens of the pyrimidine ring from quinazoline were necessary for the activity, since the isoquinoline **13** and quinoline **16** derivatives were inactive or toxic at 18.67 µM. Attracted by the potency of quinazoline **9b** with IC_50_ of 3.58 µM compared to 4.96 µM for thieno [2,3-*d*]pyrimidine **4c**, we evaluated SAR transfer synthesizing quinazoline derivatives bearing the best modifications obtained from the thieno [2,3-*d*]pyrimidine analogues previously described. With few exceptions, the resulting compounds had similar or better potencies and better efficacies (Table 4). For substituents of the phenyl ring, compounds bearing a substituent in the *para* position showed a good activity: 4-methoxy was active for the quinazoline version (**9c**, with IC_50_ = 4.76 µM, 81.5%) and 4-fluoro (**9b**), 4-chloro (**9e**) and 4-methyl (**9f**) were more potent with IC_50_ of 0.67, 2.18, and 1.22 µM, respectively. Also, with the quinazoline core, a longer linker did not lead to activity improvement, as we observed with the 4-chlorophenylethyl derivative **9g**, found to be inactive. On the other hand, addition of methyl to provide 1-phenylethylamine derivatives resulted either in a drastic reduction of activity (**9h**)**,** or in compounds with similar activities, if we compare **9f** with **9i** with 1.22 µM and 1.88 µM, respectively.

**TABLE 4:** SAR exploration of quinazoline.

Compound	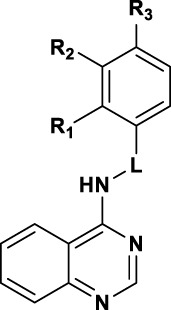				HCC1395 TopFlash^a^		HCC1395 Cytotoxicity^b^	
	L	R_1_	R_2_	R_3_	IC_50_ (µM)	Efficacy (%)	IC_50_ (µM)	Efficacy (%)
9a	-CH_2_-	OMe	H	H			29.03 ± 14.51	100 ± 0
9b	-CH_2_-	H	OMe	H	3.58 ± 1.21	82.6 ± 13.5	>50	
9c	-CH_2_-	H	H	OMe	4.76 ± 0.23	81.5 ± 10.61	>50	
9d	-CH_2_-	H	H	F	0.67 ± 0.13	87.1 ± 4.09	>50	
9e	-CH_2_-	H	H	Cl	2.18 ± 0.85	96.0 ± 5.7	>50	
9f	-CH_2_-	H	H	Me	1.22 ± 0.33	51.6 ± 7.9	8.13 ± 3.8	100 ± 0
9g	-(CH_2_)_2_-	H	H	Cl			13.02 ± 1.45	100 ± 0
9h	-CH(Me)-	H	H	F	10.70 ± 2.33	66.4 ± 6.9	16.60 ± 6.59	51.1 ± 21.3
9i	-CH(Me)-	H	H	Me	1.88 ± 0.28	54.6 ± 2.9	10.33 ± 2.06	38.7 ± 12.8

^a^ Stimulation of the Wnt-pathway with CHIR99021 (GSK3β inhibitor) and inhibition of the Wnt-pathway is measured by the TopFlash assay.

^b^ Cytotoxic activity measured by Renilla luciferase.

In general, this investigation of SAR of the three areas of the hit compound led to up to 10-fold improvements from the initial compound **4a** (IC_50_ = 8.31 µM) to compound **9d** with IC_50_ of 0.67 µM.

### 2.4 Translation in cancer models

#### 2.4.1 Inhibition of Wnt signaling translates in the decrease of cell proliferation in a panel of TNBC cell lines

**FIGURE 3:**
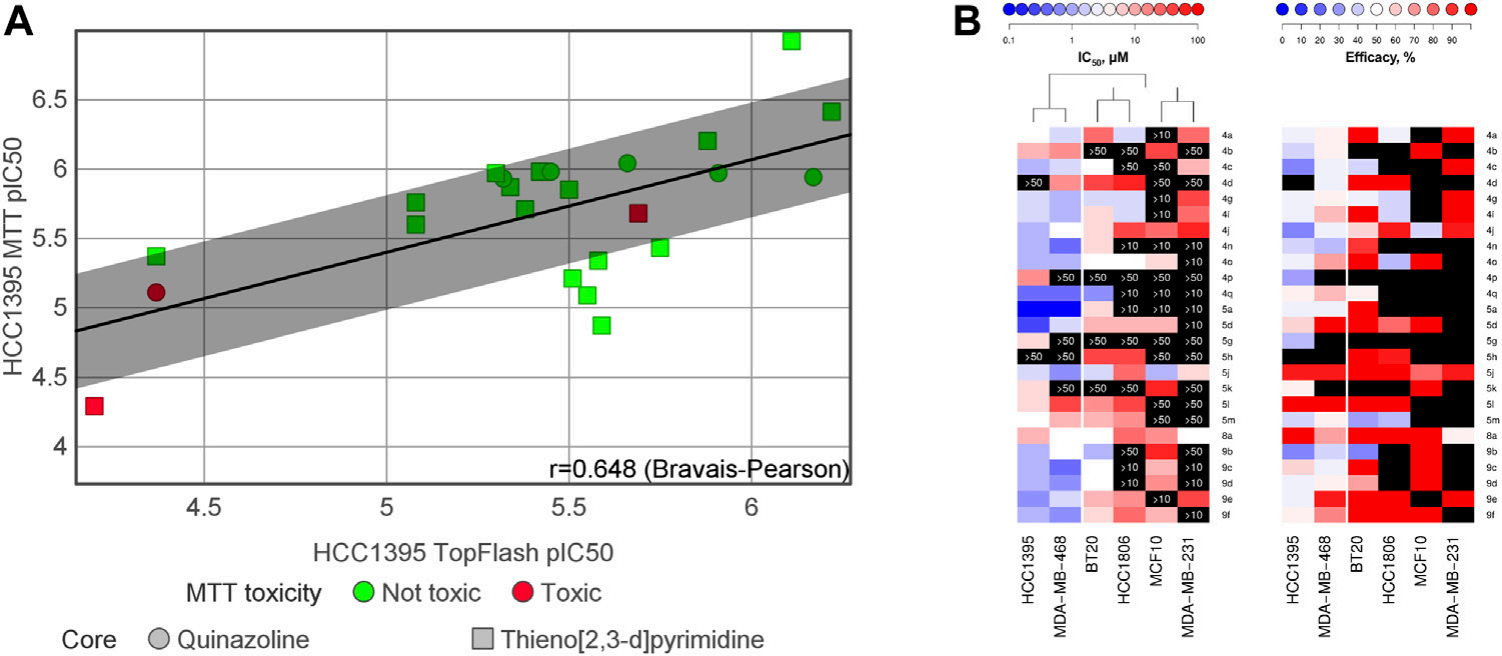
**(A)** Correlation between inhibition of the Wnt-pathway (measured by the TopFlash assay) and inhibition of TNBC cell proliferation (measured by the MTT assay). “MTT toxicity” refers the ratio between the cell proliferation IC_50_ measured on MCF10a (non-cancerous breast cells) by the IC_50_ measured on HCC1395 (TNBC cells); “Not toxic” refers to this ratio being less than 3-fold. **(B)** Heatmaps summarizing IC_50_ and efficacy of the compounds against diverse TNBC cell lines and the control non-cancerous MCF10a cell line. Only compounds that had previously demonstrated a specific anti-Wnt activity in HCC1395 cells were tested; black blocks indicate no activity within the tested concentration range, with the maximal tested concentration indicated on the block. HCC1395 and MDA-MB-468 cell lines compose a distinct cluster with overall higher susceptibility to the compounds, whereas the other tested TNBC cell lines cluster together with MCF10a cells, demonstrating lower responses.

To assess the translation of the compounds’ anti-Wnt potency—ranging from two-digits micromolar to submicromolar values—into inhibition of cell proliferation, we applied the classical MTT assay. As illustrated in Figure 3A, there was a good correlation for HCC1395 cells between the TopFlash and MTT assays with r = 0.648. Interestingly, the partial efficacy observed for some compounds in the TopFlash assay appeared not to influence the potency in the MTT survival assay. We further evaluated whether our compounds could inhibit proliferation of a large panel of TNBC cell lines and of the non-cancerous breast fibrocystic MCF10a cells (Figure 3B and Supplementary Table S2). We analyzed the obtained set of IC_50_’s by independent clustering that resulted in a clear separation of the cell lines tested into 2 groups: highly sensitive ones (HCC1395 and MDA-MB-468 lines) and poorly sensitive ones (BT20, HCC1806 and MDA-MB-231). Cell lines with high susceptibility to the compounds’ treatment systematically demonstrated lower IC_50_s as compared to the others. Those with the low antiproliferative sensitivity also clustered with the control non-cancerous MCF10a cells, which indicates strong selectivity of the compounds towards a subset cancer cells. In this regard, only few compounds such as **5j**, **5h** and **8a** (Figure 3A in red, see Supplementary Table S2) were considered to have promiscuous effects with no selectivity over the control cells (less than 3-fold difference in IC_50_), although **5h** should rather be considered as inactive (IC_50_ > 50 µM).

#### 2.4.2 High-potency compounds suppress expression of Wnt target gene c-Myc in HCC1395 cells and are selective for the Wnt-pathway

**FIGURE 4:**
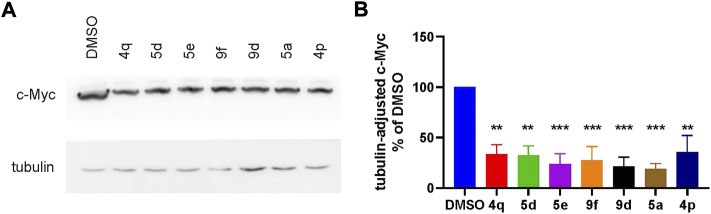
**(A)** Representative Western blot and **(B)** quantification of suppression of the Wnt target gene c-Myc by high-potent compounds (taken at 5 µM). Statistical significance was assessed by one-way ANOVA and is shown as **p < 0.01, ***p < 0.001.

We continued by analysis of the compounds’ capacity to suppress Wnt signaling in TNBC cells independently of an external Wnt ligand and beyond the artificial TopFlash reporter system. To this end, we assessed the expression levels of the transcription factor c-Myc, a well-proven key target gene of the pathway (Rennoll and Yochum, 2015; Koval et al., 2018; Ahmed et al., 2019). Effects of the compounds on c-Myc levels in HCC1395 cells upon treatment by the indicated compounds, chosen based on their higher potency (IC_50_ between 0.6 µM and 2.59 µM in the TopFlash assay), are summarized in Figure 4. As expected, Wnt inhibition by the compounds resulted in profoundly decreased endogenous c-Myc levels.

**FIGURE 5:**
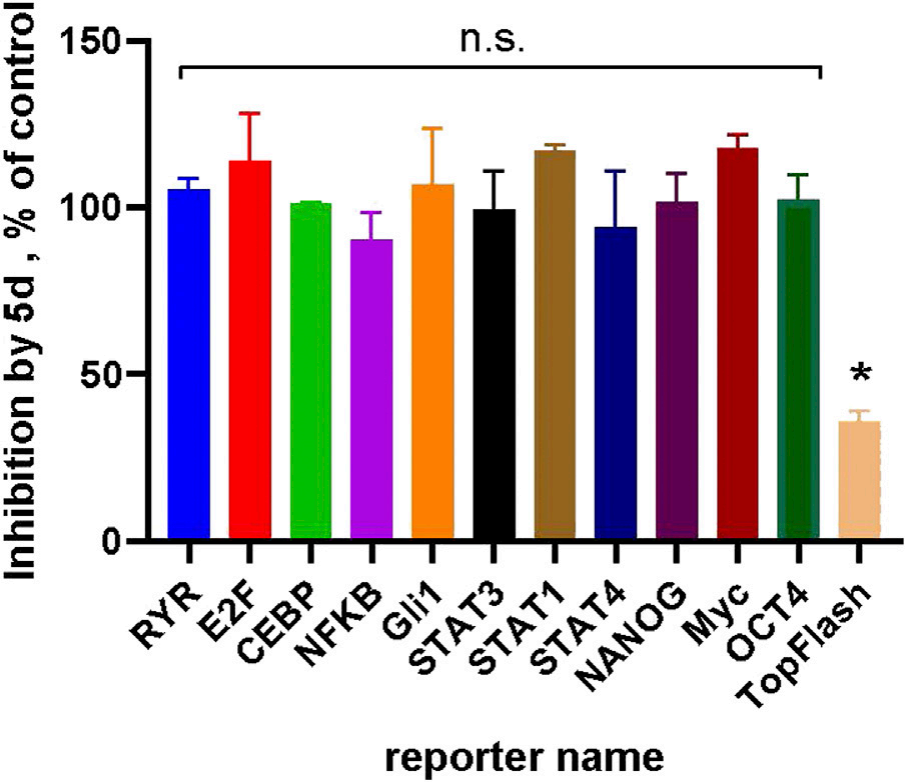
Compound 5d (at 5 µM) demonstrates selectivity towards the Wnt-pathway: no inhibition in a panel of luciferase reporters with measurable basal activities in HCC1395 cells could be observed by the compound. Inhibition of the Wnt3a-stimulated TopFlash signal is shown as a positive control. Statistical significance was assessed by one-sample *t*-test and is shown as *p < 0.05; “n.s.”—not significant.

Compound **5d** (IC_50_ = 0.6 µM, Table 2) was used as a representative to analyze the selectivity of the chemotype towards the Wnt-pathway, by profiling it against a panel of different transcriptional reporters with significant basal activities in HCC1395 cells (Figure 5). These results demonstrate that none other signaling pathways are affected by the compound. We thus conclude that the anti-proliferative effect of the compound is related exclusively to the suppressed Wnt signaling.

#### 2.4.3 Gene expression analysis points towards differences in the Wnt-pathway organization in cell lines with different sensitivity to the compound

**FIGURE 6:**
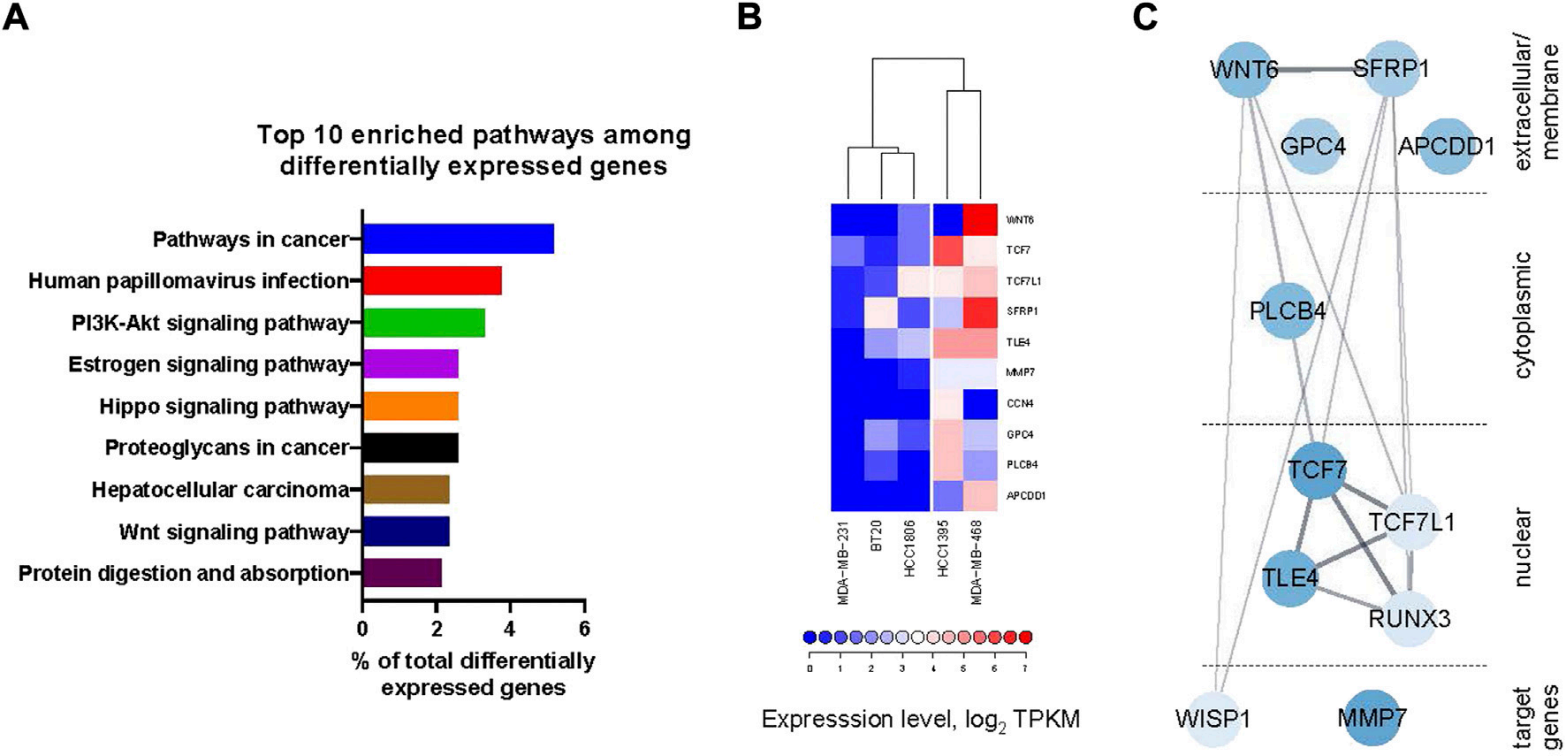
**(A)** Top 10 enriched pathways (by DAVID online tool) among the genes differentially expressed between the lines highly sensitive to the compound treatment (HCC1395 and MDA-MB-468) and those with poor sensitivity (HCC 1806, BT20 and MDA-MB-231) (see also Supplementary Table S3) includes Wnt-pathway. **(B)** Heatmap of the expression levels of differentially expressed Wnt-pathway component and target genes with clustering clearly delineates the cell lines sensitive to the compound treatment. **(C)** Representation of the differentially expressed Wnt-related genes as a network with physical and genetic interactions overlaid from StringDB. Intensity of coloration is proportional to the overall difference in the expression levels between the sensitive and insensitive lines.

The fact that some TNBC cell lines were highly sensitive to the compounds, while others were much less so (see Figure 3), brought us to hypothesize that different gene expression determinants existed between these two sets of cancer lines. We took advantage of the RNAseq-derived gene expression datasets in CCLE (Cancer Cell Line Encyclopedia (Barretina et al., 2012)) available for the five TNBC lines used in our studies. In order to pinpoint the genes most likely responsible for the differential response among the two groups of cell lines, we extracted 419 genes that had at least 4-fold higher or lower average expression levels in the HCC1395 and MDA-MB-468 lines as compared to the other cell lines. Pathway enrichment analysis of this list performed with the DAVID online tool (Huang et al., 2009; Sherman et al., 2022) identified the Wnt-pathway among the top over-represented pathways in this list, with 10 genes (Figure 6A and Supplementary Table S3). Indeed, expression levels of these 10 Wnt component and target genes cluster the 5 TNBC cell lines into the same two groups as by the potency to the compound treatment (Figure 6B). Plotting StringDB (Szklarczyk et al., 2021) interactions (physical and genetic) among these genes place them in a tightly interconnected network (Figure 6C). Taken together, these data indicate that the differential capacity of the compounds to inhibit proliferation of these lines is driven by peculiarities of the Wnt-pathway organization in them—the phenomenon previously predicted by us (Koval and Katanaev, 2018).

#### 2.4.4 Anti-Wnt activity of a panel of compounds synergizes with the chemotherapy agent docetaxel

**FIGURE 7:**
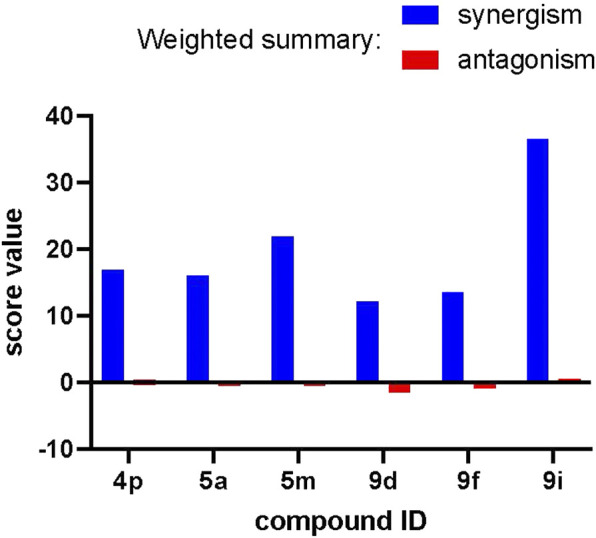
Strong synergism between our compounds and docetaxel in the anti-proliferative assays. Weighted summary synergism and antagonism scores obtained for different compounds after analysis with the COMBENEFIT software are presented.

The Wnt-pathway is implicated in chemoresistance to conventional chemotherapies and drives a poor chemotherapy response in a broad range of tumors (O’Reilly et al., 2015; Martin-Orozco et al., 2019; Zhong and Virshup, 2020). To assess how combination with our compounds affects the anti-proliferative capacity of docetaxel—one of the first-line treatments for TNBC (Mandapati and Lukong, 2022), we performed the Loewe synergism assessment over a complete dose-response range of both compounds followed by analysis in the COMBENEFIT software in Matlab (Figure 7 and Supplementary Figure S1) (Di Veroli et al., 2016).

The weighted summary synergism and antagonism scores indicate that compounds **4p**, **5a**, **5m**, **9d**, **9f**, and **9i** exert exclusively synergistic effects when used as a co-treatment with docetaxel. This effect is overall similar among the compounds (best for **9i**) and can be characterized as mild since it scores 15–30 on a scale of 0–100. The dose-by-dose matrices calculated for each compound presented at Supplementary Figure S1 indicate that for most of the compounds, major zones of synergism can be seen at the highest concentrations of our compounds and intermediate concentrations of docetaxel. This serves as a strong indication that application of these compounds *in vivo* might be useful in combination with a conventional chemotherapy through reduction of the chemotherapy dose necessary to obtain a complete response.

## 3 Discussion

From the initial thienopyrimidine hit with an IC_50_ of 8.31 µM, SAR exploration of three portions of the scaffold led to a 10-fold improvement with four compounds active below the µM range in HCC1395 cells. Although further SAR exploration would be necessary to fully understand the properties driving potency in the HCC1395 cell-based assay, elements of SAR were identified. From exploration of the top part (Table 1, Table 2 and Table 4), electron density of the phenyl does not seem to be critical as electron withdrawing or donating groups have little influence on the potency. Lipophilic or polar interactions are more important in this region. Also, for the thieno [2,3-*d*]pyrimidine derivatives, the most potent compounds are 1-phenylethylamine analogues (compounds **5a**, **5d** and **5e**; Table 2), of which only racemic mixture were tested. It would be interesting to prepare and test each enantiomer. For the core modifications, a better activity was achieved with the quinazoline nucleus replacing the thienopyrimidine core. We also observed some differences between thieno [2,3-*d*]pyrimidine and quinazoline SAR (Table 1 and Table 4). Although we expected a good shape similarity for both cores, we could speculate that the subtle electron density difference resulting from the bioisosteric replacement of the thiophene part by phenyl affects the interaction with the biological target.

Based on the assay paradigm, we could conclude that these compounds act below the destruction complex within the Wnt-pathway, as they inhibited the β-catenin-dependent signaling induced by an inhibitor of GSK3β, CHIR99021. Moreover, our exploration of the genetic differences between the cancer lines with strong and weak proliferative response to the derivatives highlighted a set of the Wnt signaling component genes likely responsible for this effect, where TCF7 (a.k.a TCF-1) and TCF7L1 (a.k.a TCF-3) and their immediate regulators and co-factors TLE4 and RUNX3 occupy the central place. Taken together with a significant evidence of non-redundant relationships among TCF/LEF transcriptional factors (Arce et al., 2006; Mao and Byers, 2011), it beckons us that the molecular target of our compounds might be hidden among unique partners of these transcriptional factors. This proposition is also reinforced by the clear specificity of action for our compounds to Wnt signaling with no significant inhibition of other signal transduction pathway observed in the HCC1395 cell line.

The search for such factors—molecular targets of our compounds—is essential for further development of this promising series of compounds. The selective mechanism of action of our compounds might be a double-edged sword: while it is likely behind their capacity to avoid strong effects on healthy cells represented by MCF-10a in our study, on the other hand it might also limit the scope of therapeutic applications of the compounds. With proliferation of 2 out of 5 TNBC lines strongly affected by the compounds, deciphering of the molecular target and of the full mechanism of action will be a key for evaluation of their scope within TNBC and other cancers, as well as for eventual patient stratification for the application of this novel therapy. Immortalized cancer cell lines as well as 2D cultures have certain limitations in translation of the results obtained to a more complex 3D milieu of the tumor in the patients. However, our findings represent a promising step forward in the development of the long-desired Wnt-targeting agents. Future investigations will follow the logical steps of the preclinical development using animal models, and should further provide interesting insights into the roles of Wnt signaling in the tumor resistance, given the synergistic relationships between our compounds and the conventional chemotherapeutic docetaxel.

## 4 Conclusion

From the initial potency of the thienopyrimidine **4a** with IC_50_ of 8.31 µM on the HCC1395 cell line, investigation of three portions of the molecule to generate a library of compounds led to identification of submicromolar inhibitors. Through orthogonal readouts, we confirmed that these thienopyrimidine derivative compounds act as downstream inhibitors of the β-catenin-dependent Wnt-pathway. Targeting downstream components would be more efficacious for the tumors where the pathway is induced by loss-of-function mutations in APC or Axin or by gain-of-function mutations in β-catenin or TCF, as compared to the upstream pathway inhibitors such as Porcupine inhibitors. Inhibition of the Wnt-pathway is translated into differential efficacy on inhibition of TNBC cell proliferation. Although we have not yet identified the exact molecular target, our analyses indicate the target to be among the partners of β-catenin-dependent transcriptional factors. Identification of the molecular target would unlock further development through the target-based assay to support further SAR exploration to identify compounds suitable for *in vivo* evaluation. Such findings will also be key to define the scope of application, determine potential biomarkers, and permit patient stratification.

## 5 Material and methods

### 5.1 Compound potency in TopFlash and other luciferase reporter assays

The Wnt3a or CHIR99021-stimulated luciferase activity in HCC1395 TNBC cell line stably transfected with the TopFlash reporter was analyzed essentially as described for the BT-20 TNBC cell line (Shaw et al., 2019b; Koval et al., 2021). Reporter cells were seeded at 10,000 cells/well in a white opaque 384-well plate in the final volume of 20 μl of DMEM medium supplemented with 10% FCS. The cells were maintained incubated at 37°C, 5% CO_2_ overnight for attachment. Subsequently, they were transfected by a plasmid encoding constitutively expressed Renilla luciferase under the CMV promoter overall as described in the manufacturer’s protocol using 12 μg/ml of DNA and 40 μl/ml XtremeGENE nine reagent. The next day, the medium in each well was replaced with a 10 μl of fresh medium containing compound of interest, and, after 1 h of preincubation with the compounds, additional 10 μl of medium supplemented with Wnt3a [purified as described (Willert et al., 2003; Koval and Katanaev, 2011)] or CHIR99021 were added creating respective final concentrations of 2.5 μg/ml or 5 μM of each. In case of other signal transduction reporters, no additional stimulation was performed. Compound dilutions were prepared by serial dilution in DMSO and diluted with the amount of medium necessary to obtain their final concentrations indicated on the figures and tables and maintain concentration of DMSO of 0.5% in all assay points. After overnight incubation, the supernatant in each well was removed, and the luciferase activity was measured as described (Dyer et al., 2000; Huber et al., 2021; Pellissier et al., 2021). The culture medium was completely removed from all wells of the plate. Finally, the luciferase activity of the firefly and Renilla luciferases was detected sequentially in individual wells of a 384-well plate through injection of corresponding measurement solutions and immediate reading (400 ms integration time) in Infinite M Plex multifunctional plate reader with injection module.

### 5.2 Survival assay

Indicated TNBC cell lines were detached and resuspended at 120,000 cells/ml and added into each well of a transparent 384-well plate in the final volume of 20 µl/well. The cells were maintained in DMEM containing 10% FBS at 37°C, 5% CO_2_ overnight. Next day, the medium in each well was replaced by 40 μl of the fresh one containing the indicated concentrations of compounds. In case of drug combination experiments, each compound dilution was delivered in corresponding well in 20 μl of medium. Compound dilutions were prepared by serial dilution in DMSO and diluted with the amount of medium necessary to obtain their final concentrations indicated on the figures and tables and maintain concentration of DMSO of 0.5% in all assay points. After incubation for 3–4 days, depending on the cell line, the medium in each well was replaced by 25 μl of 0.5 mg/ml Thiazolyl blue solution in 1xPBS following by incubation for 3 h at 37°C. Then the solution was removed and 25 μl DMSO was added into each well. Absorbance at 510 nm was measured in a Tecan Infinite M200 PRO plate reader.

### 5.3 Western blotting

HCC1395 TNBC cell line was seeded at 100,000 cells/well in 24 well plates. On the next day, the medium was replaced by the fresh one pre-warmed at 37°C containing the indicated compounds. After 24 h incubation, the medium was removed, followed by washing twice with 500 μl of 1x PBS. The cells were lysed in the well by addition of 30 μl of ice-cold RIPA buffer (1x TBS, 4 mM EDTA, 1% Triton, 0.1% SDS, 1x Protease inhibitor cocktail (Roche)) and incubated on ice for 10 min. The samples were resuspended and then centrifuged at 18,000 g at 4°C to remove debris. 15 μl of the supernatants each were further analyzed by Western blot with antibodies against c-Myc (Abcam) and α-Tubulin (Sigma) at 1:1,000 dilutions.

### 5.4 Data analysis and sources

The data was visualized analyzed in either GraphPad Prism nine using indicated statistical analysis or in R using gplots package (Warnes et al., 2009). Dataset from CCLE (Barretina et al., 2012) were used in the study and pathway enrichment analysis was performed by DAVID online tool (Sherman et al., 2022). Drug combination data was treated in COMBENEFIT software (Di Veroli et al., 2016). Network was built using Cytoscape with StringDB plugin (Shannon et al., 2003; Szklarczyk et al., 2021).

## Data Availability

The original contributions presented in the study are included in the article/Supplementary Material, further inquiries can be directed to the corresponding author.
