# Erratum: Wnt-pathway inhibitors with selective activity against triple-negative breast cancer: From thienopyrimidine to quinazoline inhibitors

**DOI:** 10.3389/fphar.2023.1173490

**Published:** 2023-03-07

**Authors:** Frontiers Production Office

**Affiliations:** Frontiers Media SA, Lausanne, Switzerland

**Keywords:** Wnt signaling, triple-negative breast cancer, β-catenin, cancer survival, medicinal chemistry, structure activity relationship, thienopyrimidine, quinazoline

An omission to the **Funding** section of the original article was made in error. The following sentence has been added: “Open access funding was provided by the University of Geneva.”

The original version of this article has been updated.

