# Supplementary figures and images for: Wnt-pathway inhibitors with selective activity against triple-negative breast cancer: From thienopyrimidine to quinazoline inhibitors
Source: Front Pharmacol. 2022 Oct 28;13:1045102. doi: 10.3389/fphar.2022.1045102 (PMC9649909; doi:10.3389/fphar.2022.1045102)

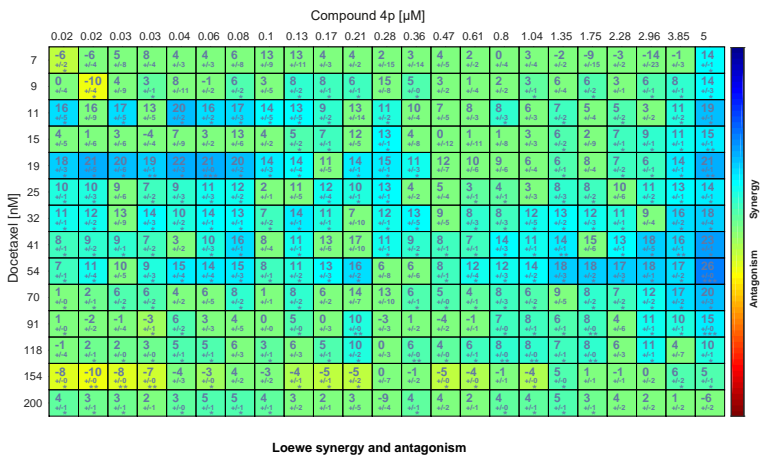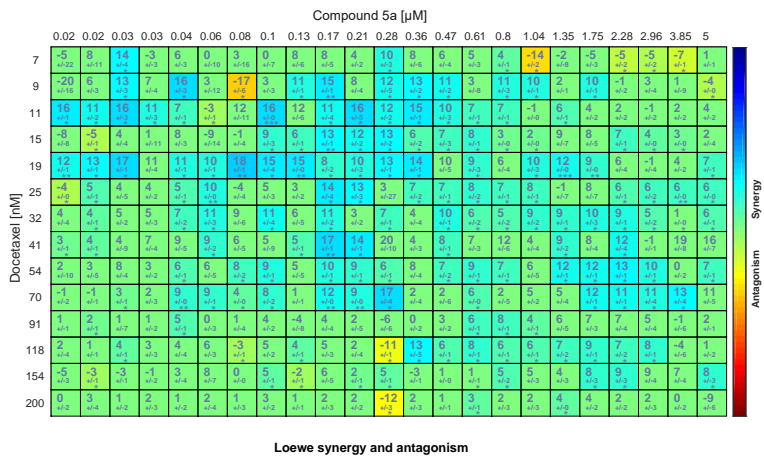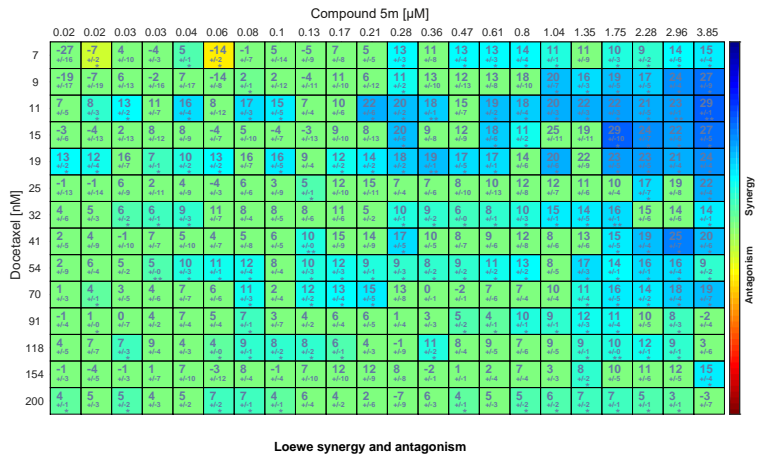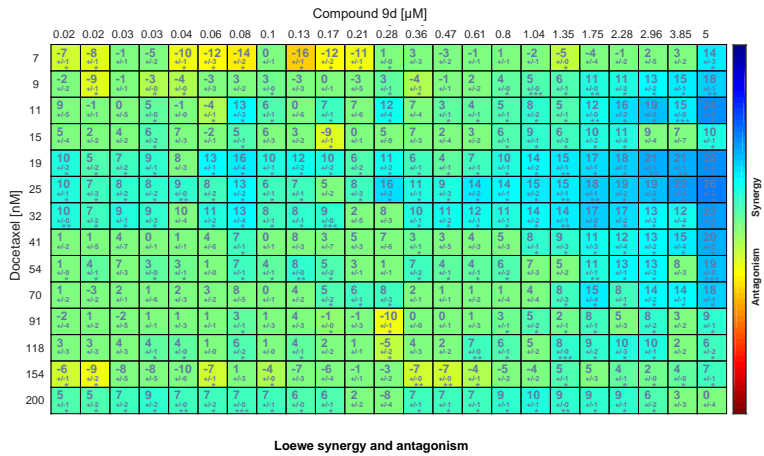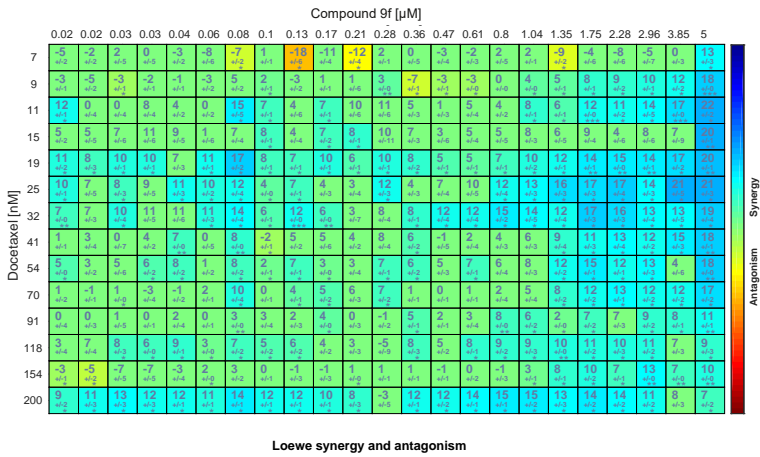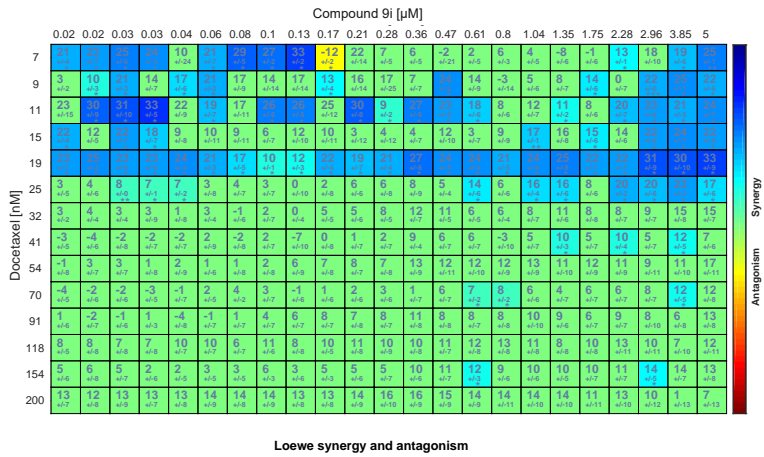

Supplement: Supplementary file 2 [file DataSheet1.PDF]
