# Supplementary material for: Wnt-pathway inhibitors with selective activity against triple-negative breast cancer: From thienopyrimidine to quinazoline inhibitors
Source: Front Pharmacol. 2022 Oct 28;13:1045102. doi: 10.3389/fphar.2022.1045102 (PMC9649909; doi:10.3389/fphar.2022.1045102)
Supplement: Supplementary file 3 [file DataSheet2.docx]

Supplementary Material

# Table S1 | SAR exploration to replace thieno[2,3-d]pyrimidine

|  |  | **HCC1395 TopFlash^a^** | | **HCC1395 Cytotoxicity^b^** | |
| --- | --- | --- | --- | --- | --- |
| **Compound** | **Ar** | **IC_50_ (µM)** | **efficacy (%)** | **IC_50_ (µM)** | **efficacy (%)** |
| **4c** |  | 4.96 ± 1.65 | 58.6 ± 22.8 | >100 |  |
| **9b** |  | 42.8 ± 10.57 | 100 ± 0 | ND |  |
| **8a** |  | 3.58 ± 1.21 | 82.6 ± 13.5 | >100 |  |
| **10** |  | >100 |  | >100 |  |
| **11** |  | 58.24 ± 1.48 | 100 ± 0 |  |  |
| **12** |  | >100 |  | ND |  |
| **13** |  | >100 |  | >100 |  |
| **16** |  |  |  | 18.67 ± 3.93 | 39.4 ± 13.0 |

^a^ Stimulation of the Wnt-pathway with CHIR99021, a GSK3ß inhibitor and inhibition of Wnt-pathway is measured by TopFlash assay; ^b^ Cytotoxic activity measured by Renilla luciferase.

# Compound synthetic procedures and characterization

Reactions were carried out under a positive argon atmosphere unless otherwise stated. Anhydrous solvents were kept over 4Å molecular sieves. Solvents of analytical grade were used for purifications and reaction treatments. Chromatographic separations were performed on an automated Büchi Laboratory® C-815 Flash apparatus equipped with UV detector and using a pre-packed silica gel column (FlashPure; 35-45 µm). A Bruker® Avance III HD 600 MHz NMR spectrometer equipped with a QCI 5mm Cryoprobe and a SampleJet automated sample changer (Bruker® BioSpin, Rheinstetten, Germany) was used to perform 1H and 13C NMR experiments. All chemical shifts are expressed in parts per million (ppm) relative to the peak of chloroform ( Chloroform*-d*; 7.26 ppm for 1H; 77.16 ppm for 13C), or deuterium methanol (MeOD; 3.31 ppm for 1H; 49.00 ppm for 13C), or dimethyl sulfoxide (DMSO; 2.50 ppm for 1H; 39.52 ppm for 13C). The protons peaks are described according to NMR conventions: “s” for singlet, “br” for broad singlet, “d” for doublet, “t” for triplet, and “q” for quadruplet. The carbon peaks are described using DEPTQ experiment which results in positive pics for quaternary “C” and “CH_2_” carbons and negative pics for “CH” and “CH_3_”. Unless stated otherwise carbon pics are assigned for one carbon. Chemical shifts are in ppm and constant coupling are expressed in Hz. The device used to record mass spectra was an Advion Expression® CMS operating in electrospray positive and negative mode (ESI) simultaneously.

## General procedure A: library exploration at position 4 of thieno[2,3*-d*]pyrimidine

To the solution of 4-chlorothieno[2,3*-d*]pyrimidine (1 eq) in dry DMF (0.145 M), the corresponding amine (1.1 eq.) and diisopropylethylamine (1.15 eq) were added and the mixture was stirred at 100°C for 4 h. The mixture was cooled to rt and quenched with water. The aqueous layer was extracted three times with ethyl acetate. The combined organic layers were washed with brine twice, dried over anhydrous MgSO_4_, filtered and evaporated. The crude mixture was purified on column chromatography on silica gel to afford the desired compound.

***N-*benzylthieno[2,3*-d*]pyrimidin-4-amine, 4a**

The titled compound **4a** was prepared according to General Procedure A, using benzylamine (35 µL, 0.32 mmol, 1.1 eq) as corresponding amine. The crude mixture was purified by column chromatography on silica gel using as eluent a gradient of CyHex/AcOEt from 1:1 to 2:3 to afford the compound **4a** as a white off solid (36 mg, 50%). m/z for C_13_H_11_N_3_S = 241.07 (calculated), 242.0 (found, [M+H]^+^). ^1^H NMR (600 MHz, Chloroform*-d*) δ 8.67 (s, 1H), 7.71 (d, J = 5.3 Hz, 1H), 7.44 (d, J = 5.4 Hz, 1H), 7.42 – 7.30 (m, 5H), 5.15 (s, 1H), 4.88 (d, J = 5.6 Hz, 2H). ^13^C NMR (151 MHz, Chloroform*-d*) δ 160.2 (C), 157.3 (C), 155.3 (CH), 138.3 (C), 131.0 (CH), 129.0 (CH, 2C), 128.1 (2C, CH), 127.9 (CH), 125.7 (CH), 115.2 (C), 45.3 (CH_2_).

***N-*(2-methoxybenzyl)thieno[2,3*-d*]pyrimidin-4-amine, 4b**

The titled compound **4b** was prepared according to General Procedure A, using 2-methoxybenzylamine (59 µL, 0.45 mmol, 1.1 eq) as corresponding amine. The crude mixture was purified on silica gel using as eluent a gradient of CyHex/AcOEt from 1:1 to 2:3 to afford the compound **4b** as a white off solid (69 mg, 62%). m/z for C_14_H_13_N_3_OS = 271.08 (calculated), 272.0 (found, [M+H]^+^). ^1^H NMR (600 MHz, Chloroform*-d*) 8.65 (s, 1H), 7.68 (d, J = 5.3 Hz, 1H), 7.41 (d, J = 5.3 Hz, 1H), 7.38 (dd, J = 7.4, 1.7 Hz, 1H), 7.29 (td, J = 7.8, 1.7 Hz, 1H), 6.96 – 6.91 (m, 2H), 5.43 (d, J = 6.1 Hz, 1H), 4.87 (d, J = 5.8 Hz, 2H), 3.90 (s, 3H). ^13^C NMR (151 MHz, Chloroform*-d*) δ 159.93 (C), 157.86 (C, 2C), 157.43 (C), 155.26 (CH), 130.76 (CH), 130.01 (CH), 129.24 (CH), 126.22 (C), 125.61 (CH), 120.86 (CH), 110.63 (CH), 55.55 (CH_3_), 41.17 (CH_2_).

***N-*(3-methoxybenzyl)thieno[2,3*-d*]pyrimidin-4-amine, 4c**

The titled compound **4c** was prepared according to General Procedure A, using 3-methoxybenzylamine (58 µL, 0.45 mmol, 1.1 eq) as corresponding amine. The crude mixture was purified by column chromatography on silica gel using as eluent a gradient of CyHex/AcOEt from 1:1 to 2:3 to afford the compound **4c** as a white off solid (62 mg, 55%). m/z calculated for C_14_H_13_N_3_OS = 271.08 (calculated), 272.0 (found, [M+H]^+^). ^1^H-NMR (600 MHz, Chloroform*-d*) δ 8.67 (s, 1H), 7.71 (d, J = 5.4 Hz, 1H), 7.44 (d, J = 5.3 Hz, 1H), 7.29 (t, J = 7.9 Hz, 1H), 6.98 (ddd, J = 7.5, 1.6, 0.9 Hz, 1H), 6.95 (t, J = 2.1 Hz, 1H), 6.85 (ddd, J = 8.3, 2.6, 0.9 Hz, 1H), 5.18 (t, J = 5.7 Hz, 1H), 4.85 (d, J = 5.6 Hz, 2H), 3.80 (s, 3H). ^13^C-NMR (151 MHz, Chloroform*-d*) δ 160.15 (C), 160.13 (C), 157.28 (C), 155.25 (CH), 139.87 (C), 131.05 (CH), 130.05 (CH), 125.66 (CH), 120.22 (CH), 115.25 (C), 113.76 (CH), 113.17 (CH), 55.41 (CH_3_), 45.23 (CH_2_).

***N-*(4-methoxybenzyl)thieno[2,3*-d*]pyrimidin-4-amine, 4d**

The titled compound **4d** was prepared according to General Procedure A, using 4-methoxybenzylamine (57 µL, 0.45 mmol, 1.1 eq) as corresponding amine. The crude mixture was purified by column chromatography on silica gel using as eluent a gradient of CyHex/AcOEt from 1:1 to 2:3 to afford the compound **4d** as a white off solid (51 mg, 45%). m/z for C_14_H_13_N_3_OS = 271.08 (calculated), 272.0 (found, [M+H]^+^). ^1^H NMR (600 MHz, Chloroform*-d*) δ 8.67 (s, 1H), 7.70 (d, J = 5.4 Hz, 1H), 7.43 (d, J = 5.3 Hz, 1H), 7.35 – 7.31 (m, 2H), 6.92 – 6.88 (m, 2H), 5.10 (t, J = 5.4 Hz, 1H), 4.80 (d, J = 5.4 Hz, 2H), 3.81 (s, 3H). ^13^C NMR (151 MHz, Chloroform*-d*) δ 160.07 (C), 159.42 (C), 157.22 (C), 155.25 (CH), 130.99 (CH), 130.25 (C), 129.48 (CH, 2C), 125.64 (CH), 115.23 (C), 114.37 (2C, CH), 55.48 (CH_3_), 44.84 (CH_2_).

***N-*(2-fluorobenzyl)thieno[2,3*-d*]pyrimidin-4-amine, 4e**

The titled compound **4e** was prepared according to General Procedure A, using (2-fluorophenyl)methanamine (52 µL, 0.45 mmol, 1.1 eq) as corresponding amine. The crude mixture was purified by column chromatography on silica gel using as eluent a mixture of CyHex/AcOEt, 1:1 to afford the compound **4e** as pale yellow solid (61 mg, 57%). m/z for C_13_H_10_FN_3_S = 259.06 (calculated), 260.0 (found, [M+H]^+^). ^1^H-NMR (600 MHz, Chloroform*-d*) δ 8.66 (s, 1H), 7.71 (d, J = 5.3 Hz, 1H), 7.45 (td, J = 7.6, 1.8 Hz, 1H), 7.43 (d, J = 5.4 Hz, 1H), 7.31 – 7.26 (m, 1H), 7.12 (td, J = 7.6, 1.2 Hz, 1H), 7.10 – 7.06 (m, 1H), 5.33 (bs, 1H), 4.94 (d, J = 5.9 Hz, 2H). ^13^C-NMR (151 MHz, Chloroform*-d*) δ 161.30 (C, d, J = 246.0 Hz), 159.98 (C), 157.26 (C), 155.08 (CH), 131.13 (CH) 130.44 (CH, d, J = 3.0 Hz), 129.64 (CH, d, J = 9.0 Hz), 125.56 (CH), 125.31 (C, d, J = 6.8 Hz), 124.48 (CH, J = 4.5), 115.66 (CH, d, J = 21.1 Hz), 115.35 (C), 39.13 (CH_2_, d, J = 3.0 Hz).

***N-*(4-fluorobenzyl)thieno[2,3*-d*]pyrimidin-4-amine, 4g**

The titled compound **4g** was prepared according to General Procedure A, using (4-fluorophenyl)methanamine (52 µL, 0.45 mmol, 1.1 eq) as corresponding amine. The crude mixture was purified by column chromatography on silica gel using as eluent a gradient of a mixture of CyHex/AcOEt 1:1 to afford the compound **4g** as brownish solid (73 mg, 68%). m/z for C_13_H_10_FN_3_S = 259.06 (calculated), 260.0 (found, [M+H]^+^). ^1^H-NMR (600 MHz, Chloroform*-d*) δ 8.66 (s, 1H), 7.71 (dd, J = 5.4, 0.9 Hz, 1H), 7.44 (dd, J = 5.4, 0.9 Hz, 1H), 7.40 – 7.33 (m, 2H), 7.06 – 7.02 (m, 2H), 5.27 (t, J = 5.7 Hz, 1H), 4.85 (d, J = 5.7 Hz, 2H). ^13^C-NMR (151 MHz, Chloroform*-d*) δ 163.29 (C), 160.84 (C, d, J = 246.0 Hz), 157.23 (C), 155.13 (CH), 134.09 (C, d, J = 3.0 Hz), 131.18 (CH), 129.70 (CH, d, J = 9.1 Hz, 2C), 125.61 (CH), 115.82 (CH, J = 21.1 Hz, 2C), 115.26 (C), 44.49 (CH_2_).

***N-*(2-chlorobenzyl)thieno[2,3*-d*]pyrimidin-4-amine, 4h**

The titled compound **4h** was prepared according to General Procedure A, using (2-chlorophenyl)methanamine (55 µL, 0.45 mmol, 1.1 eq) as corresponding amine. The crude mixture was purified by column chromatography on silica gel using as eluent a gradient of CyHex/AcOEt 1:1 and further purified by column chromatography using as eluent a mixture of DCM/AcOEt, 95:5 to afford the compound **4h** as white solid (75 mg, 66%). m/z for C_13_H_10_ClN_3_S = 275.03 (calculated), 276.5 (found, [M+H]^+^). ^1^H-NMR (600 MHz, Chloroform*-d*) δ 8.66 (s, 1H), 7.71 (dd, J = 5.4, 0.8 Hz, 1H), 7.50 – 7.47 (m, 1H), 7.43 (dd, J = 5.4, 0.9 Hz, 1H), 7.41 – 7.38 (m, 1H), 7.26 – 7.23 (m, 2H), 5.40 (t, J = 6.2 Hz, 1H), 4.98 (d, J = 6.0 Hz, 2H). ^13^C-NMR (151 MHz, Chloroform*-d*) δ 159.98 (C), 157.27 (C), 155.07 (CH), 135.70 (C), 133.88 (C), 131.19 (CH), 130.28 (CH), 129.81 (CH), 129.22 (CH), 127.22 (CH), 125.55 (CH), 115.36 (C), 43.03 (CH_2_).

***N-*(3-chlorobenzyl)thieno[2,3*-d*]pyrimidin-4-amine, 4i**

The titled compound **4i** was prepared according to General Procedure A, using (3-chlorophenyl)methanamine (55 µL, 0.45 mmol, 1.1 eq) as corresponding amine. The crude mixture was purified by column chromatography on silica gel using as eluent a mixture of CyHex/AcOEt, 1:1 to afford the compound **4i** as white solid (64 mg, 56%). m/z for C_13_H_10_ClN_3_S = 275.03 (calculated), 276.0 (found, [M+H]^+^), 298.1 (found, [M+Na]^+^). ^1^H-NMR (600 MHz, Chloroform*-d*) δ 8.66 (s, 1H), 7.72 (d, J = 5.4 Hz, 1H), 7.44 (d, J = 5.4 Hz, 1H), 7.38 (d, J = 2.1 Hz, 1H), 7.28 (d, J = 3.3 Hz, 3H), 5.36 (t, J = 5.6 Hz, 1H), 4.87 (d, J = 5.8 Hz, 2H). ^13^C-NMR (151 MHz, Chloroform*-d*) δ 160.09 (C), 157.21 (C), 155.11 (CH), 140.48 (C), 134.78 (C), 131.26 (CH), 130.20 (CH), 128.00 (CH), 127.98 (CH), 126.00 (CH), 125.61 (CH), 115.29 (C), 44.53 (CH_2_).

***N-*(4-chlorobenzyl)thieno[2,3*-d*]pyrimidin-4-amine, 4j**

The titled compound **4j** was prepared according to General Procedure A, using (4-chlorophenyl)methanamine (55 µL, 0.45 mmol, 1.1 eq) as corresponding amine. The crude mixture was purified by column chromatography on silica gel using as eluent a gradient of CyHex/AcOEt from 1:1 to 2:3 to afford the compound **4j** as a white solid (53 mg, 46%). m/z for C_13_H_10_ClN_3_S = 275.03 (calculated), 276.5 (found, [M+H]^+^). ^1^H-NMR (600 MHz, Chloroform*-d*) δ 8.65 (s, 1H), 7.72 (d, J = 5.4 Hz, 1H), 7.44 (d, J = 5.4 Hz, 1H), 7.32 (s, 4H), 5.37 – 5.29 (m, 1H), 4.85 (d, J = 5.8 Hz, 2H). ^13^C-NMR (151 MHz, Chloroform*-d*) δ 160.02 (C), 157.23 (C), 155.10 (CH), 136.88 (C), 133.66 (C), 131.24 (CH), 129.29 (CH, 2C), 129.07 (CH, 2C), 125.59 (CH), 115.27 (C), 44.47 (CH_2_).

**3-((thieno[2,3*-d*]pyrimidin-4-ylamino)methyl)benzonitrile, 4l**

The titled compound **4l** was prepared according to General Procedure A, using 3-(aminomethyl)benzonitrile (60 mg, 0.45 mmol, 1.1 eq) as corresponding amine. The crude mixture was purified by column chromatography on silica gel using as eluent CyHex/AcOEt; 1:1. The compound was further purified by column chromatography using as eluent a mixture of DCM/MeOH, 95:5, and then using a mixture of AcOEt/toluene 1:1 to afford the compound **4l** as white solid (50 mg, 46%). m/z for C_14_H_10_N_4_S = 266.06 (calculated), 266.9 (found, [M+H]^+^). 1H NMR (600 MHz, DMSO*-d6*) δ 8.47 (t, J = 6.0 Hz, 1H), 8.43 (s, 1H), 8.14 (d, J = 5.4 Hz, 1H), 7.79 (td, J = 1.7, 0.8 Hz, 1H), 7.71 (dt, J = 7.7, 1.4 Hz, 1H), 7.70 – 7.67 (m, 1H), 7.56 – 7.52 (m, 1H), 7.40 (d, J = 5.4 Hz, 1H), 4.77 (d, J = 5.9 Hz, 2H). ^13^C NMR (151 MHz, DMSO) δ 159.46 (C), 156.73 (C), 154.44 (CH), 141.38 (C), 133.29 (CH), 132.16 (CH), 130.74 (CH), 130.64 (CH), 129.60 (CH), 124.44 (CH), 118.84 (C), 114.77 (C), 111.20 (C), 42.73 (CH_2_).

***N-*(3-methylbenzyl)thieno[2,3*-d*]pyrimidin-4-amine, 4n**

The titled compound **4n** was prepared according to General Procedure A, using m-tolylmethanamine (46 mg, 0.38 mmol, 1.3 eq) as corresponding amine. The crude mixture was purified by column chromatography on silica gel using as eluent a gradient of CyHex/AcOEt from 9:1 to 7:3 to afford the compound **4n** as a white solid (49 mg, 64%). m/z for C_14_H_13_N_3_S = 255.08 (calculated), 256.2 (found, [M+H]^+^), 278.1 (found, [M+Na]^+^). ^1^H NMR (600 MHz, DMSO*-d6*) δ 8.48 (t, J = 6.0 Hz, 1H), 8.34 (s, 1H), 7.66 (d, J = 6.0 Hz, 1H), 7.58 (d, J = 5.9 Hz, 1H), 7.20 (t, J = 7.5 Hz, 1H), 7.16 (dq, J = 1.8, 0.9 Hz, 1H), 7.13 (dtt, J = 7.6, 1.4, 0.7 Hz, 1H), 7.07 – 7.04 (m, 1H), 4.71 (d, J = 6.0 Hz, 2H), 2.33 – 2.22 (m, 3H). ^13^C NMR (151 MHz, DMSO*-d6*) δ 165.59 (C), 156.85 (C), 153.76 (CH), 139.39 (C), 137.40 (C), 128.26 (CH), 127.84 (CH), 127.48 (CH), 124.37 (CH), 122.74 (CH), 119.36 (CH), 116.10 (C), 43.17 (CH_2_), 21.04 (CH_3_).

***N-*(4-methylbenzyl)thieno[2,3*-d*]pyrimidin-4-amine, 4o**

The titled compound **4o** was prepared according to General Procedure A, using p-tolylmethanamine (57 µL, 0.45 mmol, 1.1 eq) as corresponding amine. The crude mixture was purified by column chromatography on silica gel using as eluent a mixture of CyHex/AcOEt, 1:1 to afford the compound **4o** as white solid (71 mg, 67%). m/z for C_14_H_13_N_3_S = 255.08 (calculated), 256.0 (found, [M+H]^+^). ^1^H NMR (600 MHz, DMSO*-d6*) δ 8.42 (s, 1H), 8.38 (t, J = 6.0 Hz, 1H), 8.11 (d, J = 5.4 Hz, 1H), 7.38 (d, J = 5.4 Hz, 1H), 7.25 – 7.21 (m, 2H), 7.14 – 7.10 (m, 2H), 4.69 (d, J = 6.0 Hz, 2H), 2.26 (s, 3H). ^13^C NMR (151 MHz, DMSO*-d6*) δ 159.34 (C), 156.82 (C), 154.51 (CH), 136.58 (C), 135.78 (C), 133.03 (CH), 128.82 (CH, 2C), 127.22 (CH, 2C), 124.43 (CH), 114.66 (C), 43.03 (CH_2_), 20.67 (CH_3_).

***N-*(4-fluoro-3-methoxybenzyl)thieno[2,3*-d*]pyrimidin-4-amine, 4p**

The titled compound **4p** was prepared according to General Procedure A, using (4-fluoro-3-methoxyphenyl)methanamine (40 mg, 0.26 mmol, 1.1 eq) as corresponding amine. The crude mixture was purified by column chromatography on silica gel using as eluent a mixture of CyHex/AcOEt, 1:1. The compound was further purified by column chromatography using as eluent a mixture of AcOEt/toluene, 1:1 to afford the compound **4p** as white solid (20 mg, 29%). m/z for C_14_H_12_FN_3_OS = 289.07 (calculated), 290.2 (found, [M+H]^+^), 307.3 (found, [M+H+NH3]^+^). ^1^H NMR (600 MHz, DMSO*-d6*) δ 8.44 (s, 1H), 8.39 (t, J = 6.0 Hz, 1H), 8.11 (d, J = 5.3 Hz, 1H), 7.39 (d, J = 5.4 Hz, 1H), 7.19 (dd, J = 8.5, 2.0 Hz, 1H), 7.12 (dd, J = 11.6, 8.3 Hz, 1H), 6.88 (ddd, J = 8.4, 4.5, 2.1 Hz, 1H), 4.69 (d, J = 5.9 Hz, 2H), 3.80 (s, 3H). ^13^C-NMR (151 MHz, DMSO*-d6*) δ 159.40 (C), 156.76 (C), 154.47 (CH), 150.50 (C, d, J = 243.0 Hz), 146.83 (C, d, J = 10.6 Hz), 136.32 (C, d, J = 4.5 Hz), 133.13 (CH), 124.43 (CH), 119.30 (CH, d, J= 7.5 Hz), 115.49 (CH, d, J = 18.1 Hz), 114.69 (C), 113.06 (CH, d, J = 1.5 Hz), 55.88 (CH_2_), 42.98 (CH_3_).

***N-*(4-chloro-3-methoxybenzyl)thieno[2,3*-d*]pyrimidin-4-amine, 4q**

The titled compound **4q** was prepared according to General Procedure A, using (4-chloro-3-methoxyphenyl)methanamine (55 mg, 0.32 mmol, 1.1 eq) as corresponding amine. The crude mixture was purified by column chromatography on silica gel using as eluent a gradient of CyHex/AcOEt from 9:1 to 7:3 to afford the compound **4q** as a white solid (72 mg, 80%). m/z for C_14_H_12_ClN_3_OS = 305.04 (calculated), 306.1 (found, [M+H]^+^). ^1^H NMR (600 MHz, DMSO*-d6*) δ 8.52 (t, J = 6.0 Hz, 1H), 8.35 (s, 1H), 7.65 (d, J = 6.0 Hz, 1H), 7.59 (d, J = 5.9 Hz, 1H), 7.35 (d, J = 8.1 Hz, 1H), 7.18 (d, J = 1.9 Hz, 1H), 6.91 (dd, J = 8.1, 1.9 Hz, 1H), 4.73 (d, J = 5.9 Hz, 2H), 3.83 (s, 3H). ^13^C NMR (151 MHz, DMSO*-d6*) δ 165.65 (C), 156.80 (C), 154.32 (C), 153.70 (CH), 140.19 (C), 129.63 (CH), 122.91 (CH), 119.98 (CH), 119.35 (C), 119.30 (CH), 116.15 (C), 111.89 (CH), 55.96 (CH_3_), 42.99 (CH_2_).

***N-*(1-(2-methoxyphenyl)ethyl)thieno[2,3*-d*]pyrimidin-4-amine, 5a**

The titled compound **5a** was prepared according to General Procedure A, using 1-(2-methoxyphenyl)ethan-1-amine (49 mg, 0.45 mmol, 1.1 eq) as corresponding amine. The crude mixture was purified by column chromatography on silica gel using as eluent a gradient of CyHex/AcOEt from 8:2 to 6:4 to afford the compound **5a** as a white solid (54 mg, 64%). m/z for C_15_H_15_N_3_OS = 285.09 (calculated), 286.1 (found, [M+H]^+^), 308.1 (found, [M+Na]^+^). ^1^H NMR (600 MHz, DMSO*-d6*) δ 8.25 (s, 1H), 8.14 (d, J = 8.0 Hz, 1H), 7.84 (d, J = 6.0 Hz, 1H), 7.58 (d, J = 5.9 Hz, 1H), 7.31 (dd, J = 7.6, 1.7 Hz, 1H), 7.19 (ddd, J = 8.2, 7.3, 1.7 Hz, 1H), 6.98 (dd, J = 8.3, 1.1 Hz, 1H), 6.87 (td, J = 7.4, 1.1 Hz, 1H), 5.75 (p, J = 7.1 Hz, 1H), 3.85 (s, 3H), 1.47 (d, J = 6.9 Hz, 3H). ^13^C NMR (151 MHz, DMSO*-d6*) δ 166.11 (C), 156.64 (C), 156.50 (C), 154.18 (CH), 133.16 (C), 128.20 (CH), 125.74 (CH), 122.96 (CH), 120.74 (CH), 120.16 (CH), 116.58 (C), 111.33 (CH), 55.90 (CH_3_), 44.61 (CH), 21.86 (CH_3_).

***N-*(1-(3-methoxyphenyl)ethyl)thieno[2,3*-d*]pyrimidin-4-amine, 5b**

The titled compound **5b** was prepared according to General Procedure A, using 1-(3-methoxyphenyl)ethan-1-amine (49 mg, 0.32 mmol, 1.1 eq) as corresponding amine. The crude mixture was purified by column chromatography on silica gel using as eluent a gradient of CyHex/AcOEt from 9:1 to 7:3 to afford the compound **5b** as a yellow gum (39 mg, 46%). m/z for C_15_H_15_N_3_OS = 285.09 (calculated), 286.1 (found, [M+H]^+^), 308.1 (found, [M+Na]^+^) and 593.4 (found, [2M+Na]^+^). ^1^H NMR (600 MHz, DMSO*-d6*) δ 8.28 (s, 1H), 8.20 (d, J = 8.0 Hz, 1H), 7.78 (d, J = 6.0 Hz, 1H), 7.58 (d, J = 5.9 Hz, 1H), 7.24 – 7.20 (m, 1H), 6.99 – 6.95 (m, 2H), 6.80 – 6.77 (m, 1H), 5.49 (p, J = 7.2 Hz, 1H), 3.72 (s, 3H), 1.54 (d, J = 7.1 Hz, 3H). ^13^C NMR (151 MHz, DMSO*-d6*) δ 165.68 (C), 159.27 (C), 156.09 (C), 153.64 (CH), 146.46 (C), 129.34 (CH), 122.61 (CH), 119.58 (CH), 118.26 (CH), 116.05 (C), 112.09 (CH), 111.67 (CH), 54.96 (CH3), 48.88 (CH), 22.49 (CH_3_).

***N-*(1-(4-fluorophenyl)ethyl)thieno[2,3*-d*]pyrimidin-4-amine, 5c**

The titled compound **5c** was prepared according to General Procedure A, using 1-(4-fluorophenyl)ethan-1-amine (59 µL, 0.45 mmol, 1.1 eq) as corresponding amine. The crude mixture was purified by column chromatography on silica gel using as eluent a mixture of CyHex/AcOEt, 1:1 to afford the compound **5c** as white solid (57 mg, 50%). m/z for C_14_H_12_FN_3_S = 273.07 (calculated), 274.1 (found, [M+H]^+^).^1^H-NMR (600 MHz, DMSO*-d6*) δ 8.37 (s, 1H), 8.23 (d, J = 7.9 Hz, 1H), 8.11 (d, J = 5.4 Hz, 1H), 7.47 – 7.42 (m, 2H), 7.36 (d, J = 5.4 Hz, 1H), 7.15 – 7.09 (m, 2H), 5.52 (p, J = 7.2 Hz, 1H), 1.53 (d, J = 7.1 Hz, 3H). ^13^C NMR (151 MHz, DMSO*-d6*) δ 161.54 (C, d, J = 241.5 Hz), 159.90 (C), 156.53 (C), 154.82 (CH), 141.54 (C, d, J = 3.0 Hz), 133.64 (CH), 128.46 (CH, d, J = 7.5 Hz, 2C), 124.82 (CH), 115.38 (CH, d, J = 21.1 Hz, 2C), 115.09 (C), 48.97 (CH), 22.94 (CH3).

***N-*(1-(4-chlorophenyl)ethyl)thieno[2,3*-d*]pyrimidin-4-amine, 5d**

The titled compound **5d** was prepared according to General Procedure A, using 1-(4-chlorophenyl)ethan-1-amine as (45 µL, 0.32 mmol, 1.1 eq) corresponding amine. The crude mixture was purified by column chromatography on silica gel using as eluent a mixture of CyHex/AcOEt, 7:3 to afford the compound **5d** as white solid (35 mg, 41%). m/z for C_14_H_12_ClN_3_S = 289.04-291.04 (calculated), 290.0-292.3 (found, [M+H]^+^). ^1^H NMR (600 MHz, DMSO*-d_6_*) δ 8.27 (s, 1H), 8.25 (d, J = 7.8 Hz, 1H), 7.76 (d, J = 6.0 Hz, 1H), 7.59 (d, J = 6.0 Hz, 1H), 7.44 – 7.41 (m, 2H), 7.38 – 7.35 (m, 2H), 5.48 (p, J = 7.2 Hz, 1H), 1.53 (d, J = 7.1 Hz, 3H). ^13^C NMR (151 MHz, DMSO*-d6*) δ 165.72 (C), 156.06 (C), 153.62 (CH), 143.89 (C), 131.15 (C), 128.27 (CH, 2C), 127.98 (CH, 2C), 122.79 (CH), 119.56 (CH), 116.11 (C), 48.56 (CH_2_), 22.40 (CH_2_).

***N-*(1-(p-tolyl)ethyl)thieno[2,3*-d*]pyrimidin-4-amine, 5e**

The titled compound **5e** was prepared according to General Procedure A, using 1-(p-tolyl)ethan-1-amine (39 µL, 0.32 mmol, 1.1 eq) as corresponding amine. The crude mixture was purified by column chromatography on silica gel using as eluent a mixture of CyHex/AcOEt, 6:4 to afford the compound **5e** as white solid (52 mg, 63%). m/z for C_15_H_15_N_3_S = 269.1 (calculated), 270.0 (found, [M+H]^+^), 292.2 (found, [M+Na]^+^). ^1^H NMR (600 MHz, DMSO*-d6*) δ 8.27 (s, 1H), 8.19 (d, J = 8.0 Hz, 1H), 7.76 (d, J = 6.0 Hz, 1H), 7.56 (d, J = 6.0 Hz, 1H), 7.30 – 7.26 (m, 2H), 7.11 (d, J = 7.9 Hz, 2H), 5.47 (p, J = 7.2 Hz, 1H), 2.25 (s, 3H), 1.52 (d, J = 7.0 Hz, 3H). ^13^C NMR (151 MHz, DMSO*-d6*) δ 166.08 (C), 156.56 (C), 154.11 (CH), 142.18 (C), 136.13 (C), 126.42 (CH, 2C), 123.01 (CH, 2C), 123.01 (CH), 120.07 (CH), 116.52 (C), 49.12 (CH), 22.92 (CH_3_), 21.08 (CH_3_).

***N-*phenylthieno[2,3*-d*]pyrimidin-4-amine, 5f**

The titled compound **5f** was prepared according to General Procedure A, using aniline (41 µL, 0.45 mmol, 1.1 eq) as corresponding amine. The crude mixture was purified by column chromatography on silica gel using as eluent a mixture of CyHex/AcOEt, 1:1 to afford the compound **5f** as brownish solid (76 mg, 81%). m/z for C_12_H_9_N_3_S = 227.05 (calculated), 228.1 (found, [M+H]^+^). ^1^H NMR (600 MHz, DMSO*-d*6) δ 9.69 (s, 1H), 8.57 (s, 1H), 8.22 (d, J = 5.4 Hz, 1H), 7.80 – 7.76 (m, 2H), 7.46 (d, J = 5.4 Hz, 1H), 7.39 – 7.34 (m, 2H), 7.12 (tt, J = 7.4, 1.2 Hz, 1H). ^13^C NMR (151 MHz, DMSO) δ 160.42 (C), 155.22 (C), 154.01 (CH), 139.09 (C), 134.29 (CH), 128.50 (CH, 2C), 124.38 (CH), 123.56 (CH), 122.17 (CH, 2C), 115.44 (C).

***N-*phenethylthieno[2,3*-d*]pyrimidin-4-amine, 5g**

The titled compound **5g** was prepared according to General Procedure A, using 2-phenylethan-1-amine (57 µL, 0.45 mmol, 1.1 eq) as corresponding amine. The crude mixture was purified by column chromatography on silica gel using as eluent a gradient of CyHex/AcOEt from 1:1 to 2:3 to afford the compound **5g** as white solid (70 mg, 66%) . m/z for C_14_H_13_N_3_S = 255.08 (calculated), 256.1 (found, [M+H]^+^). 1H NMR (600 MHz, Chloroform*-d*) δ 8.65 (s, 1H), 7.69 (dd, J = 5.4, 1.0 Hz, 1H), 7.42 (dd, J = 5.4, 1.0 Hz, 1H), 7.33 (t, J = 7.4 Hz, 2H), 7.28 – 7.23 (m, 3H), 4.92 (t, J = 5.7 Hz, 1H), 3.96 – 3.91 (m, 2H), 3.02 (t, J = 7.0 Hz, 2H). ^13^C NMR (151 MHz, Chloroform*-d*) δ 160.01 (C), 157.38 (C), 155.25 (CH), 138.83 (C), 130.89 (CH), 129.03 (CH, 2C), 128.89 (CH, 2C), 126.82 (CH), 125.66 (CH), 115.34 (C), 42.52 (CH_2_), 35.86 (CH_2_).

***N-*(3-phenylpropyl)thieno[2,3*-d*]pyrimidin-4-amine, 5h**

The titled compound **5h** was prepared according to General Procedure A, using 3-phenylpropan-1-amine (64 µL, 0.45 mmol, 1.1 eq) as corresponding amine. The crude mixture was purified by column chromatography on silica gel using as eluent a gradient of CyHex/AcOEt from 1:1 to 4:6 to afford the compound **5h** as a brownish solid (53 mg, 48%). m/z for C_15_H_15_N_3_S = 269.10 (calculated), 270.3 (found, [M+H]^+^). ^1^H-NMR (600 MHz, Chloroform*-d*) δ 8.62 (s, 1H), 7.69 (d, J = 5.3 Hz, 1H), 7.42 (d, J = 5.4 Hz, 1H), 7.30 (dd, J = 8.3, 6.9 Hz, 2H), 7.24 – 7.19 (m, 3H), 3.72 (td, J = 7.1, 5.8 Hz, 2H), 2.77 (t, J = 7.6 Hz, 2H), 2.11 – 2.03 (m, 2H). ^13^C-NMR (151 MHz, Chloroform*-d*) δ 159.61 (C), 157.49 (C), 154.99 (CH), 141.40 (C), 131.00 (CH), 128.73 (CH, 2C), 128.53 (CH, 2C), 126.31 (CH), 125.50 (CH), 115.19 (C), 41.19 (CH_2_), 33.49 (CH_2_), 31.25 (CH_2_).

***N-*ethyl-*N-*(3-methoxybenzyl)thieno[2,3-d]pyrimidin-4-amine, 5i**

To a solution of the (3-methoxyphenyl)methanamine (0.18 mL, 1.5 mmol, 1 eq) and DIPEA (0.93 mL, 2.9 mmol, 3 eq) in DCM at room temperature was added acetic anhydride (0.46 mL, 2.9 mmol, 2 eq). The reaction mixture was stirred for 2 h then diluted with DCM (20 mL) and washed successively with an aqueous solution of NaOH 1M (2x20 mL), HCl 1M (2x20 mL), NaHCO3 (20 mL) then dried over anhydrous MgSO_4_, filtered and concentrated under reduced pressure to give *N-*(3-methoxybenzyl)acetamide as a colourless oil.

The crude acetamide was diluted in dry THF (5 mL) and LiAlH_4_ (4.4 mL, 4.4 mmol, 1 M in THF) was added dropwise at 0 °C. The reaction mixture was then stirred at rt for 2 days. Water (0.16 mL) was added to quench the hydride followed by dropwise addition of an aqueous solution of NaOH (0.16 mL, 15%/w) and water (0.5 mL) at 0 °C. After 15 min, the grey biphasic solution was allowed to warm up to rt, anhydrous MgSO_4_ was added and it was stirred for 15 min before being filtered and concentrated under reduced pressure to give *N-*(3-methoxybenzyl)ethanamine as a yellow oil.

The titled compound **5i** was prepared according to General Procedure A, step 2, starting from *N-*(3-methoxybenzyl)ethanamine prepared as corresponding amine. The crude mixture was purified by column chromatography on silica gel using as eluent a gradient of CyHex/AcOEt from 80:20 to 20:80 to afford the compound **5i** as slightly yellow oil (77 mg, 44%). m/z for C_16_H_17_N_3_OS = 299.11 (calculated), 300.0 (found, [M+H]^+^), 322.0 (found, [M+Na]^+^). ^1^H NMR (600 MHz, Methanol-*d4*) δ 8.36 (s, 1H), 7.34 (d, J = 6.2 Hz, 1H), 7.30 (d, J = 6.2 Hz, 1H), 7.27 – 7.23 (m, 1H), 6.88 – 6.81 (m, 3H), 5.04 (s, 2H), 3.84 (q, J = 7.1 Hz, 2H), 3.75 (s, 3H), 1.33 (t, J = 7.1 Hz, 3H). ^13^C NMR (151 MHz, Methanol-*d4*) δ 169.46 (C), 161.68 (C), 159.36 (C), 153.67 (CH), 140.50 (C), 130.95 (CH), 122.95 (CH), 122.27 (CH), 119.97 (CH), 116.37 (C), 113.66 (CH), 113.55 (CH), 55.62 (CH_3_), 53.30 (CH_2_), 45.79 (CH_2_), 13.06 (CH_3_).

***N-*methyl-*N-*phenethylthieno[2,3*-d*]pyrimidin-4-amine, 5j**

The titled compound **5j** was prepared according to General Procedure A, using *N-*methyl-2-phenylethan-1-amine (65 µL, 0.45 mmol, 1.1 eq) as corresponding amine. The crude mixture was purified by column chromatography on silica gel using as eluent a mixture of CyHex/AcOEt 1:1 to afford the compound **5j** as brownish oil (81 mg, 73%). m/z for C_15_H_15_N_3_S = 269.10 (calculated), 270.0 (found, [M+H]^+^). ^1^H NMR (600 MHz, Chloroform*-d*) δ 8.56 (s, 1H), 7.75 (dd, J = 5.6, 0.7 Hz, 1H), 7.47 (d, J = 5.5 Hz, 1H), 7.31 (t, J = 7.5 Hz, 2H), 7.28 – 7.25 (m, 2H), 7.24 (t, J = 7.3 Hz, 1H), 4.06 – 3.99 (m, 2H), 3.40 – 3.34 (m, 3H), 3.02 (dd, J = 8.8, 6.7 Hz, 2H). ^13^C NMR (151 MHz, Chloroform*-d*) δ 159.64 (C), 158.28 (C), 153.82 (CH), 138.65 (C), 132.16 (CH), 129.04 (CH, 2C), 128.81 (CH, 2C), 126.77 (CH), 124.72 (CH), 114.33 (C), 53.53 (CH_2_), 38.23 (CH_3_), 34.57 (CH_2_).

***N-*(2-chlorophenethyl)thieno[2,3*-d*]pyrimidin-4-amine, 5k**

The titled compound **5k** was prepared according to General Procedure A, using 2-(2-chlorophenyl)ethan-1-amine (64 µL, 0.45 mmol, 1.1 eq) as corresponding amine. The crude mixture was purified by column chromatography on silica gel using as eluent a mixture of DCM/MeOH, 95:5. The product was further purified with a second column chromatography, using as eluent a mixture of CyHex/AcOEt 1:1 to afford the pure compound **5k** as white solid (84 mg, 70%). m/z for C_14_H_12_ClN_3_S = 289.04 (calculated), 289.9 (found, [M+H]^+^). ^1^H-NMR (600 MHz, DMSO*-d_6_*) δ 8.45 (s, 1H), 8.08 (d, J = 5.3 Hz, 1H), 7.99 (t, J = 5.6 Hz, 1H), 7.43 (dd, J = 7.2, 2.1 Hz, 1H), 7.37 (d, J = 5.4 Hz, 1H), 7.34 (dd, J = 7.0. 2.3 Hz, 1H), 7.25 (tt, J = 7.4, 5.4 Hz, 2H), 3.77 – 3.71 (m, 2H), 3.07 (t, J = 7.3 Hz, 2H). ^13^C NMR (151 MHz, Chloroform*-d*) δ 159.12 (C), 156.85 (C), 154.48 (CH), 136.91 (C), 133.20 (C), 132.90 (CH), 131.10 (CH), 129.22 (CH), 128.17 (CH), 127.25 (CH), 124.37 (CH), 114.72 (C), 39.97 (CH_2_), 32.56 (CH_2_).

***N-*(3-chlorophenethyl)thieno[2,3*-d*]pyrimidin-4-amine, 5l**

The titled compound **5l** was prepared according to General Procedure A, using 2-(3-chlorophenyl)ethan-1-amine (63 µL, 0.45 mmol, 1.1 eq) as corresponding amine. The crude mixture was purified by column chromatography on silica gel using as eluent a mixture of DCM/MeOH, 95:5. The product was further purified with a second column chromatography, using as eluent a mixture of CyHex/AcOEt to afford the pure compound **5l** as white solid (83 mg, 69%). m/z for C_14_H_12_ClN_3_S = 289.04 (calculated), 289.9 (found, [M+H]^+^). ^1^H NMR (600 MHz, DMSO*-d6*) δ 8.45 (s, 1H), 8.08 (d, J = 5.3 Hz, 1H), 7.93 (t, J = 5.5 Hz, 1H), 7.37 (d, J = 5.3 Hz, 1H), 7.33 (t, J = 1.9 Hz, 1H), 7.30 (d, J = 7.7 Hz, 1H), 7.25 (ddd, J = 8.0, 2.2, 1.2 Hz, 1H), 7.21 (dt, J = 7.6, 1.4 Hz, 1H), 3.75 – 3.70 (m, 2H), 2.95 (t, J = 7.3 Hz, 2H). ^13^C NMR (151 MHz, DMSO*-d6*) δ 159.22 (C), 156.80 (C), 154.52 (CH), 142.16 (C), 132.89 (C), 132.87 (CH), 130.11 (CH), 128.58 (CH), 127.50 (CH), 126.08 (CH), 124.43 (CH), 114.67 (C), 41.38 (CH_2_), 34.30 (CH_2_).

***N-*(4-chlorophenethyl)thieno[2,3*-d*]pyrimidin-4-amine, 5m**

The titled compound **5m** was prepared according to General Procedure A, using 2-(4-chlorophenyl)ethan-1-amine (63 µL, 0.45 mmol, 1.1 eq) as corresponding amine. The crude mixture was purified by column chromatography on silica gel using as eluent a mixture of CyHex/AcOEt, 1:1 to afford the compound **5m** as white solid (75 mg, 62%). m/z for C_14_H_12_ClN_3_S = 289.04 (calculated), 289.9 (found, [M+H]^+^). ^1^H NMR (600 MHz, Chloroform*-d*) δ 8.63 (s, 1H), 7.72 (d, J = 5.4 Hz, 1H), 7.45 (d, J = 5.4 Hz, 1H), 7.30 – 7.27 (m, 2H), 7.19 – 7.15 (m, 2H), 5.45 (s, 1H), 3.92 (q, J = 6.7 Hz, 2H), 3.00 (t, J = 7.0 Hz, 2H). ^13^C NMR (151 MHz, Chloroform*-d*) δ 158.48 (C), 157.40 (C), 154.32 (CH), 137.21 (C), 132.66 (C), 131.68 (CH), 130.35 (CH, 2C), 128.97 (CH, 2C), 124.95 (CH), 115.42 (C), 42.54 (CH_2_), 35.15 (CH_2_).

***N-*(3-methoxyphenethyl)thieno[2,3*-d*]pyrimidin-4-amine, 5n**

The titled compound **5n** was prepared according to General Procedure A, using 2-(3-methoxyphenyl)ethan-1-amine (66 µL, 0.45 mmol, 1.1 eq) as corresponding amine. The crude mixture was purified by column chromatography on silica gel using as eluent a mixture of CyHex/AcOEt, 6:4 to afford the compound **5n** as white solid (86 mg, 73%). m/z for C_15_H_15_N_3_OS = 285.09 (calculated), 286.0 (found, [M+H]^+^). ^1^H NMR (600 MHz, DMSO*-d_6_*) δ 8.36 (s, 1H), 8.07 (t, J = 5.6 Hz, 1H), 7.59 (d, J = 6.0 Hz, 1H), 7.55 (d, J = 5.9 Hz, 1H), 7.20 (ddd, J = 8.0, 7.1, 0.7 Hz, 1H), 6.84 – 6.81 (m, 2H), 6.77 (ddd, J = 8.3, 2.5, 1.1 Hz, 1H), 3.74 – 3.68 (m, 5H), 2.91 (t, J = 7.4 Hz, 2H). ^13^C NMR (151 MHz, DMSO*-d6*) δ 165.42 (C), 159.28 (C), 156.84 (C), 153.82 (CH), 141.07 (C), 129.36 (CH), 122.56 (CH), 120.94 (CH), 119.30 (CH), 116.14 (C), 114.27 (CH), 111.61 (CH), 54.86 (CH_3_), 41.58 (CH_2_), 34.80 (CH_2_).

## General procedure B: library exploration at position 4 of thieno[2,3*-d*]pyrimidine

**Step 1 – thieno[2,3*-d*]pyrimidin-4-amine, 6**

The suspension of 4-chlorothieno[2,3*-d*]pyrimidine (200 mg, 1.17 mmol, 1 eq) in 25% aqueous ammonia (12 mL) was heated at 90°C overnight in a sealed vial. The mixture was cooled to rt and diluted with water. The aqueous layer was extracted three times with ethyl acetate. The combined organic layers were washed with brine, dried over anhydrous MgSO_4_, filtered and evaporated to afford a white residue (135 mg, 75%), which was used as such for the next step. m/z for C_6_H_5_N_3_S = 151.02 (calculated), 152.0 (found, [M+H]^+^). ^1^H NMR (600 MHz, Methanol*-d_4_*) δ 8.26 (s, 1H), 7.50 – 7.44 (m, 2H).

**Step 2 – library exploration through alkylation of thieno[2,3*-d*]pyrimidin-4-amine**

To a solution of thieno[2,3*-d*]pyrimidin-4-amine (1 eq, 0.37 mmol) in dry THF (0.1 M) at 0°C, NaH 60% wt (1.5 eq) was added and the mixture was stirred at rt for 30 min. Then, the reaction mixture was cooled to 0°C and the corresponding benzyl bromide derivative (1 eq) was added. The reaction mixture was allowed to stir at rt overnight, was quenched with water and extracted with ethyl acetate three times. The combined organic layers were dried over anhydrous MgSO_4_, filtered and evaporated. The crude mixture was purified by column chromatography on silica gel.

***N-*(3-fluorobenzyl)thieno[2,3*-d*]pyrimidin-4-amine, 4f**

The titled compound **4f** was prepared according to General Procedure B, step 2, starting from 1-(bromomethyl)-4-fluorobenzene (41 µL, 0.33 mmol, 1 eq) as corresponding benzyl bromide. The crude mixture was purified by column chromatography on silica gel using as eluent a gradient of DCM/MeOH from 100:0 to 95:5 to afford the compound **4f** as white solid (22 mg, 25%). m/z for C_13_H_10_FN_3_S = 259.06 (calculated), 260.0 (found, [M+H]^+^). ^1^H NMR (600 MHz, Methanol*-d*4) δ 8.33 (s, 1H), 7.51 (d, J = 6.0 Hz, 1H), 7.48 (d, J = 6.0 Hz, 1H), 7.32 (td, J = 8.0, 5.9 Hz, 1H), 7.19 (ddd, J = 7.7, 1.6, 0.9 Hz, 1H), 7.10 (ddd, J = 10.1, 2.6, 1.6 Hz, 1H), 7.00 – 6.93 (m, 1H), 4.81 (s, 2H). ^13^C NMR (151 MHz, MeOD) δ 165.85 (C, d, J = 191.7 Hz), 163.60 (C), 158.72 (C), 154.65 (CH), 143.41 (C, d, J = 6.0 Hz), 131.23 (CH, d, J = 7.5 Hz), 124.20 (CH, d, J = 3.0 Hz), 124.11 (CH), 119.73 (CH), 118.04 (C), 115.13 (CH, d, J = 21.1 Hz), 114.76 (CH, d, J = 21.1), 44.61 (CH_2_, d, J = 1.5 Hz).

**2-((thieno[2,3*-d*]pyrimidin-4-ylamino)methyl)benzonitrile, 4k**

The titled compound **4k** was prepared according to General Procedure B, step 2, starting from 2-(bromomethyl)benzonitrile (61 mg, 0.31 mmol, 1 eq) as corresponding benzyl bromide. The crude mixture was purified by column chromatography on silica gel using as eluent a gradient of DCM/MeOH from 95:5 to 90:10 to afford the compound **4k** as yellow solid (36 mg, 43%). m/z for C_14_H_10_N_4_S = 266.06 (calculated), 267.1 (found, [M+H]^+^), 289.1 (found, [M+Na]^+^). ^1^H NMR (600 MHz, DMSO*-d6*) δ 8.66 (t, J = 5.8 Hz, 1H), 8.34 (s, 1H), 7.84 (dd, J = 7.7, 1.3 Hz, 1H), 7.68 – 7.64 (m, 2H), 7.62 (d, J = 5.9 Hz, 1H), 7.54 – 7.51 (m, 1H), 7.46 (td, J = 7.6, 1.2 Hz, 1H), 4.90 (d, J = 5.7 Hz, 2H). ^13^C NMR (151 MHz, DMSO*-d6*) δ 165.77 (C), 156.75 (C), 153.66 (CH), 143.00 (C), 133.46 (CH), 133.03 (CH), 128.05 (CH), 127.90 (CH), 123.24 (CH), 119.31 (CH), 117.50 (C), 116.28 (C), 110.56 (C), 41.96 (CH_2_).

**4-((thieno[2,3*-d*]pyrimidin-4-ylamino)methyl)benzonitrile, 4m**

The titled compound **4m** was prepared according to General Procedure B, step 2, starting from 4-(bromomethyl)benzonitrile (61 mg, 0.31 mmol, 1 eq) as corresponding benzyl bromide. The crude mixture was purified by column chromatography on silica gel using as eluent a gradient of CyHex/AcOEt from 70:30 to 40:60 to afford the compound **4m** as white solid (18 mg, 22%). m/z for C_14_H_10_N_4_S = 266.06 (calculated), 267.0 (found, [M+H]^+^). ^1^H NMR (600 MHz, DMSO*-d6*) δ 8.63 (t, J = 6.1 Hz, 1H), 8.32 (s, 1H), 7.79 (d, J = 8.1 Hz, 2H), 7.65 (d, J = 6.0 Hz, 1H), 7.62 (dd, J = 6.0, 0.9 Hz, 1H), 7.53 (d, J = 8.1 Hz, 2H), 4.82 (d, J = 6.0 Hz, 2H). ^13^C NMR (151 MHz, DMSO*-d6*) δ 165.70 (C), 156.78 (C), 153.67 (CH), 145.59 (C), 132.32 (CH, 2C), 127.96 (CH, 2C), 123.14 (CH), 119.19 (CH), 118.90 (C), 116.17 (C), 109.53 (C), 43.00 (CH_2_).

## Procedures for linker exploration with thieno[2,3*-d*]pyrimidine core

**3-methoxy-*N-*(thieno[2,3*-d*]pyrimidin-4-yl)benzamide, 7**

The mixture of thieno[2,3*-d*]pyrimidin-4-amine, 6 (50 mg, 0.33 mmol, 1 eq), 3-methoxybenzoyl chloride (85 mg, 0.50 mmol, 1.5 eq) in pyridine (4.1 mL) was stirred at room temperature overnight. MeOH was added to quench the excess of 3-methoxybenzoyl chloride at 0°C. The reaction mixture was poured into iced water, and the aqueous layer was extracted three times with ethyl acetate. The combined organic layers were washed with aqueous NaHSO_4_ (15%) three times, water, and aqueous saturated NaHCO_3_ twice, dried over anhydrous MgSO_4_, filtered and evaporated. The crude was purified on silica gel by column chromatography using as eluent DCM/MeOH 95:5 to afford the compound **7** as pale yellow solid (40 mg, 42%). m/z for C_14_H_11_N_3_O_2_S = 285.06 (calculated), 285.8 (found, [M+H]^+^). ^1^H-NMR (600 MHz, DMSO*-d_6_*) δ 11.57 (s, 1H), 8.95 (s, 1H), 8.41 (d, J = 5.6 Hz, 1H), 7.70 – 7.62 (m, 2H), 7.57 (d, J = 5.5 Hz, 1H), 7.48 (t, J = 7.9 Hz, 1H), 7.23 (ddd, J = 8.3, 2.6, 1.0 Hz, 1H), 3.86 (s, 3H). ^13^C NMR (151 MHz, DMSO*-d6*) δ 166.43 (C), 163.25 (C), 159.69 (C, 2C), 154.19 (C), 153.81 (CH), 139.03 (CH), 134.33 (C), 130.18 (CH), 123.76 (CH), 121.42 (CH), 119.68 (CH), 113.68 (CH), 55.89 (CH_3_).

## Procedures for core replacement exploration

***N-*(3-methoxybenzyl)-7-methylthieno[3,2*-d*]pyrimidin-4-amine, 10**

To a solution of 3-methoxybenzylamine (0.13 mL, 1.0 mmol, 2 eq) and K_2_CO_3_ (418 mg, 3.0 mmol, 6 eq) in DMF (3 mL) was added 4-chloro-7-methylthieno[3,2*-d*]pyrimidine (100 mg, 0.5 mmol, 1 eq). The reaction mixture was stirred at 80 °C overnight. The mixture was cooled down to rt, and diluted with AcOEt (20 mL) and water (20 mL). After separation, the organic layer was washed with brine (3x20 mL), dried over anhydrous MgSO_4_, filtered and concentrated under reduced pressure. The crude was purified on silica gel by column chromatography using as eluent a gradient of CyHex/AcOEt from 100:0 to 70:30) to afford the compound **10** as a slightly orange solid (117 mg, 40% yield). m/z for C_15_H_15_N_3_OS = 285.09 (calculated), 286.1 (found, [M+H]^+^). ^1^H NMR (600 MHz, MeOD*-d_4_*) *δ* 8.43 (s, 1H), 7.54 (d, J = 1.1 Hz, 1H), 7.19 (t, J = 8.1 Hz, 1H), 6.92 (dd, J = 4.3, 1.8 Hz, 2H), 6.77 (dd, J = 8.1, 2.2 Hz, 1H), 4.77 (s, 2H), 3.73 (s, 3H), 2.38 (d, J = 1.2 Hz, 3H).^13^C NMR (151 MHz, MeOD*-d_4_*) *δ* 161.29 (C), 158.96 (C), 158.58 (C), 155.19 (CH), 141.99 (C), 134.13 (C), 130.47 (CH), 128.60 (CH), 120.57 (CH), 117.08 (C), 114.02 (CH), 113.43 (CH), 55.56 (CH_3_), 45.05 (CH_2_), 12.98 (CH_3_).

**Step 1: 6-chloro-*N-*(3-methoxybenzyl)pyrimidin-4-amine, 11**

To a solution of 4,6*-d*ichloropyrimidine (150 mg, 1.01 mmol, 1 eq) in DMF (6.7 mL), (3-methoxyphenyl)methanamine (142 µL, 1.11 mmol, 1.1 eq.) and diisopropylethylamine (203 µL, 1.17 mmol, 1.15 eq) were added. The reaction mixture was stirred at 100°C for 4h then cooled to rt. Water was added and extracted three times with ethyl acetate. The combined organic layers were washed with brine twice, dried over anhydrous MgSO_4_, filtered and evaporated. The crude mixture was purified on silica gel by column chromatography using as eluent a mixture of CyHex/AcOEt, 1:1 to afford the compound **11** as a white solid (163 mg, 58%). m/z for C_12_H_13_ClN_3_O = 249.07-251.06 (calculated), 250.0-252.0 (found, [M+H]^+^). ^1^H NMR (600 MHz, DMSO*-d6*) δ 8.28 (s, 1H), 8.17 (s, 1H), 7.24 (t, J = 8.0 Hz, 1H), 6.87 (d, J = 6.0 Hz, 2H), 6.82 (ddd, J = 8.2, 2.6, 1.0 Hz, 1H), 6.59 (s, 1H), 4.53 (d, J = 5.8 Hz, 2H), 3.73 (s, 3H). ^13^C NMR (151 MHz, DMSO*-d6*) δ 163.13 (C), 159.39 (C), 158.62 (CH), 157.15 (C), 140.52 (C), 129.55 (CH), 119.50 (CH), 113.06 (CH), 112.36 (CH), 103.77 (CH), 55.02 (CH_3_), 43.55 (CH_2_).

**Step 2: *N-*(3-methoxybenzyl)pyrimidin-4-amine, 12**

The solution of 6-chloro-*N-*(3-methoxybenzyl)pyrimidin-4-amine **11** (45 mg, 0.18 mmol, 1 eq) in EtOH (2 mL) was added Pd/C 10%wt (13.5 mg, 30% m/m) and ammonium formate (67 mg, 1.06 mmol, 5.9 eq). The reaction mixture was degassed for 5 min, and then refluxed for 6 h. The reaction was left to cool to rt, filtered over a celite pad and the filtrate was evaporated to dryness. The crude mixture was purified on silica gel by column chromatography using as eluent a gradient of DCM/MeOH from 100:0 to 95:5 to afford compound **12** as a yellow oil (23 mg, 59%). m/z for C_12_H_13_N_3_O = 215.11 (calculated), 261.1 (found, [M+H]+). 1H NMR (600 MHz, Methanol*-d*4) δ 8.39 (s, 1H), 7.99 (d, J = 6.3 Hz, 1H), 7.23 (dd, J = 8.5, 7.2 Hz, 1H), 6.90 (dd, J = 7.8, 1.6 Hz, 2H), 6.83 – 6.79 (m, 1H), 6.52 (s, 1H), 4.55 (s, 2H), 3.77 (s, 3H). ^13^C NMR (151 MHz, Methanol*-d*4) δ 161.42 (C, 2C), 158.89 (CH, 2C), 143.26 (C), 130.62 (CH), 120.63 (CH), 114.05 (CH), 113.61 (CH, 2C), 55.60 (CH_3_), 30.76 (CH_2_).

***N-*(3-methoxybenzyl)isoquinolin-1-amine, 13**

To a solution of the isoquinolin-1-amine (50 mg, 0.3 mmol, 1 eq) in DMF (1 mL) was added at room temperature sodium *tert*-butoxide (34 mg, 0.4 mmol, 1.1 eq). The reaction mixture was stirred for 30 min and then 1-(chloromethyl)-3-methoxybenzene was added (0.06 mL, 0.5 mmol, 1.5 eq). The reaction mixture was stirred for 2 days at room temperature then diluted with water (20 mL) and extracted with EtOAc (20 mL). The organic layer was washed with brine (3x20 mL), dried over anhydrous MgSO_4_, filtered and evaporated to dryness. The crude mixture was purified twice on silica gel by column chromatography using as eluent a mixture of CyHex/AcOEt, 30:70, then a gradient of CyHex/AcOEt from 90:10 to 70:30 to afford compound **13** as a white solid (5.6 mg, 7%). m/z for C_17_H_16_N_2_O = 264.13 (calculated), 264.1 (found, [M+H]+). ^1^H NMR (600 MHz, DMSO-*d6*) δ 8.30 (dd, J = 8.5, 1.1 Hz, 1H), 7.95 (t, J = 6.0 Hz, 1H), 7.82 (d, J = 5.8 Hz, 1H), 7.73 – 7.69 (m, 1H), 7.63 (ddd, J = 8.1, 6.9, 1.1 Hz, 1H), 7.50 (ddd, J = 8.3, 6.9, 1.3 Hz, 1H), 7.20 (t, J = 8.1 Hz, 1H), 6.95 – 6.91 (m, 2H), 6.89 (dd, J = 5.8, 0.8 Hz, 1H), 6.77 (ddd, J = 8.2, 2.6, 1.0 Hz, 1H), 4.72 (d, J = 5.9 Hz, 2H), 3.70 (s, 3H). ^13^C NMR (151 MHz, DMSO-*d6*) δ 159.20 (C), 155.10 (C), 142.52 (C), 141.38 (CH), 136.67 (C), 129.71 (CH), 129.13 (CH), 126.50 (CH), 125.62 (CH), 122.96 (CH), 119.29 (CH), 117.77 (C), 112.91 (CH), 111.53 (CH), 109.67 (CH), 54.89 (CH_3_), 43.79 (CH_2_).

### Synthesis of *N-*(3-methoxybenzyl)quinolin-4-amine

 **Step 1:** ***tert-*butyl quinolin-4-ylcarbamate, 14**

A solution of quinolin-4-amine (500 mg, 3.47 mmol, 1 eq), DMAP (42.4 mg, 0.35 mmol, 0.1 eq) and di-*tert-*butyl dicarbonate (842 mg, 4.16 mmol, 1.2 eq.) in DCM (17 mL) was stirred at room temperature overnight. The reaction mixture was then evaporated to dryness. The crude mixture was purified on silica gel by column chromatography using as eluent a mixture of CyHex/AcOEt, 30:70 then a gradient of DCM/MeOH from 95:5 to 90:10 to afford compound, *tert-*butyl quinolin-4-ylcarbamate, **14** as a yellow solid (m= 691 mg, 71%). m/z for C_14_H_16_N_2_O_2_ = 244.12 (calculated), 189.0 (found, [M+2H-*t*ert-Bu]+), 145.0 (found, [M-CO_2_-*t*ert-Bu]+). ^1^H NMR (600 MHz, DMSO-*d6*) δ 9.78 (s, 1H), 8.74 (d, J = 5.1 Hz, 1H), 8.35 (ddd, J = 8.4, 1.4, 0.6 Hz, 1H), 7.97 – 7.94 (m, 1H), 7.90 (d, J = 5.1 Hz, 1H), 7.73 (ddd, J = 8.3, 6.8, 1.3 Hz, 1H), 7.55 (ddd, J = 8.3, 6.8, 1.3 Hz, 1H), 1.54 (s, 9H).

**Step 2:** ***tert-*butyl (3-methoxybenzyl)(quinolin-4-yl)carbamate, 15**

To a solution of *tert-*butyl quinolin-4-ylcarbamate **14** (297 mg, 1.22 mmol, 1 eq) in DCM (6 mL) was added at 0°C, NaH (58 mg, 60%, 1.2 eq) and the mixture was stirred at room temperature for 30 min. Then the reaction was cooled to 0°C and reagent 1-(bromomethyl)-3-methoxybenzene (179 µL, 1.05 eq) was added and the reaction was allowed to stir at room temperature for 24h. The reaction mixture was quenched with water (15mL) and extracted with AcOEt (3x25mL). The organic layers were dried over anhydrous MgSO_4_ and evaporated to dryness. The crude mixture was purified on silica gel by column chromatography using as eluent a gradient of DCM/MeOH, from 95:5 to 90:10, to afford compound, *tert-*butyl (3-methoxybenzyl)(quinolin-4-yl)carbamate, **15** as a yellow solid (m= 63.1 mg, 13%). m/z for C_22_H_24_N_2_O_3_ = 364.18 (calculated), 286.1 (found, [M+H]+), 264.2 [M+2H-*tert*-Bu]+). ^1^H NMR (600 MHz, DMSO-*d6*) δ 8.45 (d, J = 8.2 Hz, 1H), 8.28 – 8.19 (m, 1H), 7.69 (dd, J = 14.4, 7.2 Hz, 2H), 7.46 – 7.39 (m, 1H), 7.25 (td, J = 7.7, 0.9 Hz, 1H), 7.07 (s, 1H), 6.89 – 6.83 (m, 2H), 6.76 – 6.71 (m, 1H), 5.57 (s, 2H), 3.71 (s, 3H), 1.49 (s, 9H).

**Step 3:** ***N-*(3-methoxybenzyl)quinolin-4-amine, 16**

To a solution of *tert-*butyl (3-methoxybenzyl)(quinolin-4-yl)carbamate **15** (63 mg, 0.17 mmol, 1 eq) in DCM (0.3 mL) was added a 4M solution of hydrogen chloride in dioxane (1.9 mL, 45 eq.). The resulting mixture was stirred at room temperature for three days. A precipitate appeared, which was filtered and washed with Et_2_O to afford the HCl salt of *N-*(3-methoxybenzyl)quinolin-4-amine **16** as a beige solid (40 mg, 74%). m/z for C_17_H_16_N_2_O = 264.13 (calculated), 265.2 (found, [M+H]+). ^1^H NMR (600 MHz, DMSO-*d6*) δ 9.27 (s, 1H), 9.21 (s, 1H), 8.71 (d, J = 7.2 Hz, 1H), 8.52 (dd, J = 8.4, 1.4 Hz, 1H), 7.99 (dd, J = 8.9, 1.1 Hz, 1H), 7.95 (ddd, J = 8.7, 6.8, 1.4 Hz, 1H), 7.71 (ddd, J = 8.2, 6.8, 1.1 Hz, 1H), 7.29 – 7.23 (m, 1H), 6.92 – 6.86 (m, 3H), 6.73 (dt, J = 7.9, 1.2 Hz, 1H), 5.79 (s, 2H), 3.72 (s, 3H). ^13^C NMR (151 MHz, DMSO-*d6*) δ 159.58 (C), 158.32 (C), 146.81 (CH), 138.26 (C), 136.93 (C), 134.47 (CH), 130.18 (CH), 126.44 (CH), 124.65 (CH), 118.66 (CH), 118.45 (CH), 117.23 (C), 113.16 (CH), 112.80 (CH), 102.28 (CH), 56.40 (CH_2_), 55.12 (CH_3_).

## Procedures for library exploration of quinazoline core

### General Procedure C for nucleophilic aromatic substitution of 2,4*-d*ichloroquinazoline

To the solution of 2,4-dichloroquinazoline (1 eq) in dry ACN (0.5 M) under argon atmosphere, the corresponding amine (1 eq.) and diisopropylethylamine (1.5 eq) were added and the mixture was stirred at rt overnight. Water was added and the aqueous layer was extracted three times with ethyl acetate. The combined organic layers were washed with brine three times, dried over anhydrous MgSO_4_, filtered and evaporated. The crude was purified on silica gel by column chromatography to afford the desired product.

**2-chloro-*N-*(2-methoxybenzyl)quinazolin-4-amine, 8a**

The titled compound **8a** was prepared according to General Procedure C, using 2-methoxybenzylamine (207 mg, 1.0 mmol, 1 eq) as corresponding amine. The crude mixture was purified on silica gel by column chromatography using as eluent a gradient of CyHex/AcOEt, 85:15 to 40:60 to afford the compound **8a** as white solid (274 mg, 61%). m/z for C_16_H_14_ClN_3_O = 299.08-301.08 (calculated), 300.1-302.1 (found, [M+H]^+^), 322.2-324.2 (found, [M+Na]^+^). ^1^H NMR (600 MHz, Chloroform*-d*) δ 7.75 (ddd, J = 8.3, 1.4, 0.6 Hz, 1H), 7.70 (ddd, J = 8.3, 6.8, 1.3 Hz, 1H), 7.63 – 7.59 (m, 1H), 7.42 (ddt, J = 8.2, 4.5, 2.0 Hz, 2H), 7.31 (ddd, J = 8.3, 7.5, 1.8 Hz, 1H), 6.96 (td, J = 7.4, 1.1 Hz, 1H), 6.93 (dd, J = 8.2, 1.0 Hz, 1H), 6.49 (t, J = 5.4 Hz, 1H), 4.86 (d, J = 5.5 Hz, 2H), 3.92 (s, 3H). 13C NMR (151 MHz, Chloroform*-d*) δ 160.79 (C), 157.97 (C, 2C), 151.01 (C), 133.48 (CH), 130.75 (CH), 129.57 (CH), 127.96 (CH), 126.15 (CH), 125.37 (C), 121.05 (CH), 120.89 (CH), 113.53 (C), 110.72 (CH), 55.67 (CH_3_), 41.83 (CH_2_).

**2-chloro-*N-*(3-methoxybenzyl)quinazolin-4-amine, 8b**

The titled compound **8b** was prepared according to General Procedure C, using (3-methoxyphenyl)methanamine (129 µL, 1.00 mmol, 1 eq) as corresponding amine. The crude mixture was purified on silica gel by column chromatography using as eluent a gradient of CyHex/AcOEt from 7:3 to 6:4 and was purified again by column chromatography using as eluent a mixture of CyHex/AcOEt, 3:2 to afford the compound **8b** as white solid (179 mg, 58%). m/z for C_16_H_14_ClN_3_O = 299.08-301.08 (calculated), 299.8 (found, [M+H]^+^). ^1^H NMR (600 MHz, DMSO*-d6*) δ 9.27 (s, 1H), 8.35 – 8.28 (m, 1H), 7.81 (ddd, J = 8.3, 7.0, 1.3 Hz, 1H), 7.67 – 7.62 (m, 1H), 7.55 (ddd, J = 8.3, 7.0, 1.2 Hz, 1H), 7.25 (t, J = 7.9 Hz, 1H), 6.97 (dd, J = 2.6, 1.5 Hz, 1H), 6.94 (ddd, J = 7.5, 1.7, 0.9 Hz, 1H), 6.83 (ddd, J = 8.2, 2.7, 0.9 Hz, 1H), 4.76 – 4.70 (m, 2H), 3.73 (s, 3H). ^13^C NMR (151 MHz, DMSO*-d6*) δ 161.11 (C), 159.31 (C), 156.87 (C), 150.38 (C), 140.09 (C), 133.75 (CH), 129.50 (CH), 126.70 (CH), 126.24 (CH), 123.10 (CH), 119.58 (CH), 113.50 (C), 113.29 (CH), 112.39 (CH), 54.98 (CH_3_), 43.88 (CH_2_).

**2-chloro-*N-*(4-methoxybenzyl)quinazolin-4-amine, 8c**

The titled compound **8c** was prepared according to General Procedure C, using 4-methoxybenzylamine (207 mg, 1.0 mmol, 1 eq) as corresponding amine. The crude mixture was purified on silica gel by column chromatography using as eluent a gradient of CyHex/AcOEt, 85:15 to 40:60 to afford the compound **8c** as white solid (305 mg, 68%). m/z for C_16_H_14_ClN_3_O = 299.08-301.08 (calculated), 322.2-324.1 (found, [M+Na]^+^). ^1^H NMR (600 MHz, Chloroform*-d*) δ 7.79 (ddd, J = 8.4, 1.3, 0.6 Hz, 1H), 7.74 (ddd, J = 8.4, 6.9, 1.3 Hz, 1H), 7.63 (dt, J = 8.1, 1.1 Hz, 1H), 7.44 (ddd, J = 8.2, 6.9, 1.3 Hz, 1H), 7.37 – 7.32 (m, 2H), 6.94 – 6.90 (m, 2H), 6.00 (s, 1H), 4.79 (d, J = 5.1 Hz, 2H), 3.82 (s, 3H). 13C NMR (151 MHz, Chloroform*-d*) δ 160.79 (C), 159.75 (C), 157.97 (C), 151.11 (C), 133.80 (CH), 130.05 (CH, 2C), 129.50 (C), 128.17 (CH), 126.48 (CH), 120.93 (CH), 114.60 (CH, 2C), 113.37 (C), 55.61 (CH3), 45.59 (CH2).

**2-chloro-*N-*(4-fluorobenzyl)quinazolin-4-amine, 8d**

The titled compound **8d** was prepared according to General Procedure C, using 4-fluorobenzylamine (189 mg, 1.5 mmol, 1 eq) as corresponding amine. The crude mixture was purified on silica gel by column chromatography using as eluent a gradient of CyHex/AcOEt, 85:15 to 40:60 to afford the compound **8d** as white solid (315 mg, 73%). m/z for C_15_H_11_ClFN_3_ = 287.06-289.06 (calculated), 288.0-290.0 (found, [M+H]^+^). ^1^H NMR (600 MHz, Chloroform-*d*) δ 7.80 (ddd, J = 8.4, 1.3, 0.6 Hz, 1H), 7.75 (ddd, J = 8.4, 6.9, 1.3 Hz, 1H), 7.67 – 7.63 (m, 1H), 7.46 (ddd, J = 8.2, 6.9, 1.3 Hz, 1H), 7.42 – 7.37 (m, 2H), 7.07 (ddt, J = 8.6, 6.5, 2.6 Hz, 2H), 6.04 (s, 1H), 4.84 (d, J = 5.4 Hz, 2H). ^13^C NMR (151 MHz, Chloroform-*d*) δ 161.86 (C), 162.12 (C, d, J = 412.0 Hz), 157.81 (C), 151.13 (C), 133.82 (CH), 133.27 (C, d, J = 4.5 Hz), 130.25 (CH, d, J = 9.1 Hz, 2C), 128.20 (CH), 126.49 (CH), 120.75 (CH), 116.01 (CH, d, J = 21.1 Hz, 2C), 113.22 (C), 45.14 (CH_2_).

**2-chloro-*N-*(4-chlorobenzyl)quinazolin-4-amine, 8e**

The titled compound **8e** was prepared according to General Procedure C, using 4-chlorobenzylamine (213 mg, 1.5 mmol, 1 eq) as corresponding amine. The crude mixture was purified on silica gel by column chromatography using as eluent a gradient of CyHex/AcOEt, 85:15 to 40:60 to afford the compound **8e** as white solid (305 mg, 67%). m/z for C_15_H_11_Cl_2_N_3_ = 303.03-305.03 (calculated), 304.0-306.0 (found, [M+H]^+^). ^1^H NMR (600 MHz, Chloroform-*d*) δ 7.80 (ddd, J = 8.4, 1.3, 0.6 Hz, 1H), 7.76 (ddd, J = 8.4, 6.9, 1.3 Hz, 1H), 7.68 – 7.64 (m, 1H), 7.46 (ddd, J = 8.2, 6.9, 1.3 Hz, 1H), 7.37 – 7.33 (m, 4H), 6.06 (s, 1H), 4.85 (d, J = 5.5 Hz, 2H). ^13^C NMR (151 MHz, Chloroform-*d*) δ 160.80 (C), 157.78 (C), 151.15 (C), 135.99 (C), 134.09 (C), 133.85 (CH), 129.81 (CH, 2C), 129.24 (CH, 2C), 128.22 (CH), 126.53 (CH), 120.73 (CH), 113.21 (C), 45.11 (CH_2_).

**2-chloro-*N-*(4-methylbenzyl)quinazolin-4-amine, 8f**

The titled compound **8f** was prepared according to General Procedure C, using 4-methylbenzylamine (183 mg, 1.5 mmol, 1 eq) as corresponding amine. The crude mixture was purified on silica gel by column chromatography using as eluent a gradient of CyHex/AcOEt, 85:15 to 40:60 to afford the compound **8f** as white solid (288 mg, 67%). m/z for C_16_H_14_ClN_3_ = 283.09-285.08 (calculated), 284.1-286.1 (found, [M+H]^+^), 306.1-308.1 (found, [M+Na]^+^). ^1^H NMR (600 MHz, Chloroform-*d*) δ 7.77 (dd, J = 8.4, 1.3 Hz, 1H), 7.72 (ddd, J = 8.4, 6.9, 1.3 Hz, 1H), 7.66 (dd, J = 8.3, 1.3 Hz, 1H), 7.43 (ddd, J = 8.2, 6.9, 1.3 Hz, 1H), 7.31 – 7.28 (m, 2H), 7.18 (d, J = 7.8 Hz, 2H), 6.15 (t, J = 5.4 Hz, 1H), 4.81 (d, J = 5.2 Hz, 2H), 2.36 (s, 3H). 13C NMR (151 MHz, Chloroform-*d*) δ 160.76 (C), 157.82 (C), 150.91 (C), 138.03 (C), 134.31 (C), 133.69 (CH), 129.75 (CH, 2C), 128.51 (CH, 2C), 127.93 (CH), 126.38 (CH), 120.92 (CH), 113.28 (C), 45.72 (CH_2_), 21.29 (CH_3_).

**2-chloro-*N-*(4-chlorophenethyl)quinazolin-4-amine, 8g**

The titled compound **8g** was prepared according to General Procedure C, using 2-(4-chlorophenyl)ethan-1-amine (141 µL, 1.00 mmol, 1 eq) as corresponding amine. The crude mixture was purified on silica gel by column chromatography using as eluent a mixture of CyHex/AcOEt, 7:3 to afford the compound **8g** as white solid (217 mg, 67%). m/z for C_16_H_13_Cl_2_N_3_ = 317.05-319.05 (calculated), 317.9-319.9 (found, [M+H]^+^). ^1^H NMR (600 MHz, Chloroform*-d*) δ 7.77 (ddd, J = 8.4, 1.4, 0.6 Hz, 1H), 7.73 (ddd, J = 8.3, 6.8, 1.3 Hz, 1H), 7.54 – 7.50 (m, 1H), 7.43 (ddd, J = 8.2, 6.8, 1.4 Hz, 1H), 7.33 – 7.29 (m, 2H), 7.22 – 7.17 (m, 2H), 5.90 (t, J = 5.5 Hz, 1H), 3.93 (td, J = 6.9, 5.7 Hz, 2H), 3.01 (t, J = 6.9 Hz, 2H). ^13^C NMR (151 MHz, Chloroform*-d*) δ 160.93 (C), 157.85 (C), 150.98 (C), 137.10 (C), 133.71 (CH), 132.82 (C), 130.34 (CH, 2C), 129.10 (CH, 2C), 128.11 (CH), 126.45 (CH), 120.60 (CH), 113.32 (C), 42.57 (CH_2_), 34.57 (CH_2_).

**2-chloro-*N-*(1-(4-fluorophenyl)ethyl)quinazolin-4-amine, 8h**

The titled compound **8h** was prepared according to General Procedure C, using 1-(4-fluorophenyl)ethan-1-amine (210 mg, 1.5 mmol, 1 eq) as corresponding amine. The crude mixture was purified on silica gel by column chromatography using as eluent a gradient of CyHex/AcOEt, 85:15 to 40:60 to afford the compound **8h** as white solid (290 mg, 64%). m/z for C_16_H_13_ClFN_3_ = 301.08-303.08 (calculated), 302.0-304.0 (found, [M+H]^+^). ^1^H NMR (600 MHz, Chloroform-*d*) δ 7.78 – 7.67 (m, 3H), 7.47 – 7.39 (m, 3H), 7.07 – 7.01 (m, 2H), 6.06 (d, J = 7.5 Hz, 1H), 5.63 (p, J = 7.0 Hz, 1H), 1.69 (d, J = 6.9 Hz, 3H). ^13^C NMR (151 MHz, Chloroform-*d*) δ 161.59 (C, d, J = 476.9 Hz), 161.54 (C), 157.77 (C), 151.02 (C), 138.30 (C, d, J = 3.0 Hz), 133.70 (CH), 128.37 (CH, d, J = 7.5 Hz, 2C), 128.04 (CH), 126.37 (CH), 120.81 (CH), 115.78 (CH, d, J = 21.1 Hz, 2C), 113.21 (C), 49.89 (CH), 21.38(CH_3_).

**2-chloro-*N-*(1-(p-tolyl)ethyl)quinazolin-4-amine, 8i**

The titled compound **8i** was prepared according to General Procedure C, using 1-(p-tolyl)ethan-1-amine (75 mg, 0.55 mmol, 1.1 eq) as corresponding amine. The crude mixture was purified on silica gel by column chromatography using as eluent a mixture of CyHex/AcOEt, 75:25 to afford the compound **8i** as white solid (91 mg, 60%). m/z for C_17_H_16_ClN_3_ = 297.10-299.10 (calculated), 298.0-300.0 (found, [M+H]^+^). ^1^H NMR (600 MHz, DMSO-*d6*) δ 8.89 (d, J = 7.7 Hz, 1H), 8.50 – 8.42 (m, 1H), 7.80 (ddd, J = 8.4, 7.0, 1.4 Hz, 1H), 7.61 (dd, J = 8.3, 1.2 Hz, 1H), 7.55 (ddd, J = 8.2, 7.0, 1.3 Hz, 1H), 7.36 – 7.29 (m, 2H), 7.16 – 7.11 (m, 2H), 5.49 (p, J = 7.2 Hz, 1H), 2.26 (s, 3H), 1.58 (d, J = 7.0 Hz, 3H).

### General procedures D for dechlorination of 2-chloroquinazoline

**Method A:** The solution of the corresponding 2-chloroquinazoline derivative (1 eq) in EtOH (0.1M) was degassed. Then, Pd/C 10%wt (30% m/m) and ammonium formate (5.9 eq) were added. The mixture was further degassed for 5 min, and then refluxed for 6h. The reaction was left to cool to rt and filtered on celite. The filtrate was evaporated and purified by column chromatography on silica gel.

**Method B:** To the suspension of the corresponding 2-chloroquinazoline derivative (1 eq) in MeOH (0.04 M), zinc (15 eq), *N,N,N′,N′*-Tetramethyl ethylenediamine (10 eq) and acetic acid (20 eq) were added at 0°C. The reaction mixture was heated at 40-45°C overnight. Then, the reaction mixture was cooled to rt, diluted with MeOH, and 2-mercaptonicotinic acid (15 eq) was added. The resulting suspension was stirred at rt for 30 min before diluting with ethyl acetate and washed with aqueous 2M NaOH twice. The combined aqueous layers were extracted twice with ethyl acetate. Then, the organic phase was washed with aqueous saturated NaHCO_3_, dried over anhydrous MgSO_4_, filtered and concentrated. The crude was purified on silica gel to afford the desired product.

***N-*(2-methoxybenzyl)quinazolin-4-amine, 9a**

The titled compound **9a** was prepared according to General Procedure D (method A), starting from 2-chloro-*N-*(2-methoxybenzyl)quinazolin-4-amine (**8a**, 100 mg, 0.33 mmol, 1 eq). The crude mixture was purified on silica gel by column chromatography using as eluent a mixture of CyHex/AcOEt, 30:70 to afford the compound **9a** as white solid (32 mg, 35%). m/z for C_16_H_15_N_3_O = 265.12 (calculated), 266.1 (found, [M+H]^+^), 288.1 (found, [M+Na]^+^). ^1^H NMR (600 MHz, Chloroform-*d*) δ 8.69 (s, 1H), 7.83 (d, J = 8.3 Hz, 1H), 7.75 – 7.69 (m, 1H), 7.66 (d, J = 8.2 Hz, 1H), 7.44 (t, J = 7.9 Hz, 1H), 7.42 – 7.38 (m, 1H), 7.30 (td, J = 7.9, 1.7 Hz, 1H), 6.98 – 6.91 (m, 2H), 6.27 (d, J = 5.8 Hz, 1H), 4.88 (d, J = 5.5 Hz, 2H), 3.92 (s, 3H). ^13^C NMR (151 MHz, Chloroform-*d*) δ 159.43 (C), 157.96 (C), 155.65 (CH), 149.64 (C), 132.60 (CH), 130.27 (CH), 129.29 (CH), 128.75 (CH), 126.09 (C), 125.98 (CH), 120.94 (CH), 120.63 (CH), 115.22 (C), 110.69 (CH), 55.63 (CH_3_), 41.38 (CH_2_).

***N-*(3-methoxybenzyl)quinazolin-4-amine, 9b**

The titled compound **9b** was prepared according to General Procedure D (method A), starting from 2-chloro-*N-*(3-methoxybenzyl)quinazolin-4-amine (**8b**, 50 mg, 0.17 mmol, 1 eq). The crude mixture was purified on silica gel by column chromatography using as eluent a mixture of CyHex/AcOEt, 1:1 to afford the compound **9b** as white solid (33 mg, 74%). m/z for C_16_H_15_N_3_O = 265.12 (calculated), 266.0 (found, [M+H]^+^). ^1^H NMR (600 MHz, DMSO*-d6*) δ 8.81 (t, J = 6.0 Hz, 1H), 8.45 (s, 1H), 8.30 (ddd, J = 8.4, 1.3, 0.6 Hz, 1H), 7.78 (ddd, J = 8.3, 6.9, 1.4 Hz, 1H), 7.70 (dd, J = 8.3, 1.2 Hz, 1H), 7.53 (ddd, J = 8.2, 6.9, 1.3 Hz, 1H), 7.25 – 7.19 (m, 1H), 6.96 – 6.90 (m, 2H), 6.80 (ddd, J = 8.2, 2.5, 1.1 Hz, 1H), 4.76 (d, J = 5.9 Hz, 2H), 3.71 (s, 3H). ^13^C NMR (151 MHz, DMSO-*d6*) δ 159.37 (C), 159.29 (C), 155.06 (CH), 149.18 (C), 141.08 (C), 132.62 (CH), 129.38 (CH), 127.54 (CH), 125.73 (CH), 122.65 (CH), 119.33 (CH), 114.90 (C), 113.01 (CH), 111.99 (CH), 54.96 (CH_3_), 43.45 (CH_2_).

***N-*(4-methoxybenzyl)quinazolin-4-amine, 9c**

The titled compound **9c** was prepared according to General Procedure D (method A), starting from 2-chloro-*N-*(4-methoxybenzyl)quinazolin-4-amine (**8c**, 100 mg, 0.33 mmol, 1 eq). The crude mixture was purified on silica gel by column chromatography using as eluent a gradient of CyHex/AcOEt from 85:15 to40:60 to afford the compound **9c** as white solid (53 mg, 60%). m/z for C_16_H_15_N_3_O = 265.12 (calculated), 266.1 (found, [M+H]^+^), 288.1 (found, [M+Na]^+^). ^1^H NMR (600 MHz, Chloroform-*d*) δ 8.70 (s, 1H), 7.85 (dd, J = 8.4, 1.2 Hz, 1H), 7.73 (ddd, J = 8.4, 7.0, 1.3 Hz, 1H), 7.69 (dd, J = 8.3, 1.3 Hz, 1H), 7.43 (ddd, J = 8.2, 7.0, 1.2 Hz, 1H), 7.35 – 7.30 (m, 2H), 6.92 – 6.87 (m, 2H), 5.98 (d, J = 6.2 Hz, 1H), 4.79 (d, J = 5.2 Hz, 2H), 3.81 (s, 3H). ^13^C NMR (151 MHz, Chloroform-*d*) δ 159.41 (C), 159.32 (C), 155.57 (CH), 149.62 (C), 132.75 (CH), 130.17 (C), 129.60 (CH), 129.58 (CH), 128.76 (CH), 126.15 (CH), 120.64 (CH), 114.98 (C), 114.38 (CH), 55.47 (CH_3_), 45.03 (CH_2_).

***N-*(4-fluoro)quinazolin-4-amine, 9d**

The titled compound **9d** was prepared according to General Procedure D (method A), starting from 2-chloro-*N-*(4-fluorobenzyl)quinazolin-4-amine (**8d**, 100 mg, 0.35 mmol, 1 eq). The crude mixture was purified on silica gel by column chromatography using as eluent a mixture of CyHex/AcOEt, 30:70 to afford the compound **9d** as white solid (11 mg, 11%). m/z for C_15_H_12_FN_3_ = 253.10 (calculated), 254.1 (found, [M+H]^+^). ^1^H NMR (600 MHz, Chloroform-*d*) δ 8.71 (s, 1H), 7.87 (d, J = 8.4 Hz, 1H), 7.76 (ddd, J = 8.3, 6.9, 1.3 Hz, 1H), 7.71 – 7.67 (m, 1H), 7.47 (ddd, J = 8.2, 7.0, 1.2 Hz, 1H), 7.41 – 7.36 (m, 2H), 7.06 (t, J = 8.6 Hz, 2H), 5.92 (t, J = 5.6 Hz, 1H), 4.85 (d, J = 5.5 Hz, 2H). ^13^C NMR (151 MHz, Chloroform-*d*) δ 163.34 (C), 160.52 (C, d, J = 357.7 Hz), 155.51 (CH), 149.71 (C), 134.02 (C, d, J = 3.0 Hz), 132.88 (CH), 129.86 (CH, d, J = 7.5 Hz, 2C), 128.93 (CH), 126.30 (CH), 120.50 (CH), 115.88 (CH, d, J = 21.1 Hz, 2C), 114.81 (C), 44.73 (CH_2_).

***N-*(4-chloro)quinazolin-4-amine, 9e**

The titled compound **9e** was prepared according to General Procedure D (method B), starting from 2-chloro-*N-*(4-chlorobenzyl)quinazolin-4-amine (**8e**, 100 mg, 0.33 mmol, 1 eq). The crude mixture was purified on silica gel by column chromatography using as eluent a mixture of CyHex/AcOEt, 30:70 to afford the compound **9e** as white solid (5 mg, 6%). m/z for C_15_H_12_ClN_3_ = 269.07-271.07 (calculated), 270.1-272.1 (found, [M+H]^+^). ^1^H NMR (600 MHz, Chloroform-*d*) δ 8.70 (s, 1H), 7.88 (d, J = 8.4 Hz, 1H), 7.76 (ddt, J = 8.2, 7.0, 1.1 Hz, 1H), 7.72 (d, J = 8.2 Hz, 1H), 7.48 (ddt, J = 8.2, 6.9, 1.1 Hz, 1H), 7.37 – 7.31 (m, 4H), 6.03 (s, 1H), 4.86 (d, J = 5.5 Hz, 2H). ^13^C NMR (151 MHz, Chloroform-*d*) δ 159.39 (C), 155.40 (CH), 149.53 (C), 136.78 (C), 133.73 (C), 132.96 (CH), 129.45 (CH, 2C), 129.12 (CH, 2C), 128.80 (CH), 126.38 (CH), 120.55 (CH), 114.87 (C), 44.70 (CH_2_).

***N-*(4-methyl)quinazolin-4-amine, 9f**

The titled compound **9f** was prepared according to General Procedure D (method A), starting from 2-chloro-*N-*(4-methylbenzyl)quinazolin-4-amine (**8f**, 100 mg, 0.35 mmol, 1 eq). The crude mixture was purified on silica gel by column chromatography using as eluent a mixture of CyHex/AcOEt, 30:70 to afford the compound **9f** as white solid (29 mg, 32%). m/z for C_16_H_15_N_3_ = 249.13 (calculated), 250.1 (found, [M+H]^+^), 272.1 (found, [M+Na]^+^). ^1^H NMR (600 MHz, Chloroform-*d*) δ 8.71 (s, 1H), 7.86 (d, J = 8.3 Hz, 1H), 7.74 (ddd, J = 8.4, 7.0, 1.3 Hz, 1H), 7.68 (dd, J = 8.4, 1.3 Hz, 1H), 7.45 (ddd, J = 8.2, 7.0, 1.3 Hz, 1H), 7.30 (d, J = 7.8 Hz, 2H), 7.18 (d, J = 7.8 Hz, 2H), 5.99 – 5.83 (m, 1H), 4.82 (d, J = 5.2 Hz, 2H), 2.36 (s, 3H). ^13^C NMR (151 MHz, Chloroform-*d*) δ 159.23 (C), 155.46 (CH), 149.52 (C), 137.63 (C), 134.97 (C), 132.63 (CH), 129.56 (CH, 2C), 128.70 (CH), 128.10 (CH, 2C), 126.04 (CH), 120.45 (CH), 114.85 (C), 45.20 (CH_2_), 21.15 (CH_3_).

***N-*(4-chlorophenethyl)quinazolin-4-amine, 9g**

The titled compound **9g** was prepared according to General Procedure D (method B), starting from 2-chloro-*N-*(4-chlorophenethyl)quinazolin-4-amine (**8g**, 50 mg, 0.16 mmol, 1 eq). The crude mixture was purified on silica gel by column chromatography using as eluent a mixture of CyHex/AcOEt, 1:1 to afford the compound **9g** as white solid (20 mg, 44%). m/z for C_16_H_14_ClN_3_O = 283.09-285.08 (calculated), 284.0-286.0 (found, [M+H]^+^). ^1^H NMR (600 MHz, Methanol-*d4*) δ 8.46 (s, 1H), 8.04 (ddd, J = 8.3, 1.4, 0.6 Hz, 1H), 7.79 (ddd, J = 8.3, 6.9, 1.4 Hz, 1H), 7.71 (dt, J = 8.3, 1.0 Hz, 1H), 7.52 (ddd, J = 8.3, 6.9, 1.3 Hz, 1H), 7.30 – 7.23 (m, 4H), 3.87 – 3.80 (m, 2H), 3.02 (dd, J = 8.1, 6.7 Hz, 2H). ^13^C NMR (151 MHz, MeOD-*d4*) δ 161.41 (C), 156.03 (CH), 149.62 (C), 139.54 (C), 134.21 (CH), 133.18 (C), 131.58 (CH, 2C), 129.51 (CH, 2C), 127.72 (CH), 127.48 (CH), 123.33 (CH), 116.47 (C), 43.58 (CH_2_), 35.38 (CH_2_).

***N-*(1-(4-fluorophenyl)ethyl)quinazolin-4-amine, 9h**

The titled compound **9h** was prepared according to General Procedure D (method A), starting from 2-chloro-*N-*(1-(4-fluorophenyl)ethyl)quinazolin-4-amine (**8h**, 90 mg, 0.30 mmol, 1 eq). The crude mixture was purified on silica gel by column chromatography using as eluent a gradient of CyHex/AcOEt, from 30:70 to 70:30 to afford the compound **9h** as white solid (43 mg, 54%). m/z for C_16_H_14_FN_3_ = 267.12 (calculated), 268.2 (found, [M+H]^+^), 290.2 (found, [M+Na]^+^). ^1^H NMR (600 MHz, DMSO-*d6*) δ 8.46 – 8.43 (m, 2H), 8.40 (s, 1H), 7.77 (ddd, J = 8.3, 6.9, 1.3 Hz, 1H), 7.67 (dd, J = 8.2, 1.2 Hz, 1H), 7.54 (ddd, J = 8.2, 6.9, 1.3 Hz, 1H), 7.49 – 7.45 (m, 2H), 7.15 – 7.10 (m, 2H), 5.59 (p, J = 7.2 Hz, 1H), 1.58 (d, J = 7.1 Hz, 3H). ^13^C NMR (151 MHz, DMSO-*d6*) δ 160.98 (C, d, J = 240.2 Hz), 158.55 (C), 154.94 (CH), 149.19 (C), 140.87 (C, d, J = 3.0 Hz), 132.59 (CH), 128.03 (CH, d, J = 9.0 Hz, 2C), 127.47 (CH), 125.56 (CH), 122.94 (CH), 114.91 (CH, d, J = 21.0 Hz, 2C), 114.80 (C), 48.64 (CH), 22.29 (CH_3_).

***N-*(1-(p-tolyl)ethyl)quinazolin-4-amine, 9i**

The titled compound **9i** was prepared according to General Procedure D (method A), starting from 2-chloro-*N-*(1-(p-tolyl)ethyl)quinazolin-4-amine (**8i**, 91 mg, 0.31 mmol, 1 eq). The crude mixture was purified on silica gel by column chromatography using as eluent a gradient of CyHex/AcOEt, from 30:70 to 70:30 to afford the compound **9i** as white solid (35 mg, 43%). m/z for C_17_H_17_N_3_ = 263.14 (calculated), 264.1 (found, [M+H]^+^), 286.1 (found, [M+Na]^+^). ^1^H NMR (600 MHz, DMSO-*d6*) δ 8.47 – 8.43 (m, 1H), 8.42 – 8.38 (m, 2H), 7.76 (ddd, J = 8.3, 6.9, 1.3 Hz, 1H), 7.66 (dd, J = 8.4, 1.3 Hz, 1H), 7.53 (ddd, J = 8.3, 6.9, 1.3 Hz, 1H), 7.34 – 7.29 (m, 2H), 7.13 – 7.09 (m, 2H), 5.56 (p, J = 7.2 Hz, 1H), 2.25 (s, 3H), 1.56 (d, J = 7.0 Hz, 3H). ^13^C NMR (151 MHz, DMSO-*d6*) δ 158.58 (C), 154.99 (CH), 149.19 (C), 141.70 (C), 135.59 (C), 132.52 (CH), 128.76 (CH, 2C), 127.44 (CH), 126.02 (CH, 2C), 125.49 (CH), 122.96 (CH), 114.84 (C), 48.93 (CH), 22.28 (CH_3_), 20.61 (CH_3_).

# NMR spectrums

***N-*benzylthieno[2,3*-d*]pyrimidin-4-amine, 4a**

***
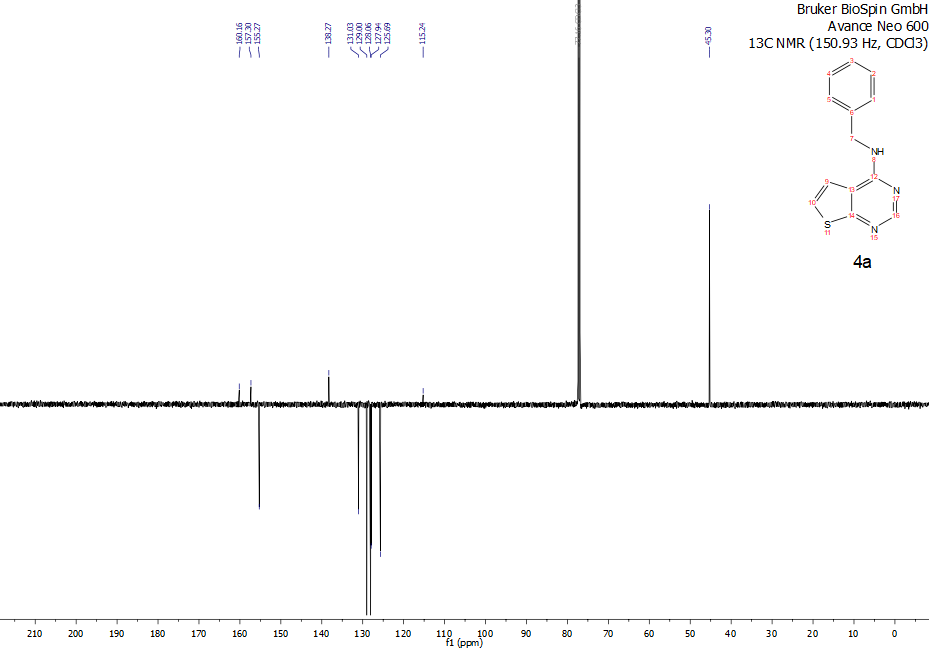

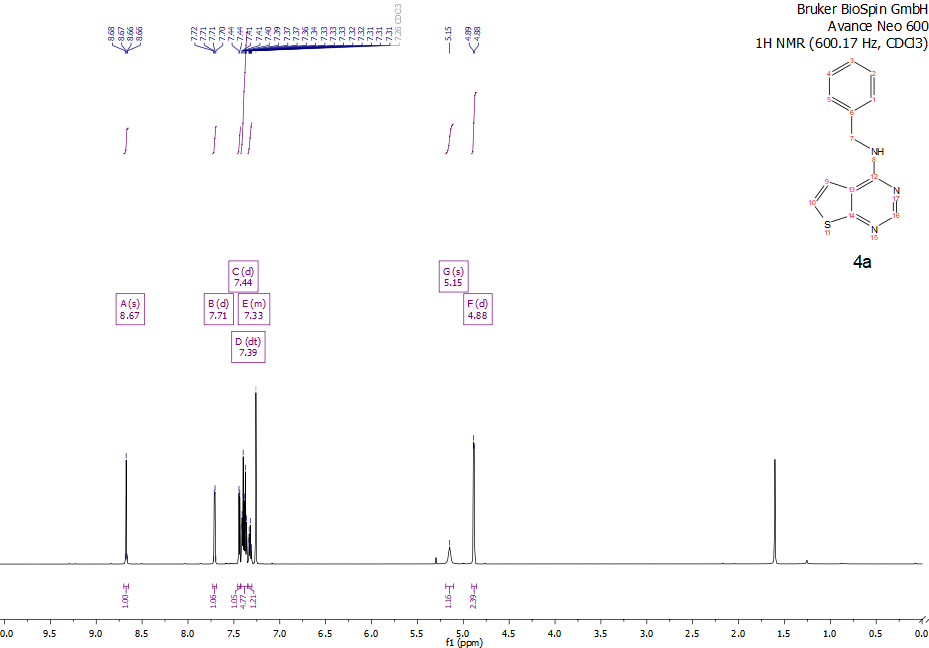
***

***N-*(2-methoxybenzyl)thieno[2,3*-d*]pyrimidin-4-amine, 4b**

***
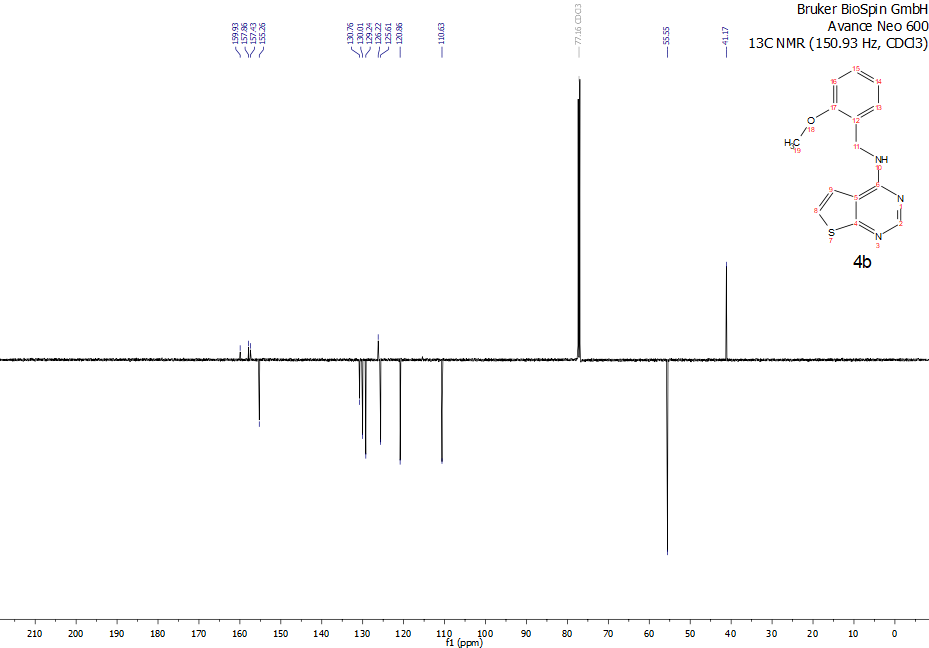
***

***
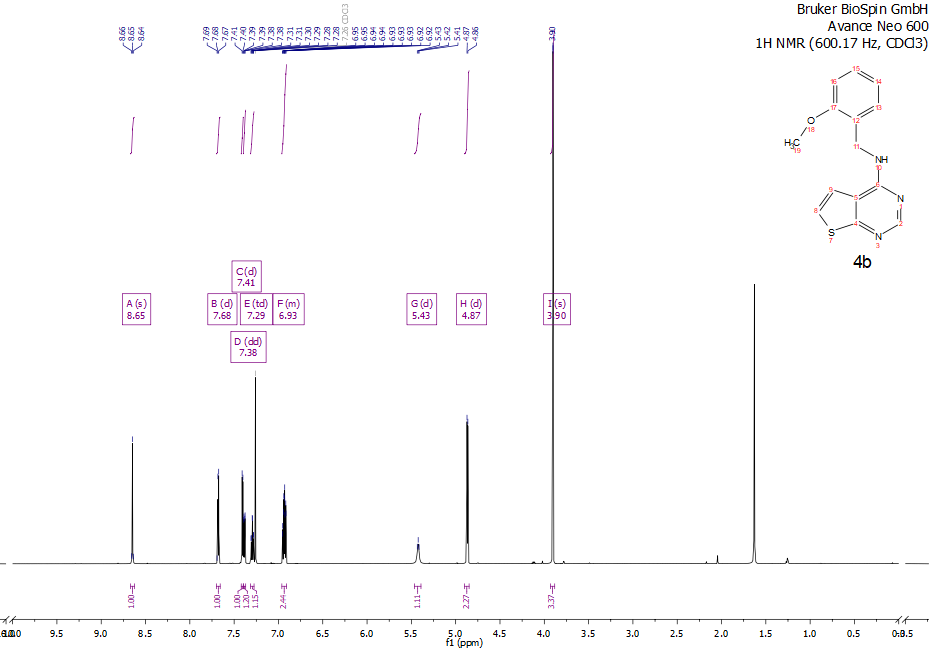
***

***N-*(3-methoxybenzyl)thieno[2,3*-d*]pyrimidin-4-amine, 4c**

***
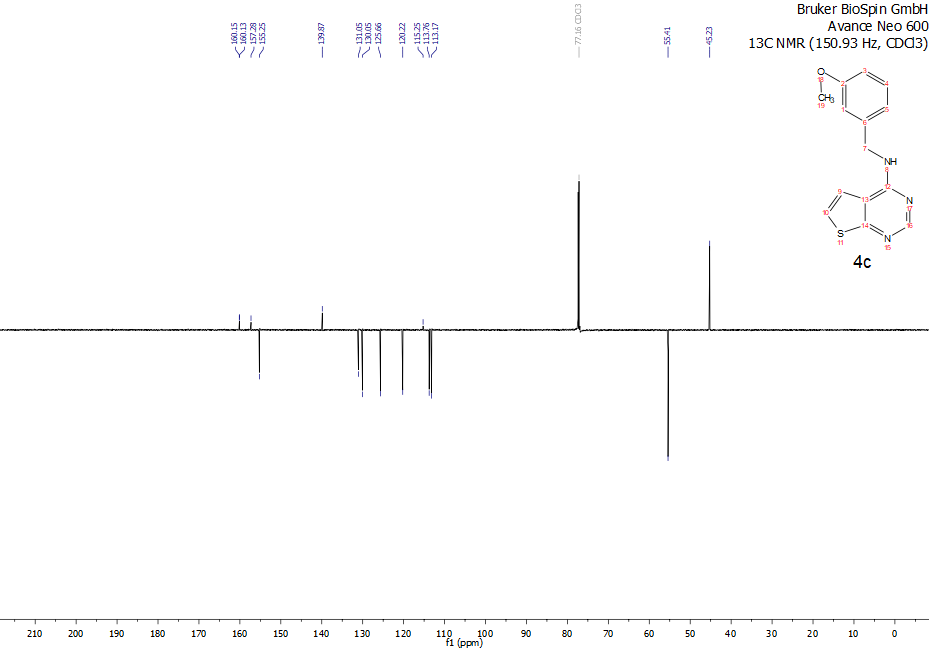

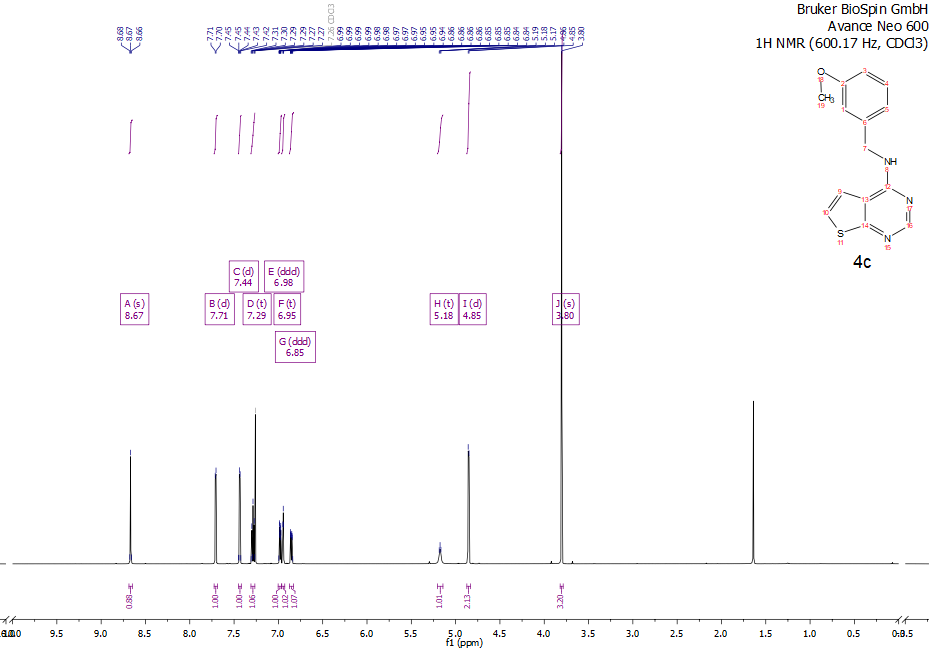
***

***N-*(4-methoxybenzyl)thieno[2,3*-d*]pyrimidin-4-amine, 4d**

**
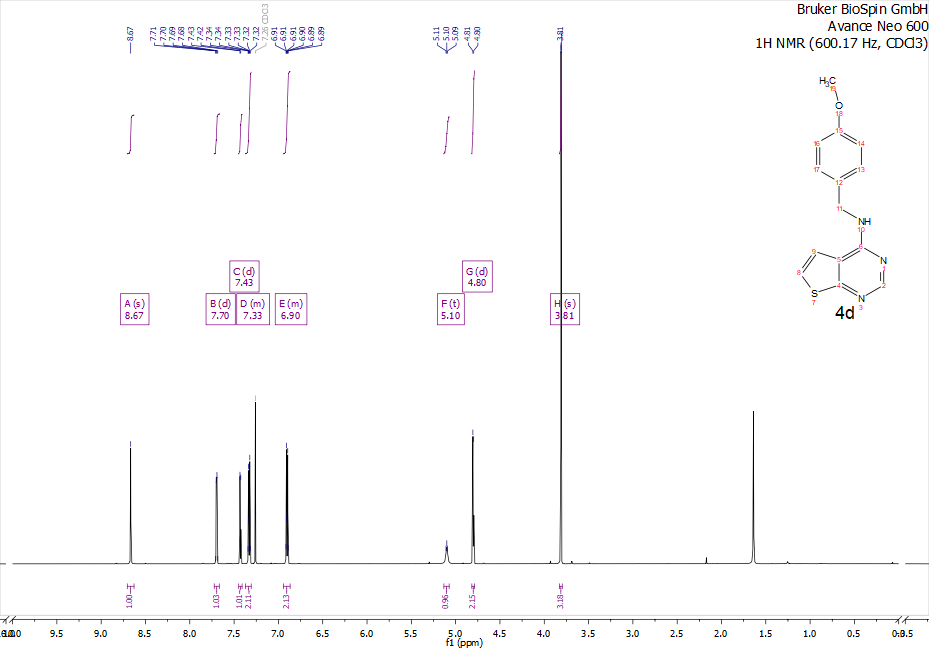
**

**
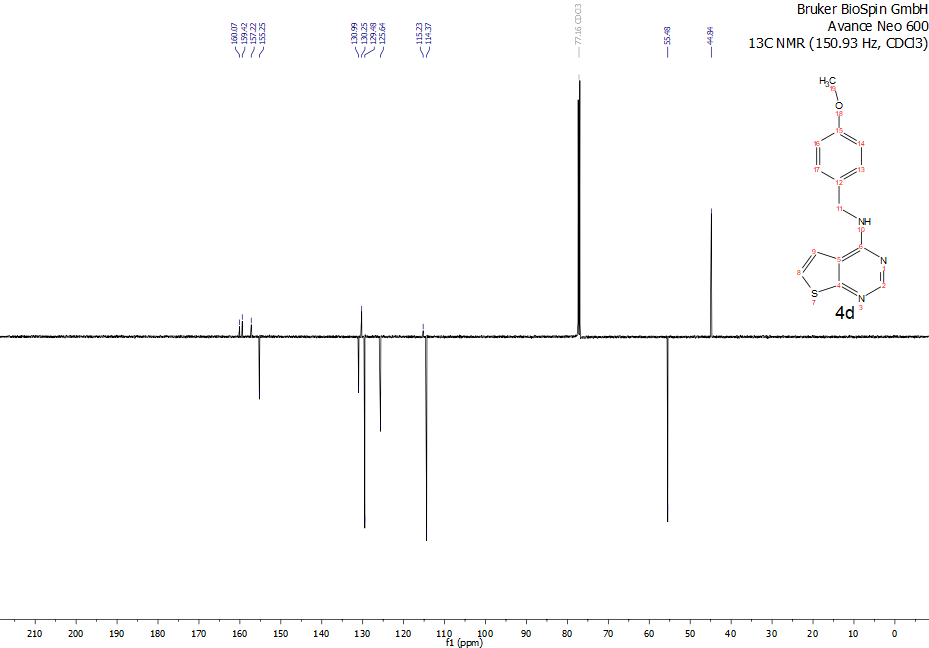
**

***N-*(2-fluorobenzyl)thieno[2,3*-d*]pyrimidin-4-amine, 4e**

***
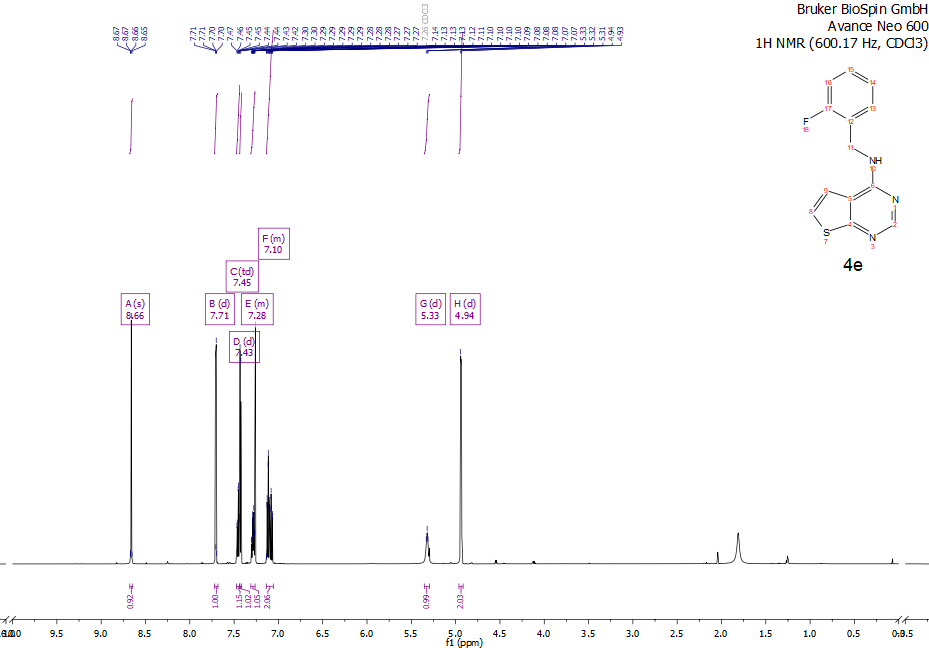
***

***
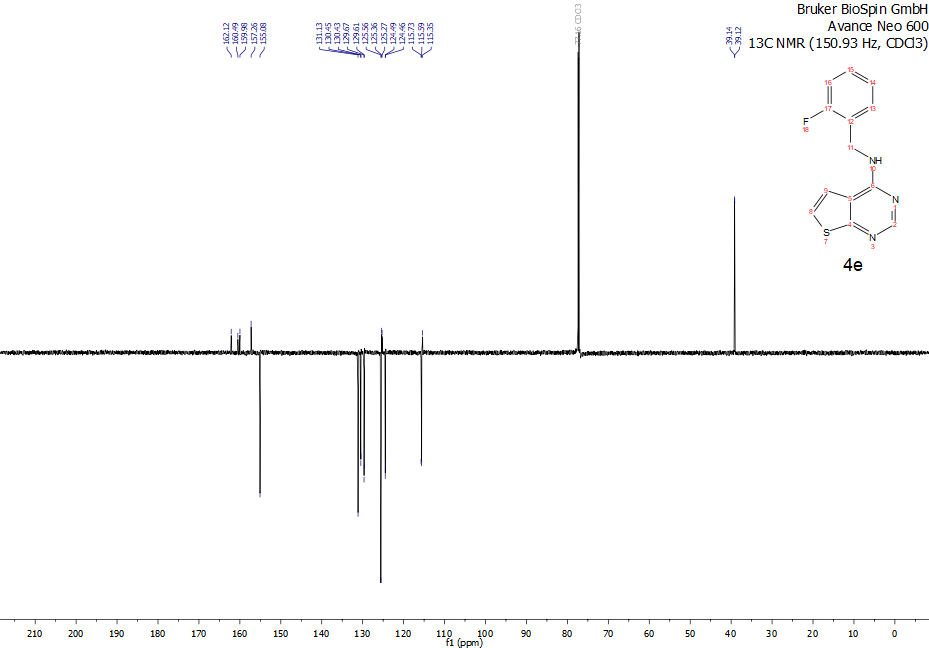
***

***N-*(3-fluorobenzyl)thieno[2,3*-d*]pyrimidin-4-amine, 4f**

***
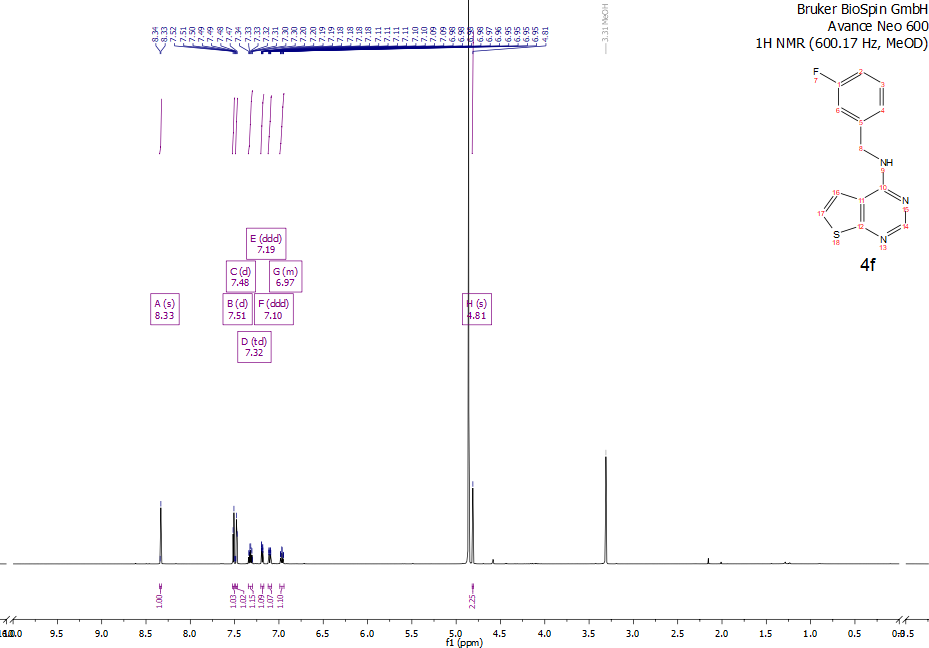
***

***
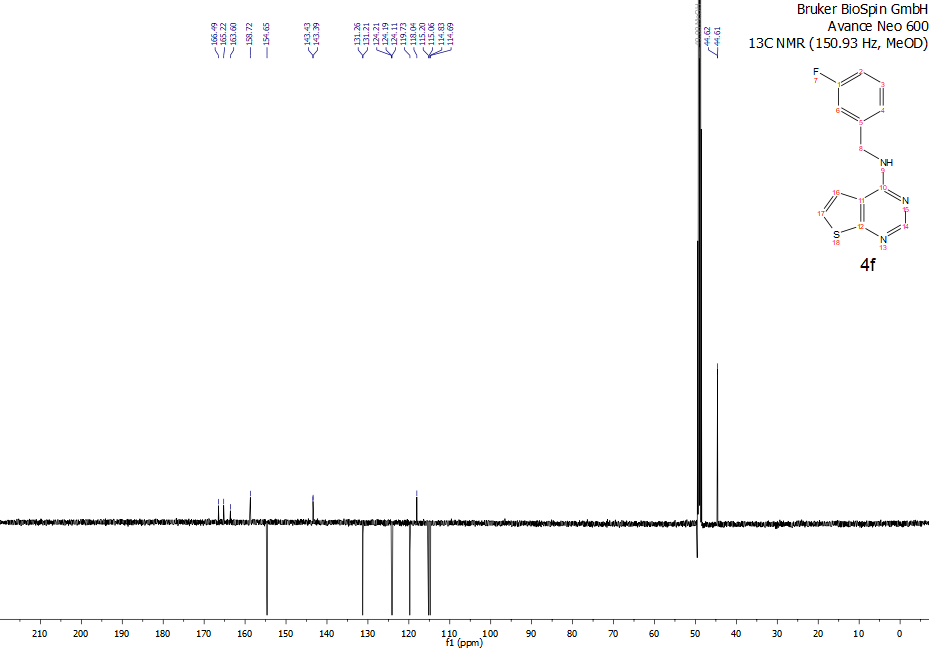
***

***N-*(4-fluorobenzyl)thieno[2,3*-d*]pyrimidin-4-amine, 4g**

***
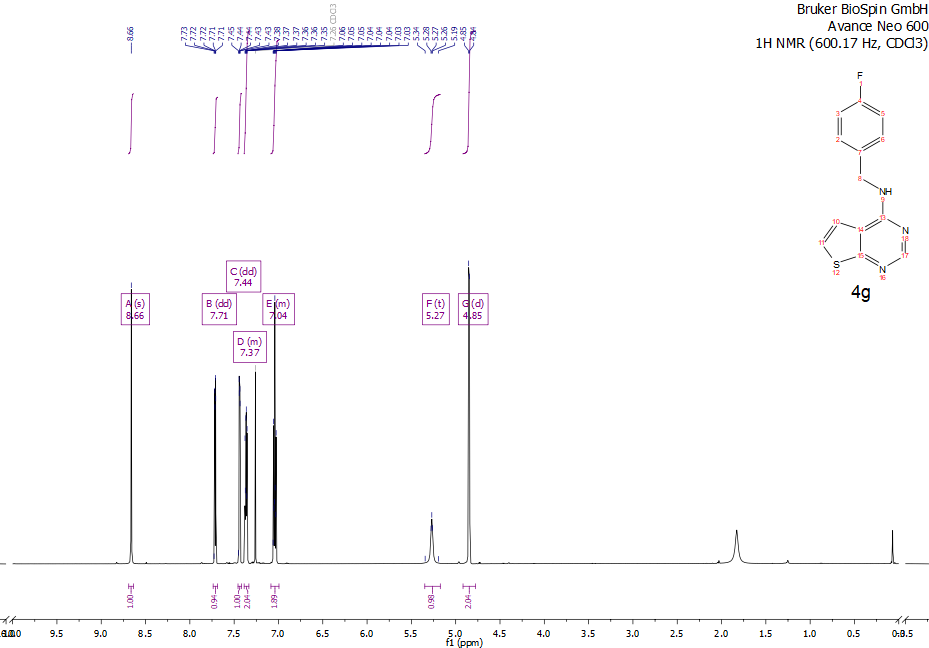
***

***
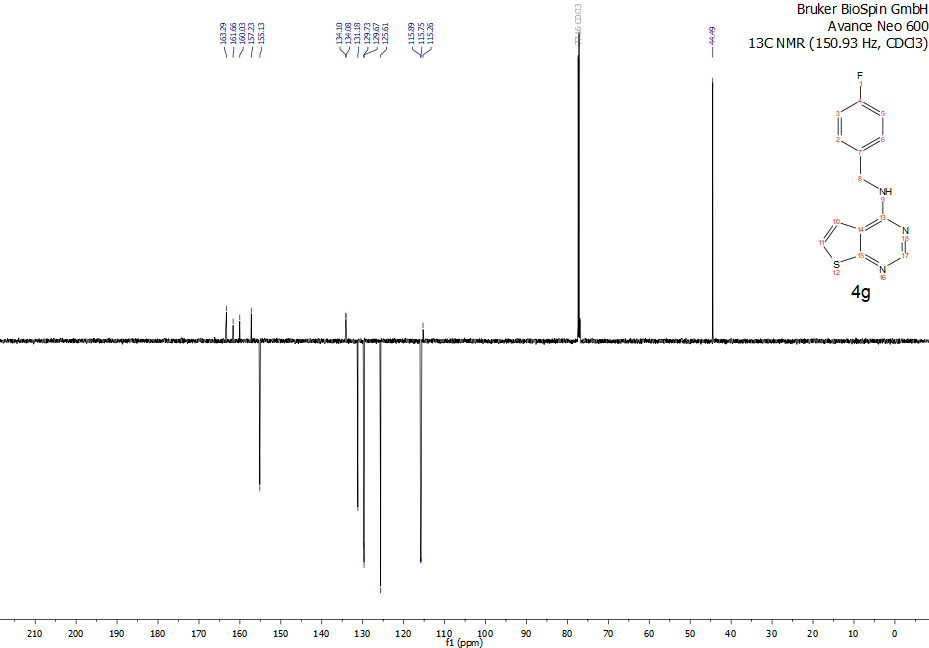
***

***N-*(2-chlorobenzyl)thieno[2,3*-d*]pyrimidin-4-amine, 4h**

***
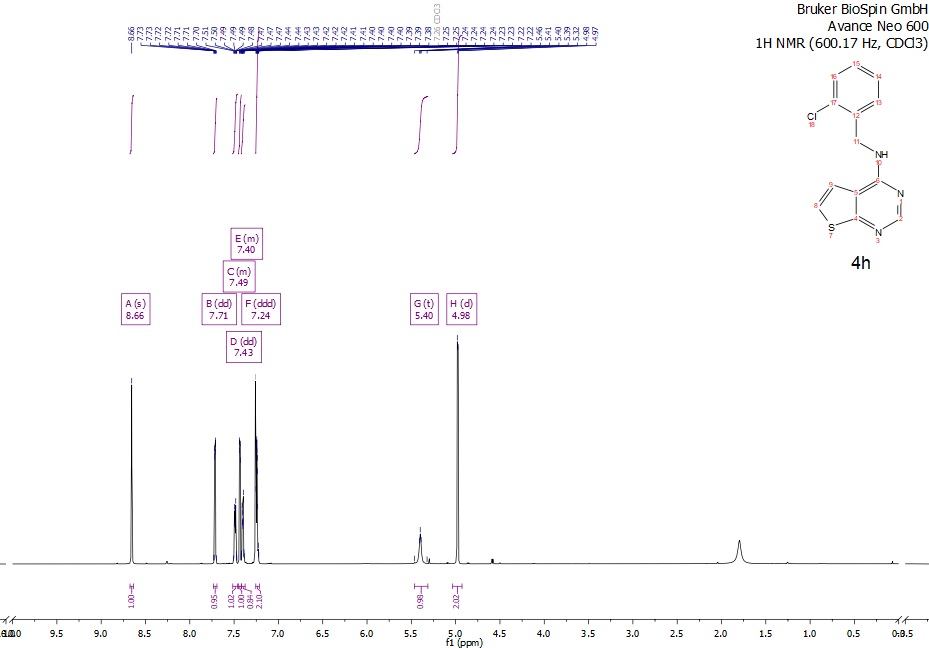
***

***
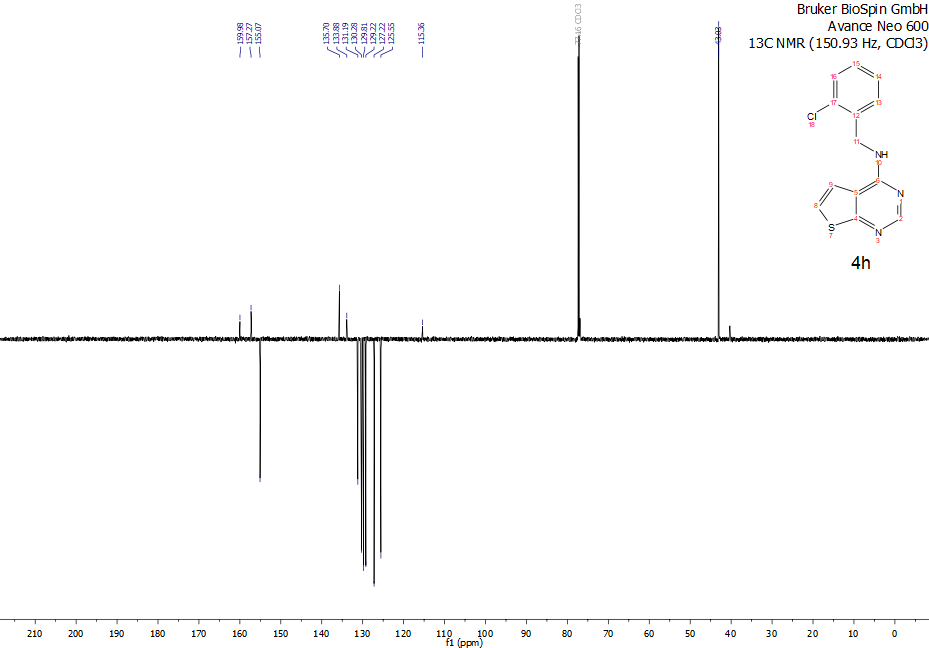
***

***N-*(3-chlorobenzyl)thieno[2,3*-d*]pyrimidin-4-amine, 4i**

***
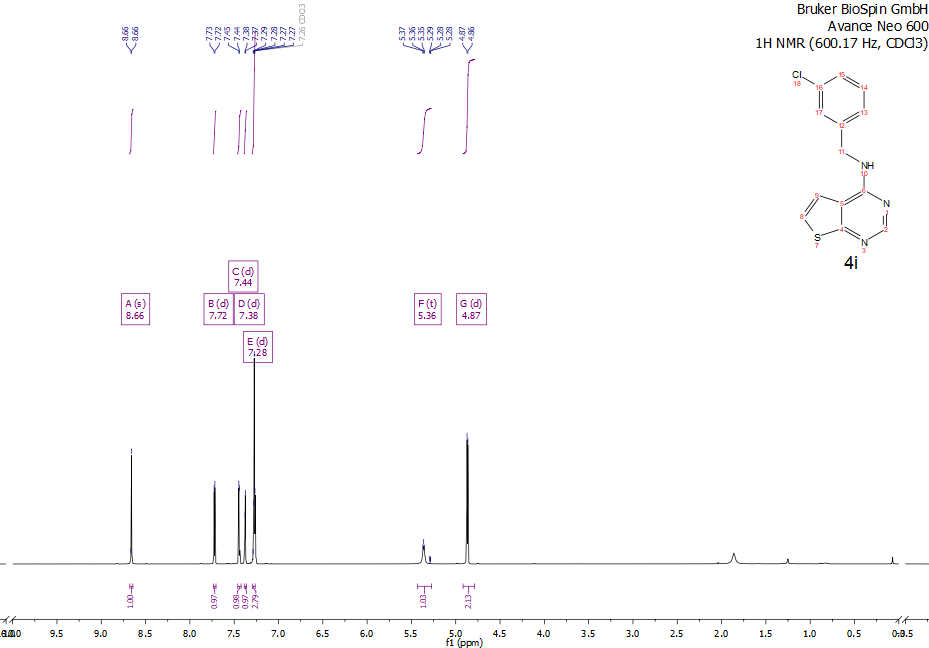
***

***
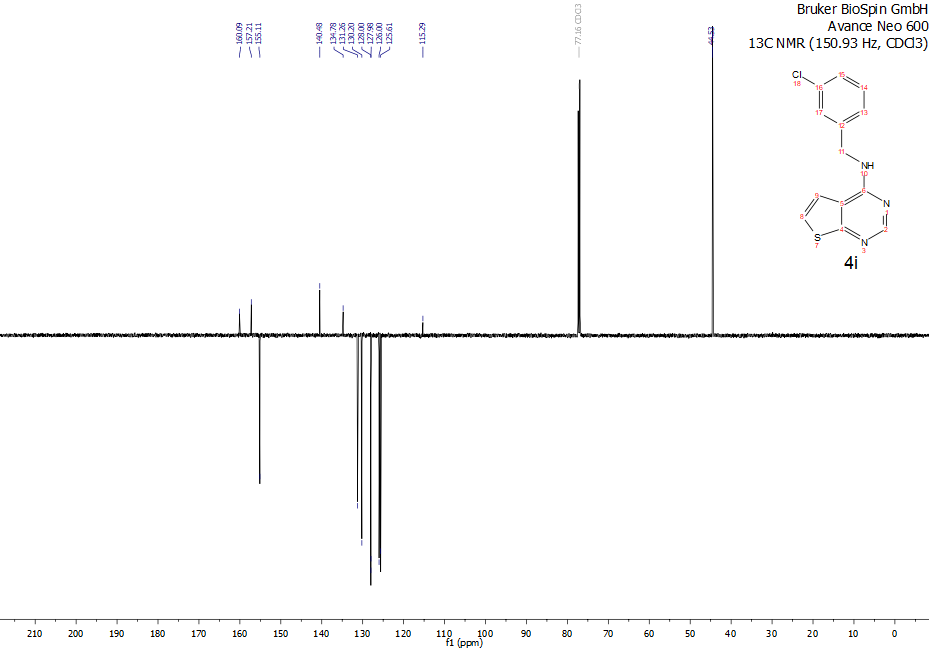
***

***N-*(4-chlorobenzyl)thieno[2,3*-d*]pyrimidin-4-amine, 4j**

**
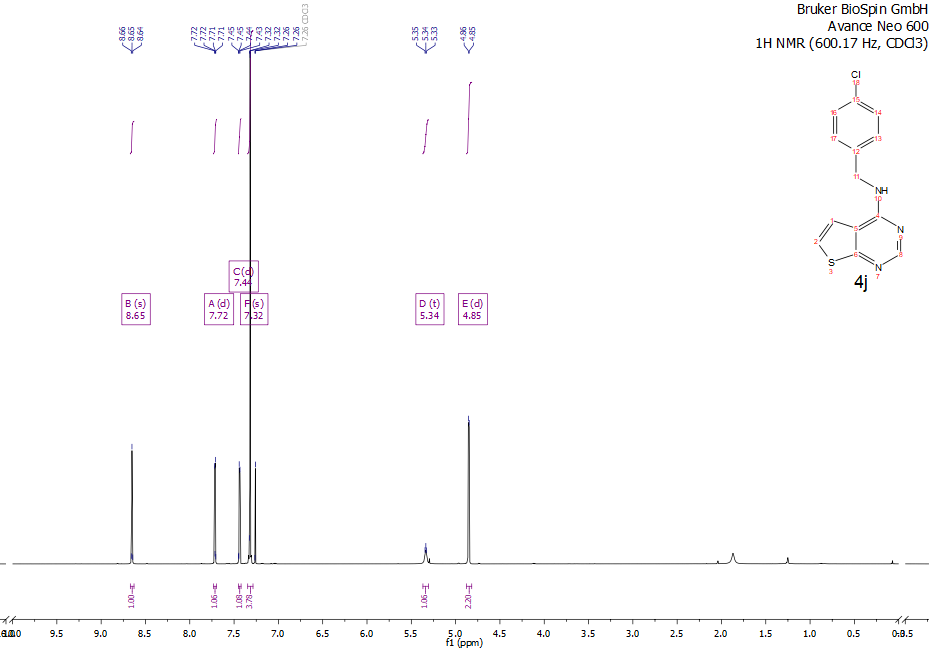
**

**
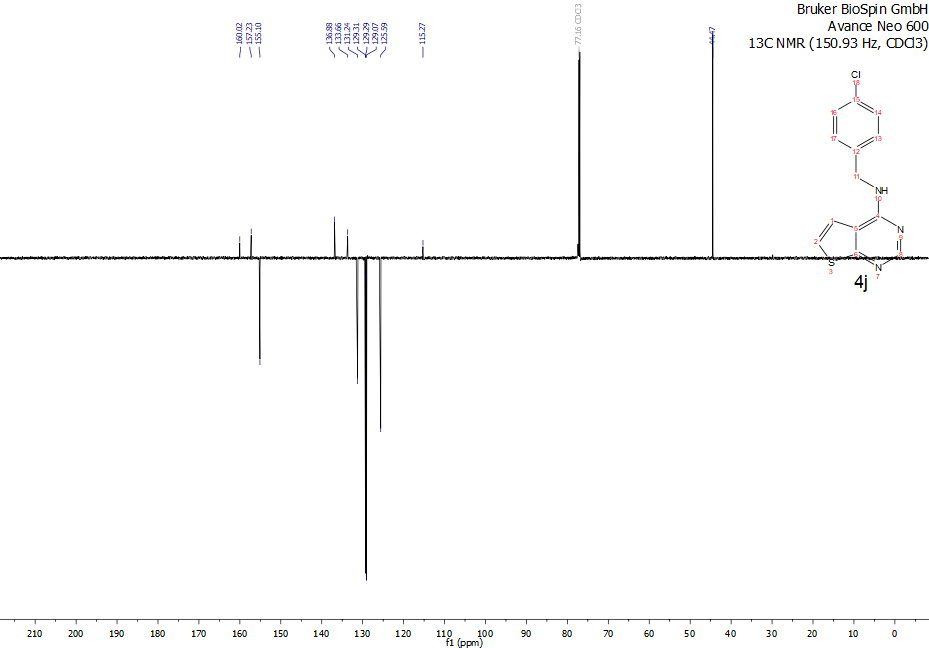
**

**2-((thieno[2,3*-d*]pyrimidin-4-ylamino)methyl)benzonitrile, 4k**

**
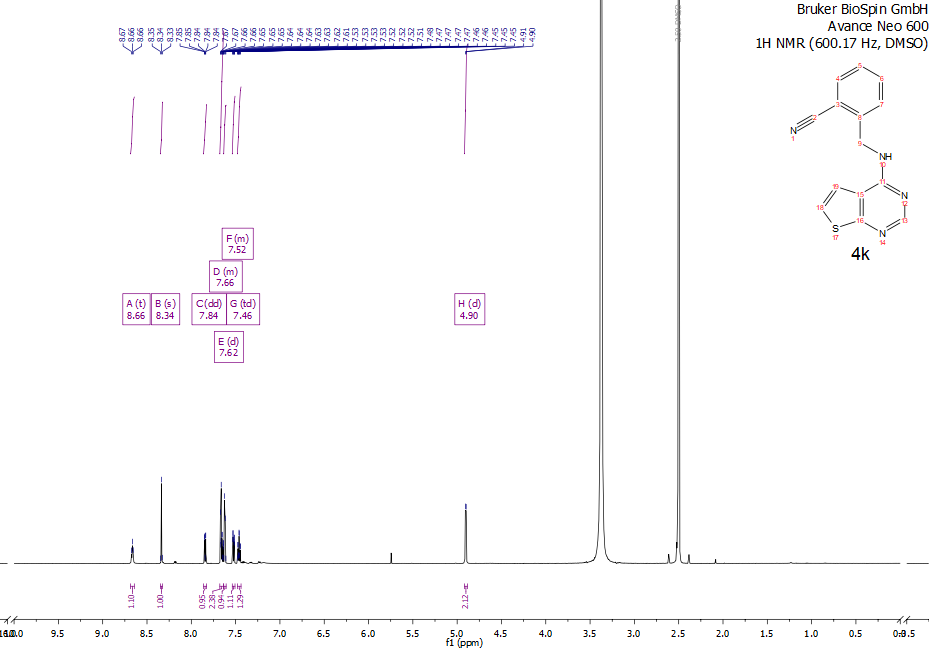
**

**
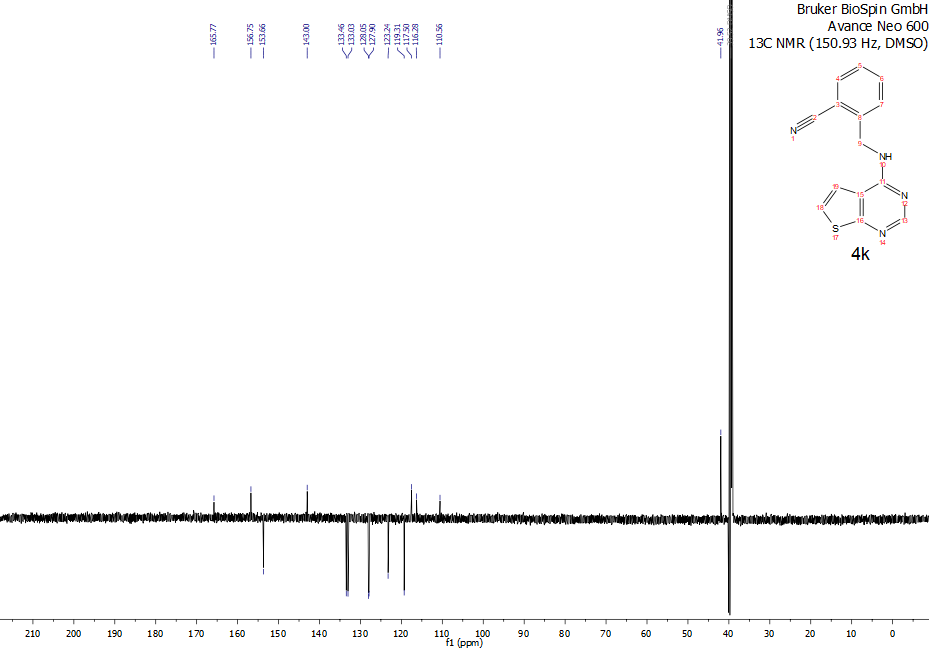
**

**3-((thieno[2,3*-d*]pyrimidin-4-ylamino)methyl)benzonitrile, 4l**

**
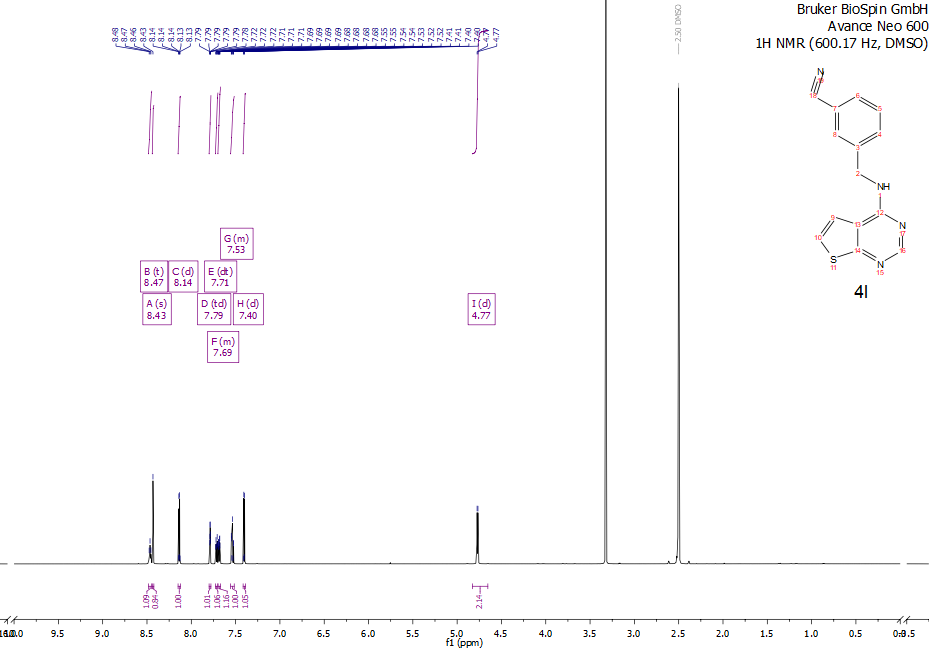
**

**
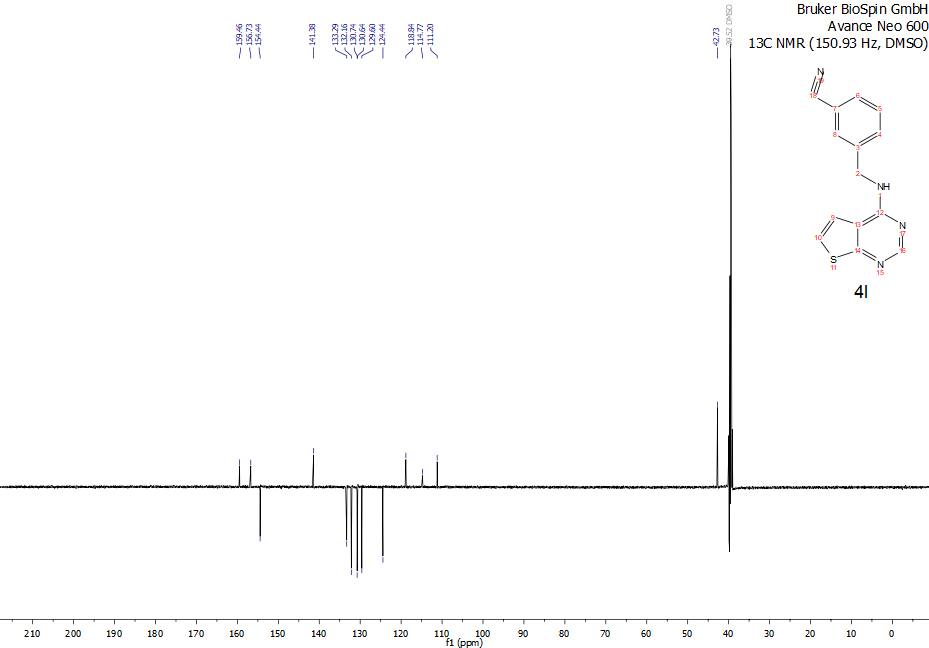
**

**4-((thieno[2,3*-d*]pyrimidin-4-ylamino)methyl)benzonitrile, 4m**

***
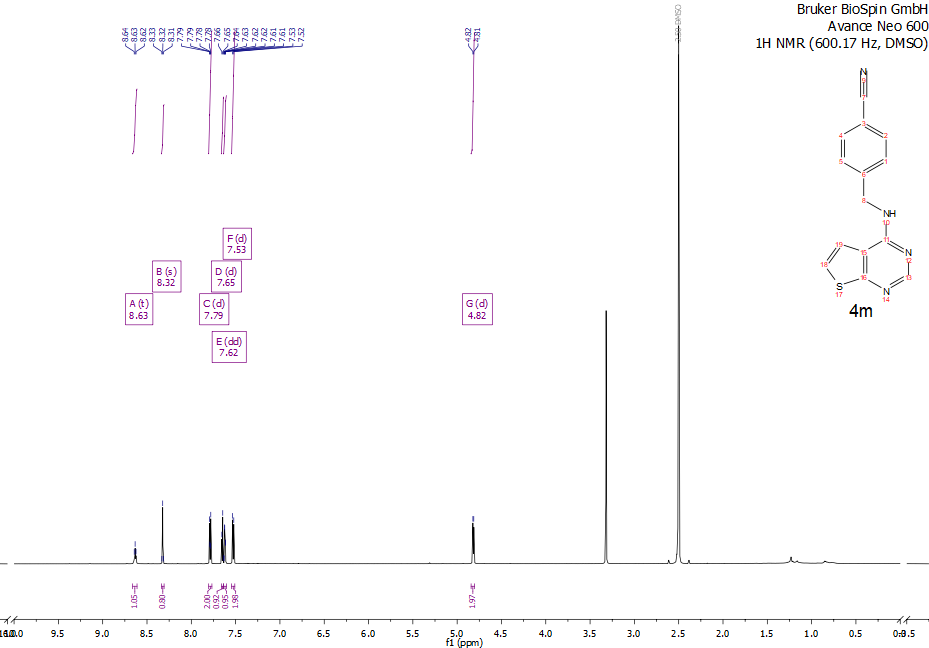
***

***
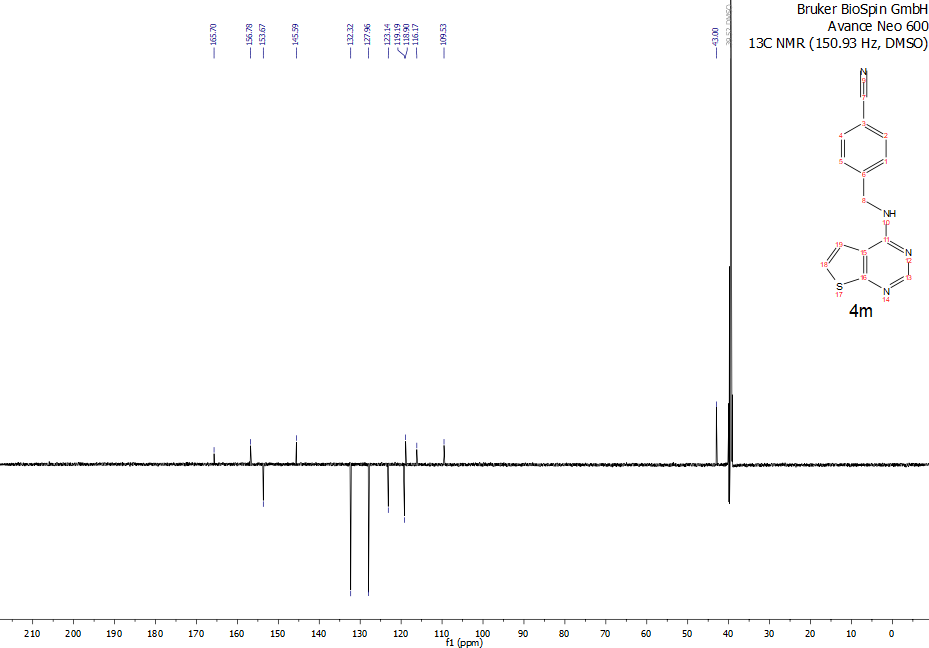
***

***N-*(3-methylbenzyl)thieno[2,3*-d*]pyrimidin-4-amine, 4n**

***
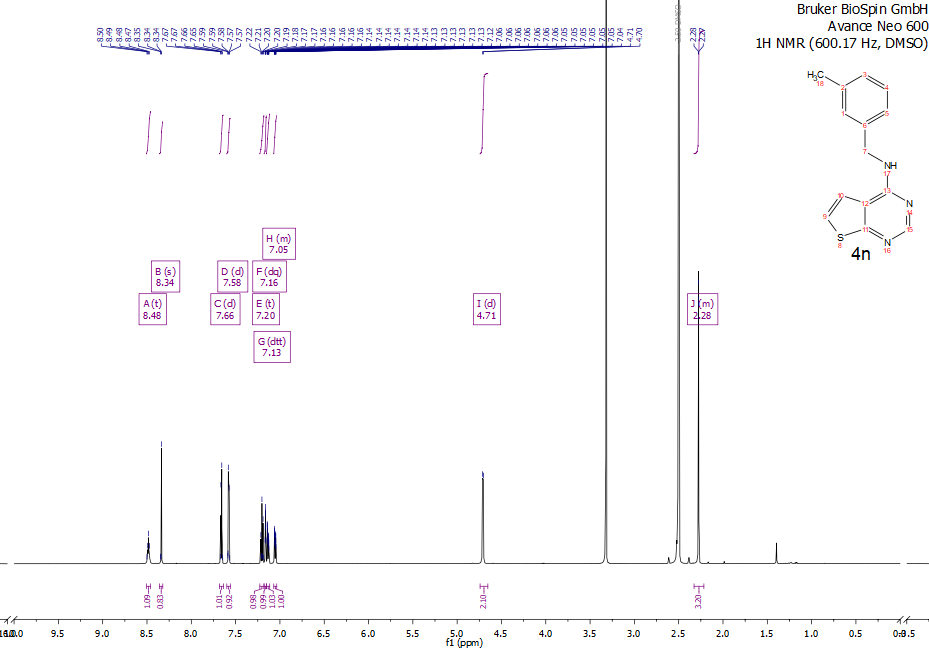
***

***
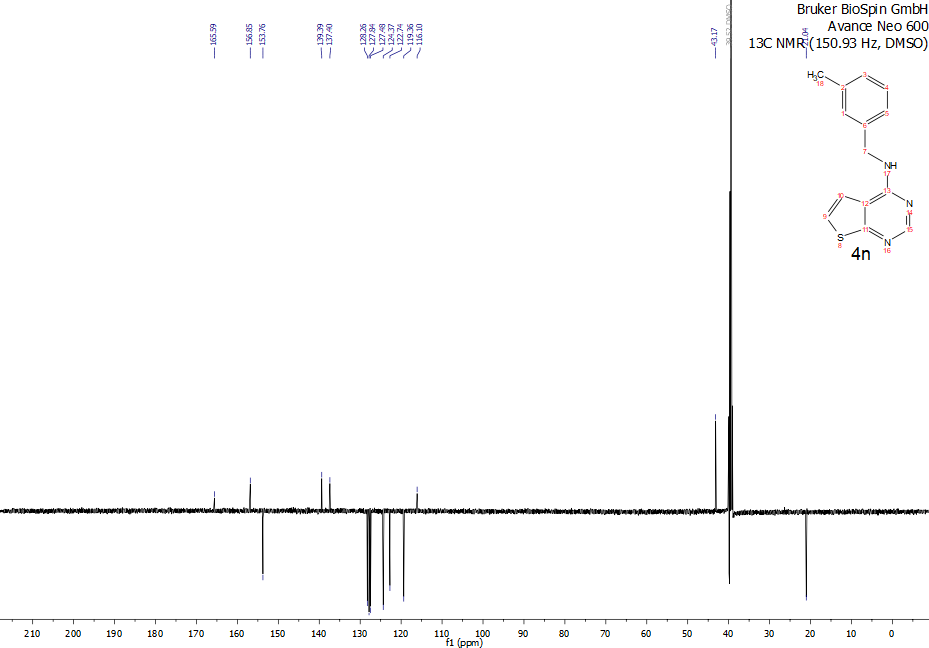
***

***N-*(4-methylbenzyl)thieno[2,3*-d*]pyrimidin-4-amine, 4o**

***
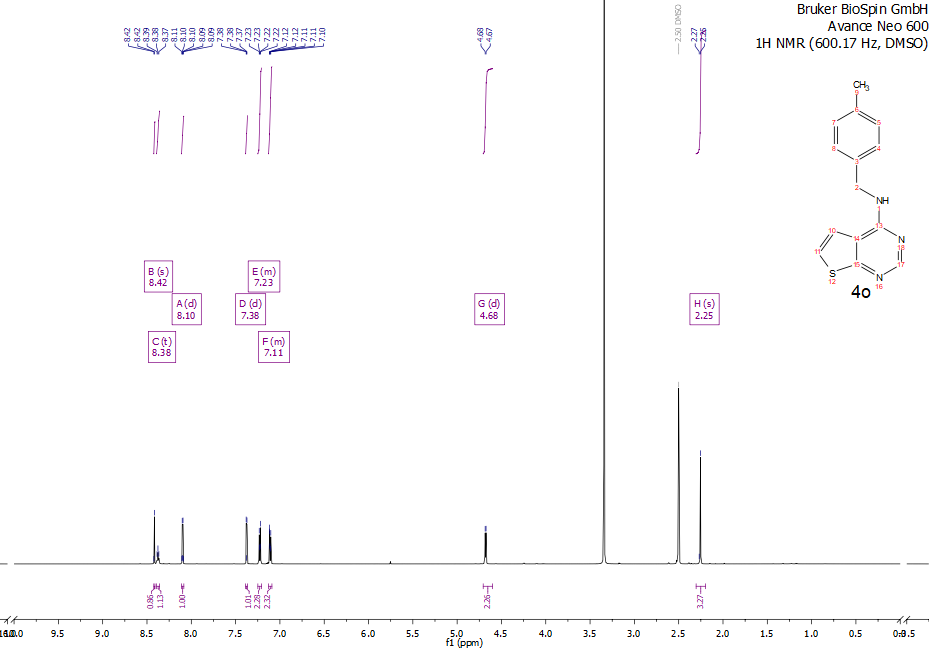
***

***
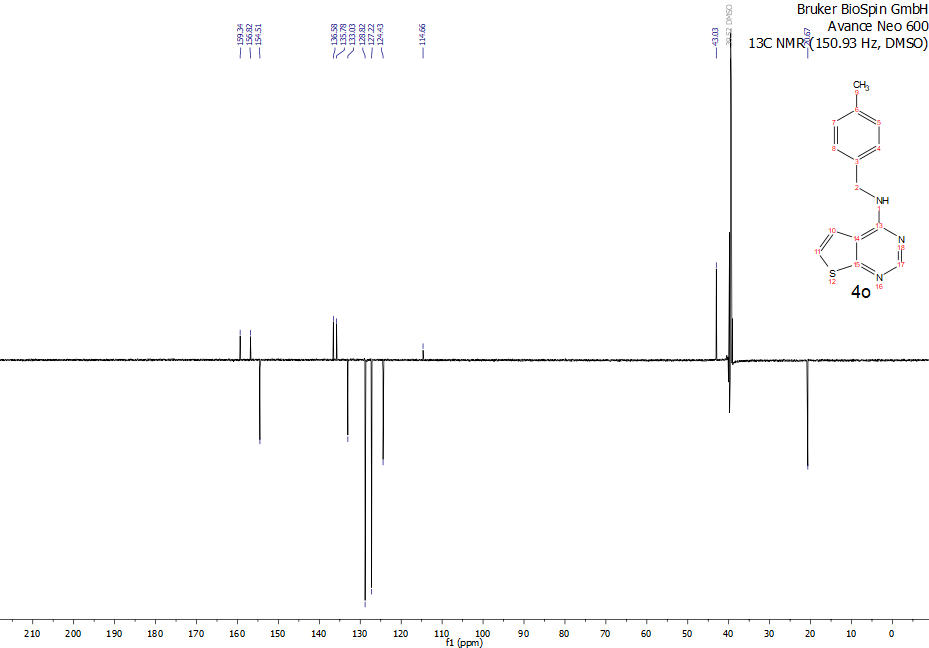
***

***N-*(4-fluoro-3-methoxybenzyl)thieno[2,3*-d*]pyrimidin-4-amine, 4p**

***
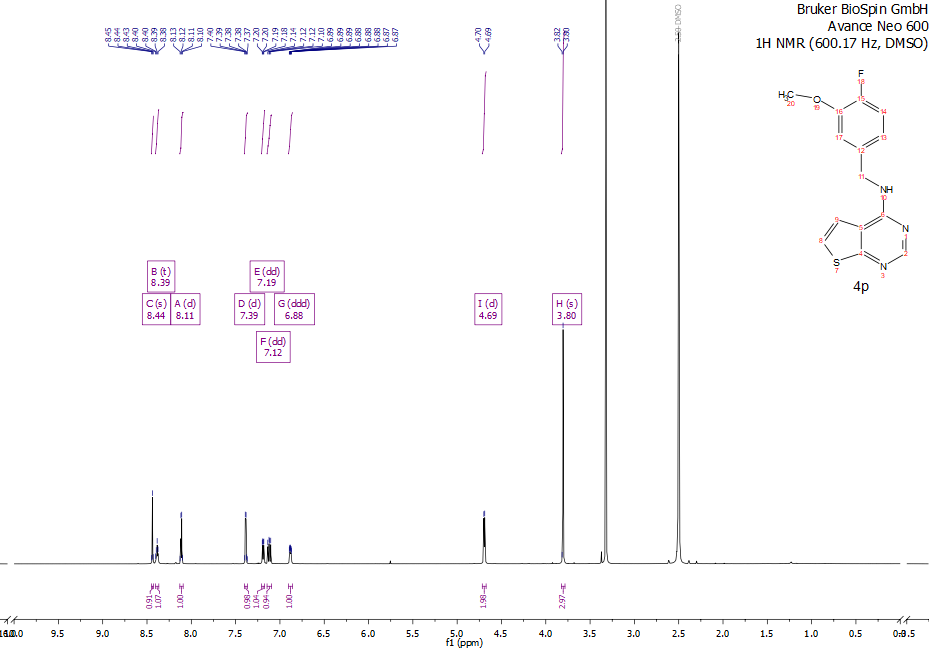
***

***
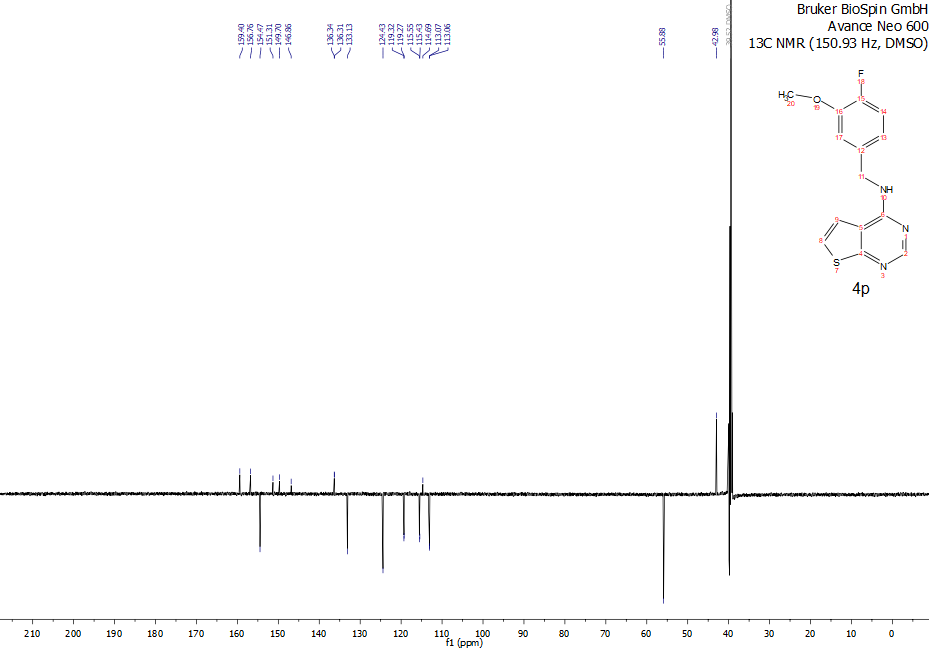
***

***N-*(4-chloro-3-methoxybenzyl)thieno[2,3*-d*]pyrimidin-4-amine, 4q**

***
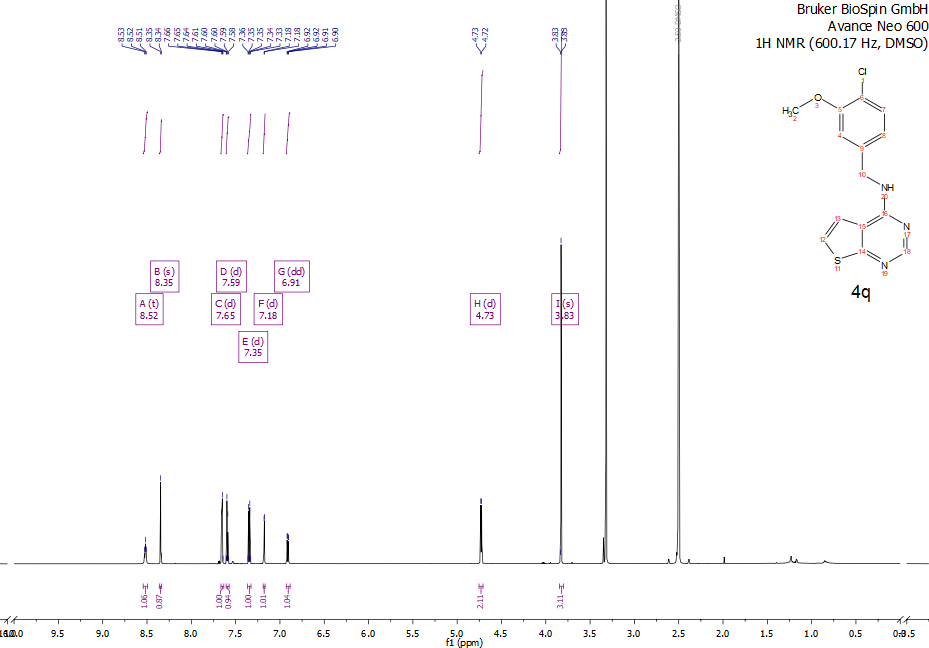
***

***
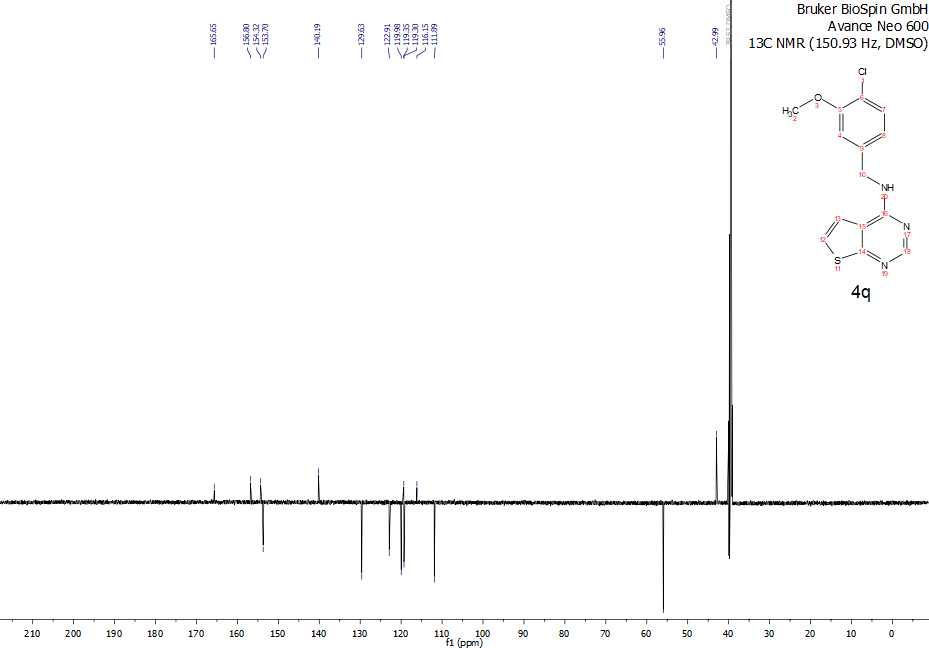
***

***N-*(1-(2-methoxyphenyl)ethyl)thieno[2,3*-d*]pyrimidin-4-amine, 5a**

***
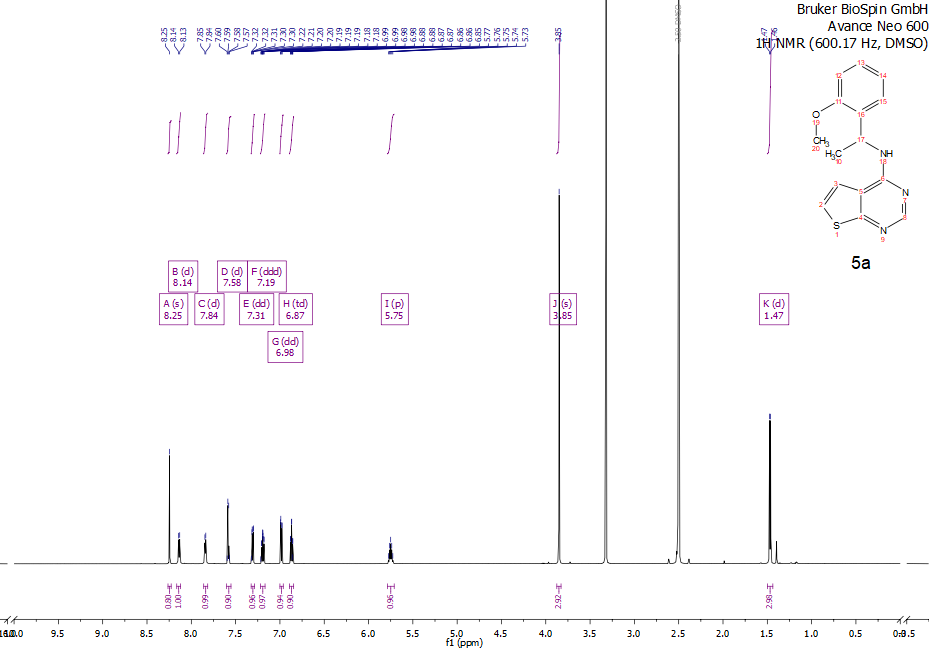
***

***
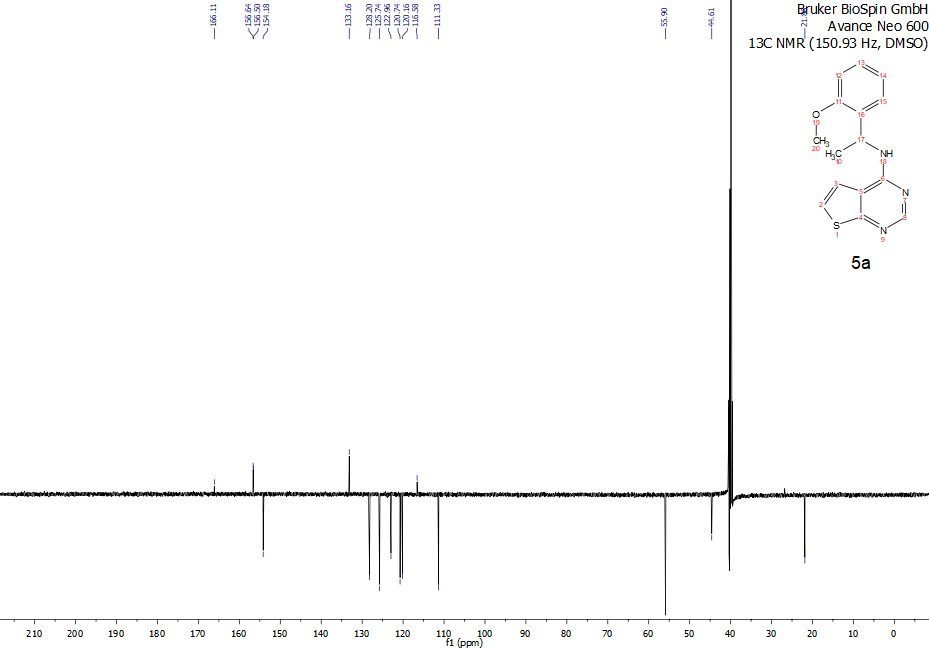
***

***N-*(1-(3-methoxyphenyl)ethyl)thieno[2,3*-d*]pyrimidin-4-amine, 5b**

***
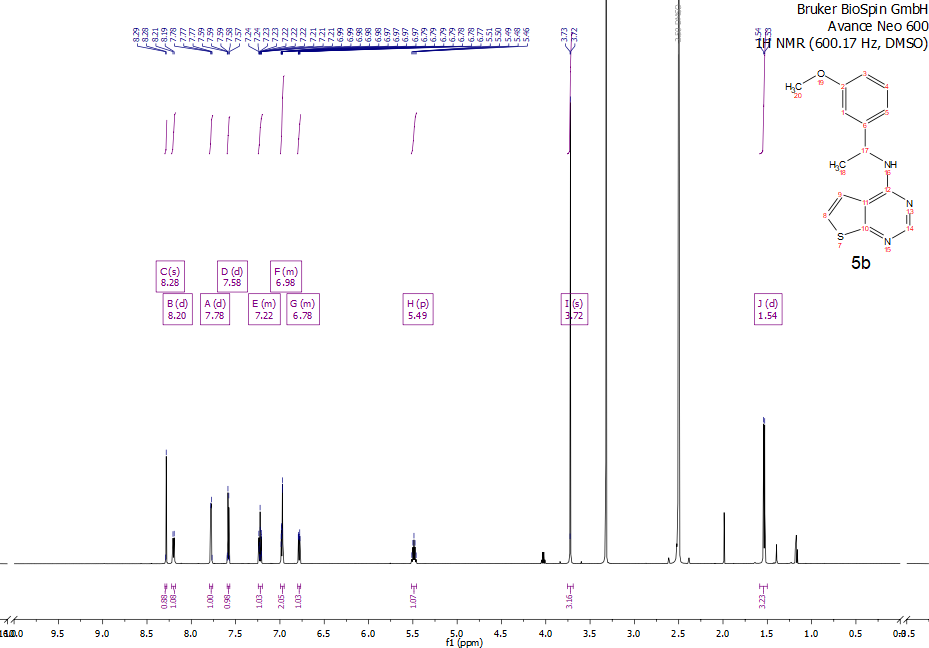
***

***
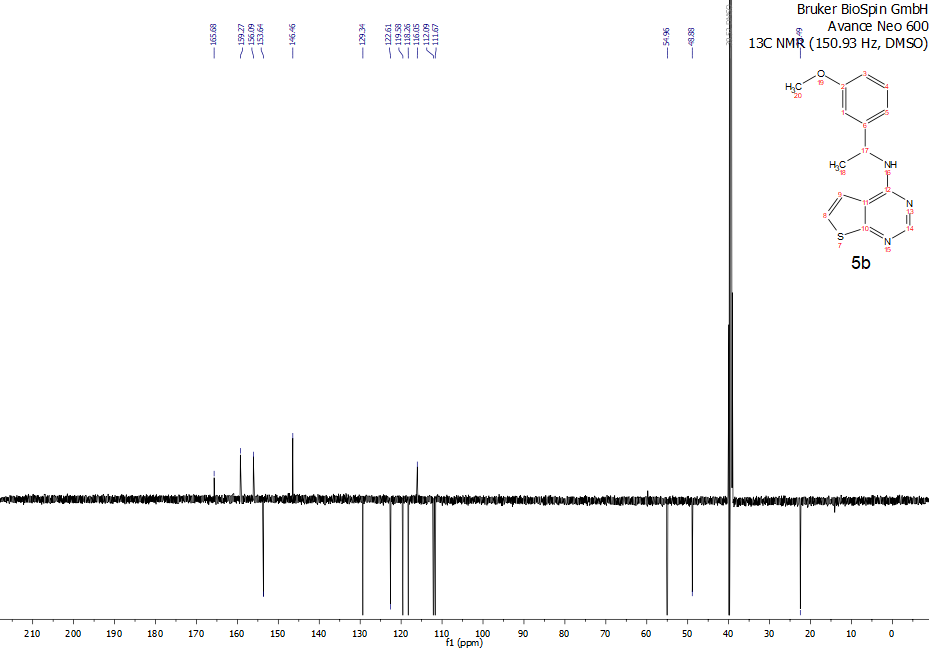
***

***N-*(1-(4-fluorophenyl)ethyl)thieno[2,3*-d*]pyrimidin-4-amine, 5c**

***
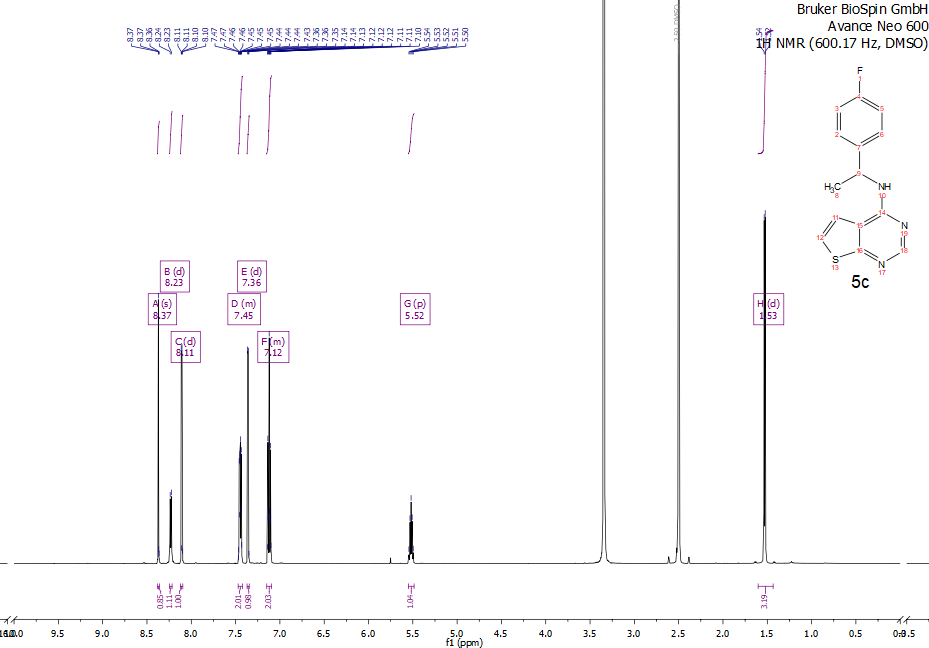
***

***
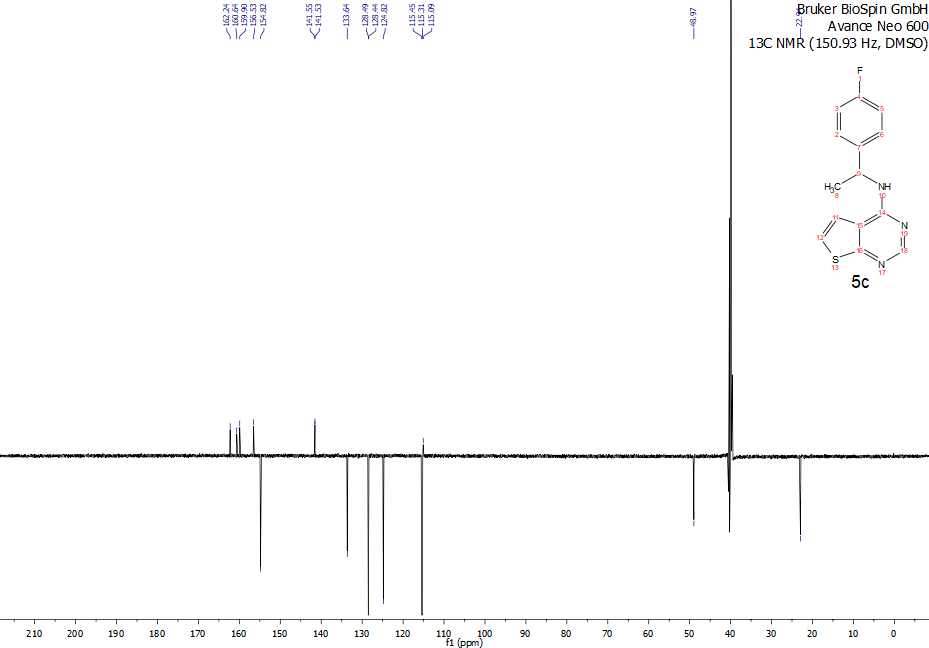
***

***N-*(1-(4-chlorophenyl)ethyl)thieno[2,3*-d*]pyrimidin-4-amine, 5d**

***
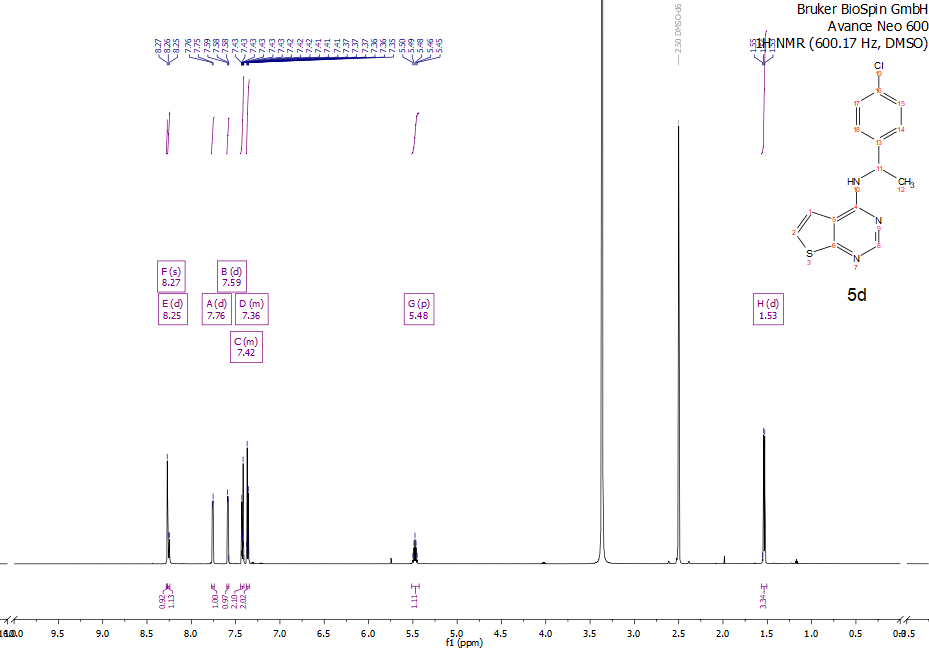

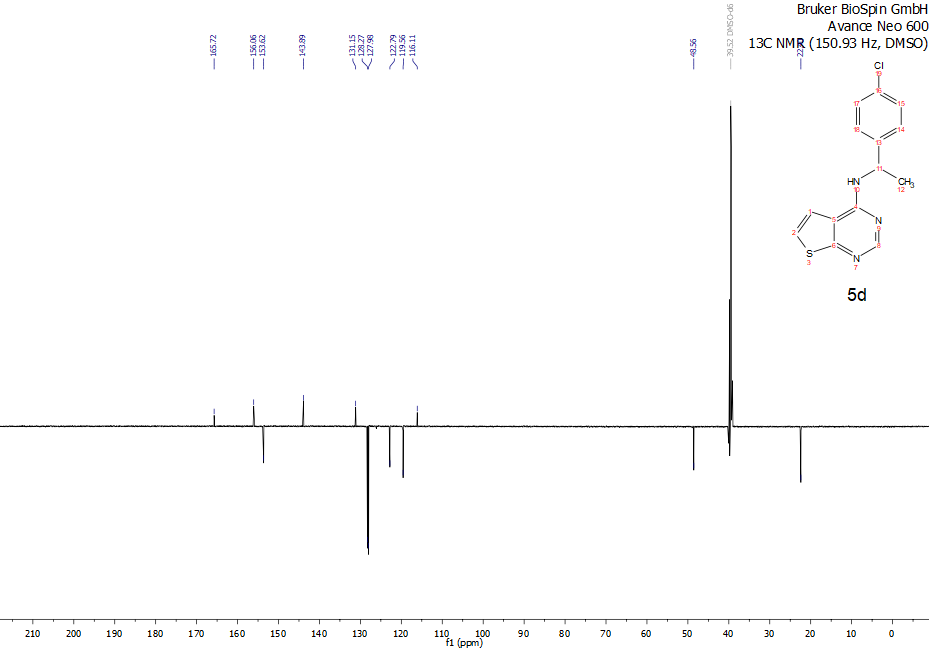
***

***N-*(1-(p-tolyl)ethyl)thieno[2,3*-d*]pyrimidin-4-amine, 5e**

***
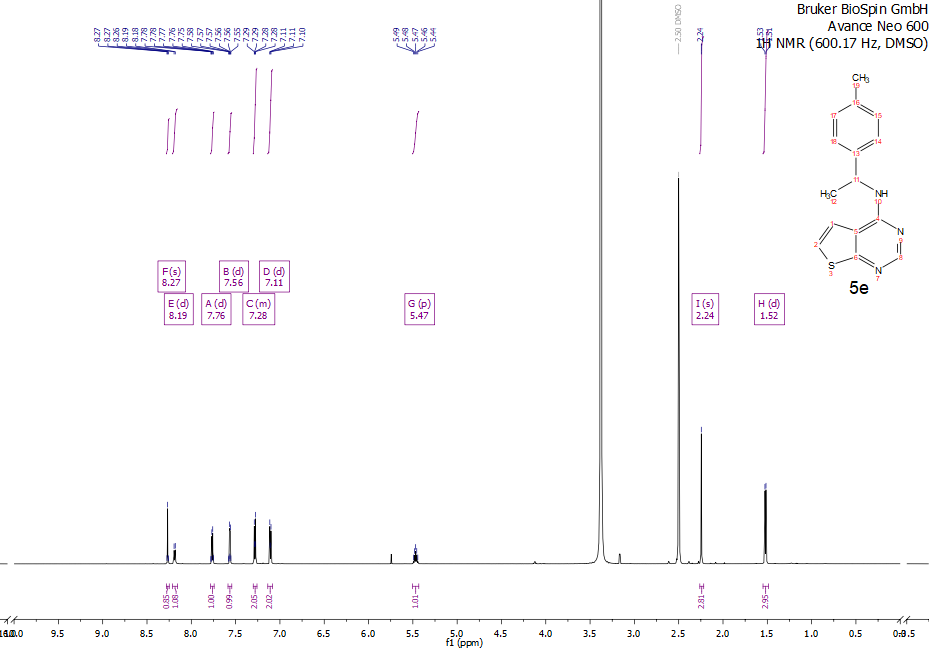
***

***
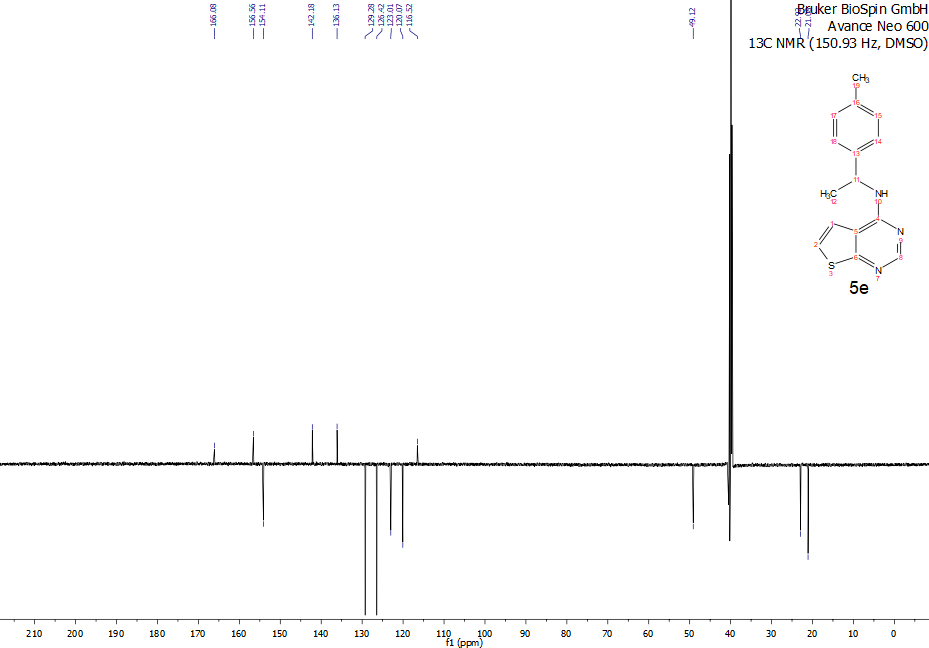
***

***N-*phenylthieno[2,3*-d*]pyrimidin-4-amine, 5f**

***
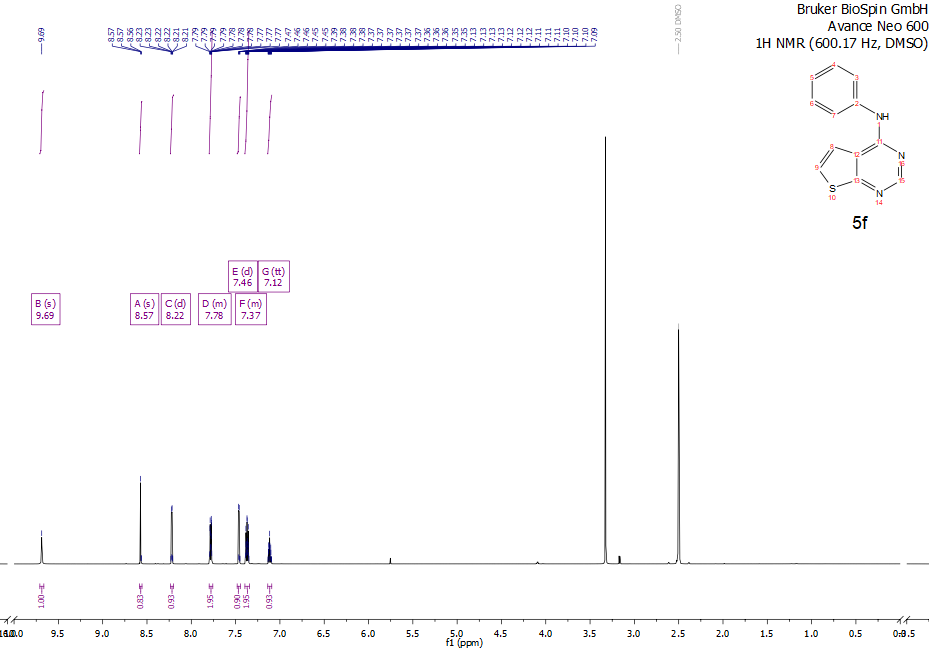
***

***
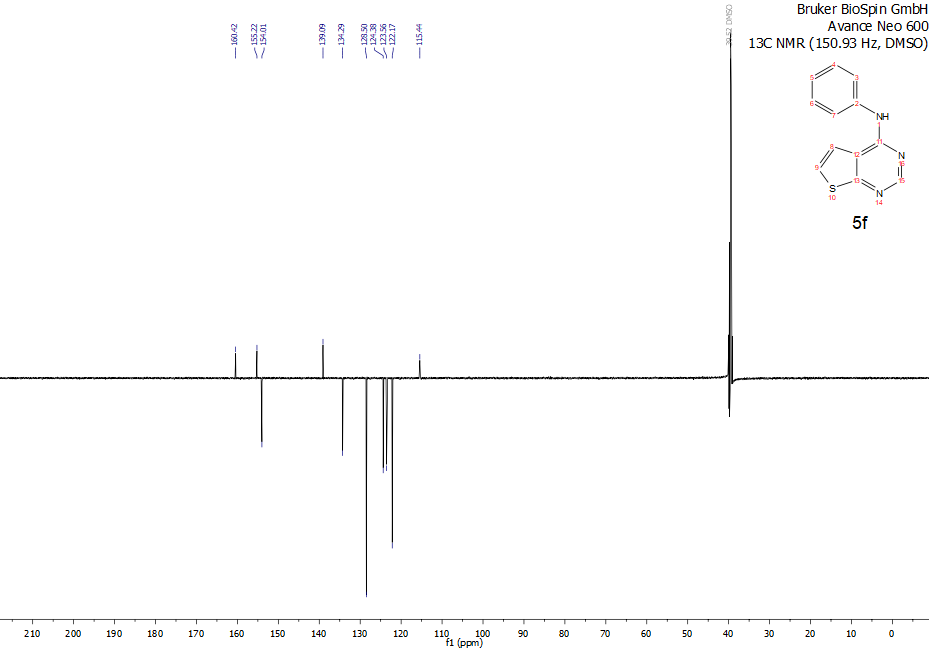
***

***N-*phenethylthieno[2,3*-d*]pyrimidin-4-amine, 5g**

***
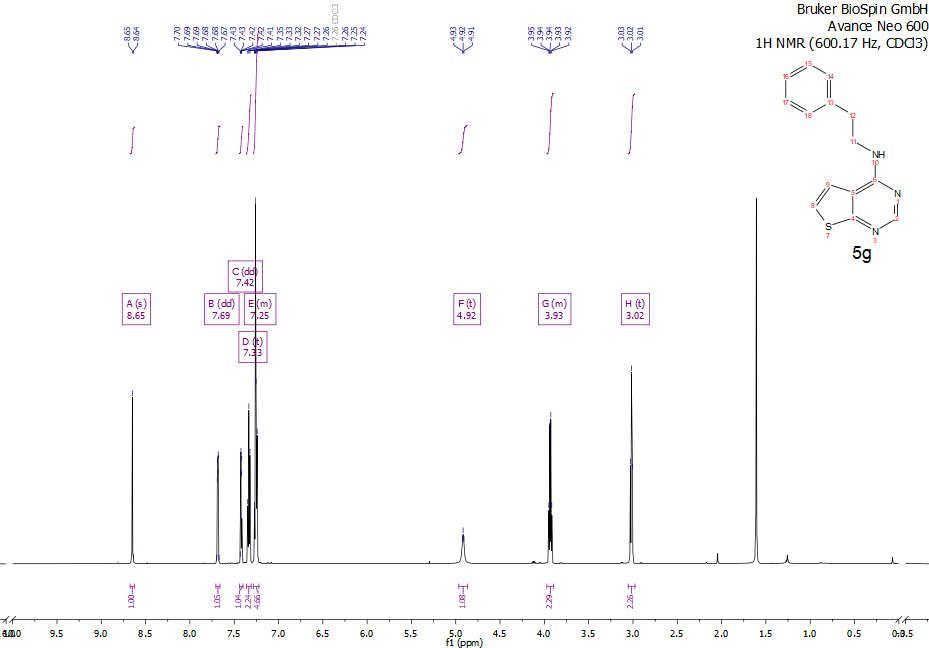
***

***
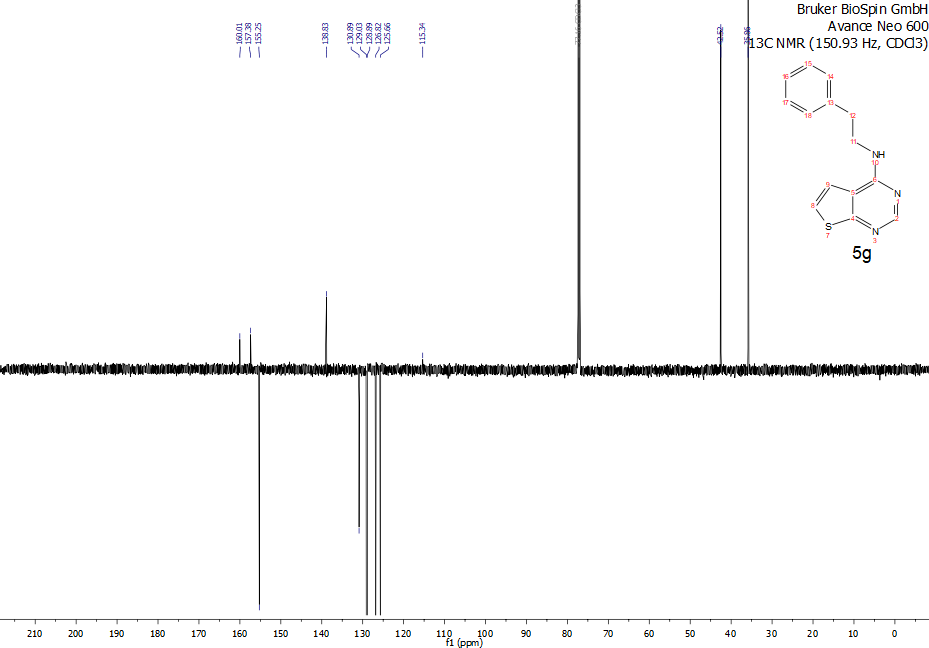
***

***N-*(3-phenylpropyl)thieno[2,3*-d*]pyrimidin-4-amine, 5h**

***
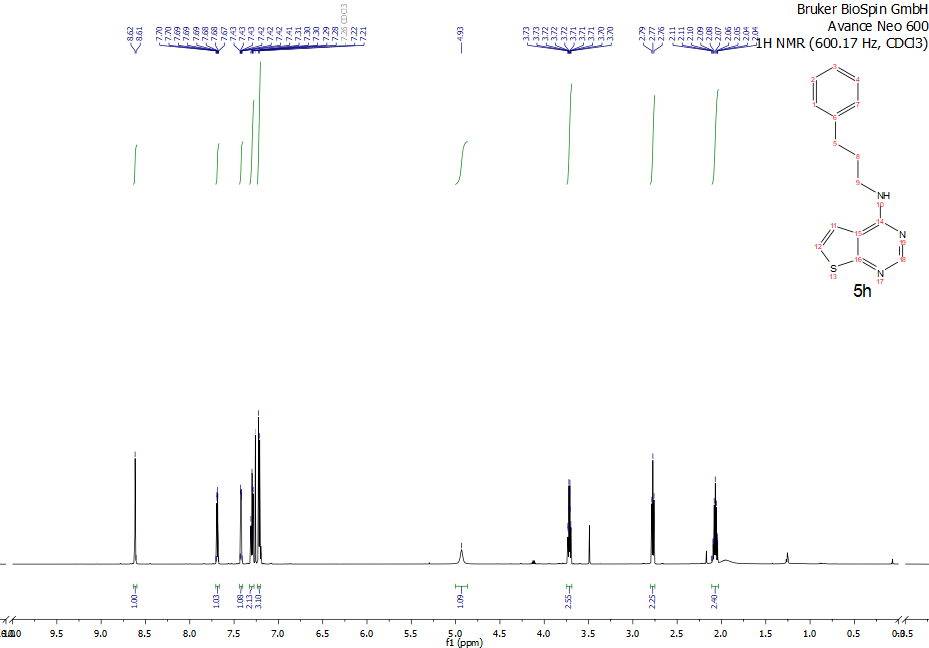
***

***
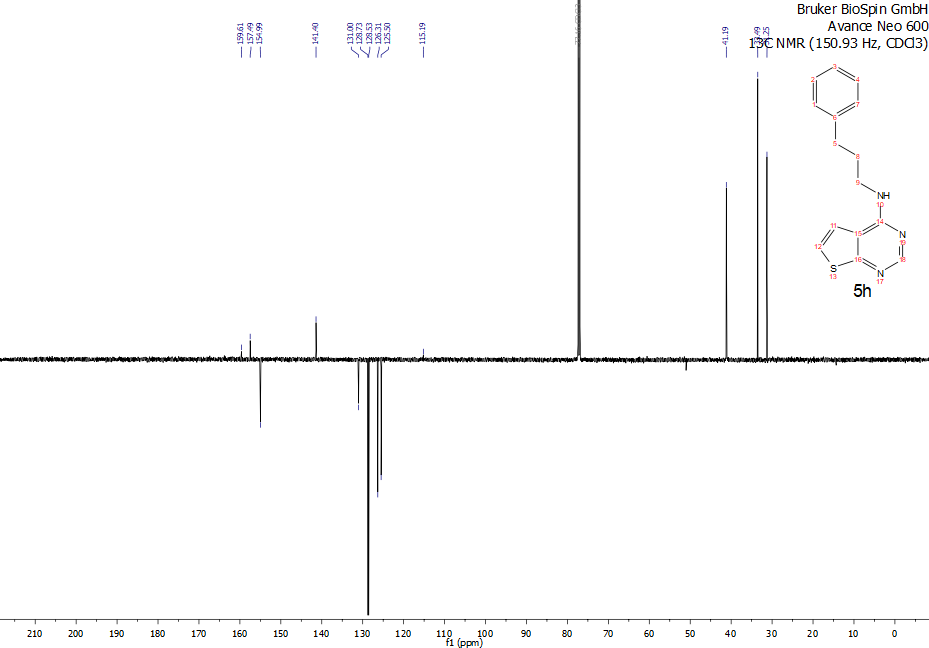
***

***N-*ethyl-*N-*(3-methoxybenzyl)thieno[2,3-d]pyrimidin-4-amine, 5i**

***
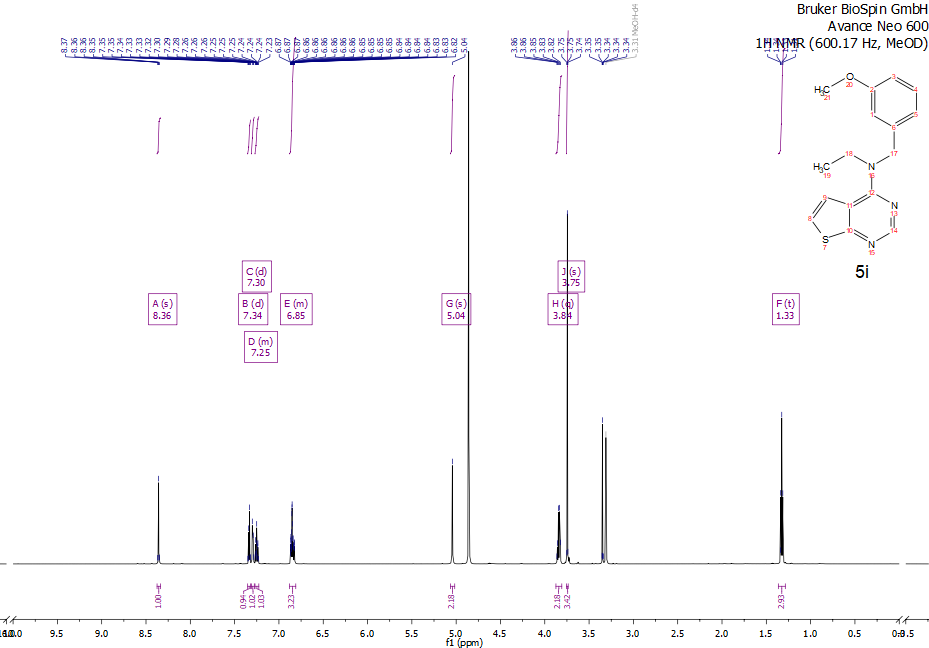
***

***
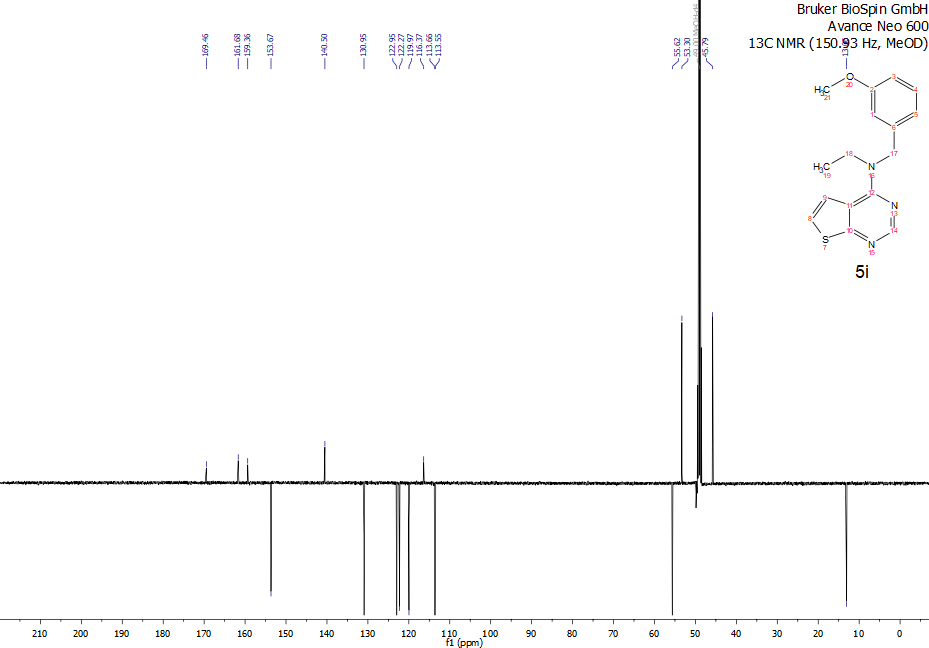
***

***N-*methyl-*N-*phenethylthieno[2,3*-d*]pyrimidin-4-amine, 5j**

***
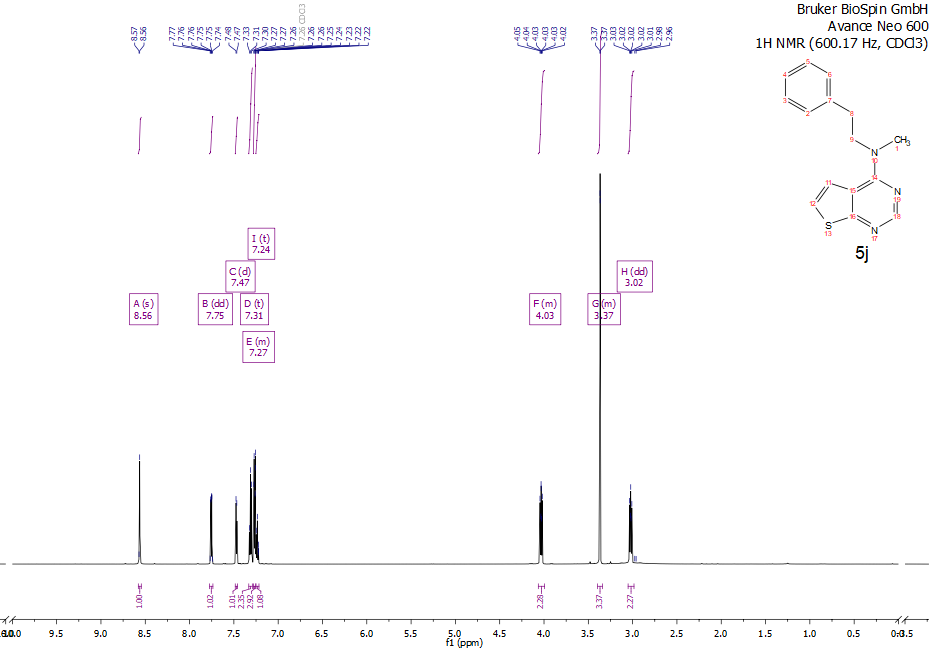
***

***
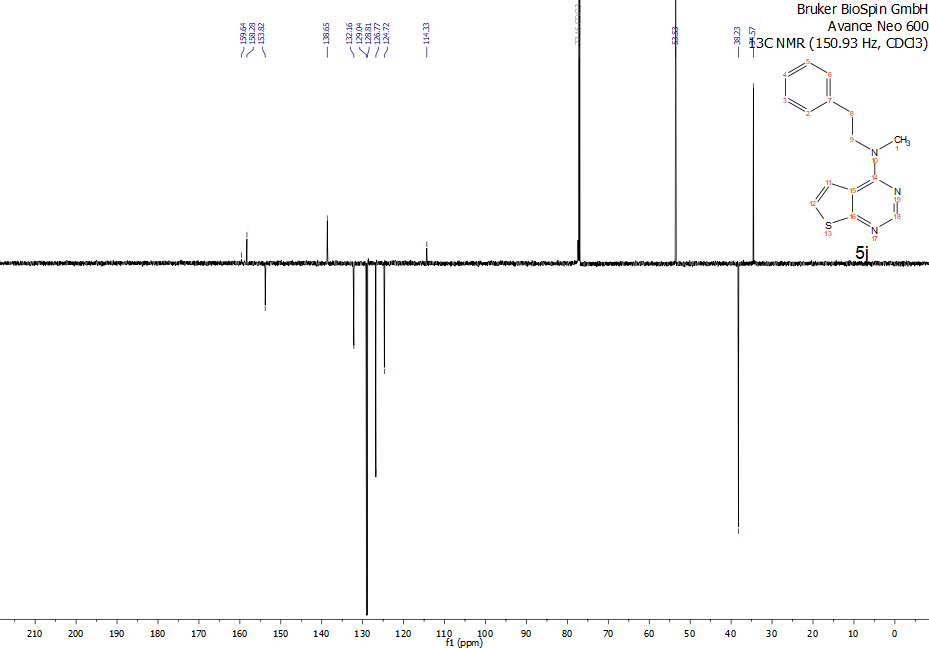
***

***N-*(2-chlorophenethyl)thieno[2,3*-d*]pyrimidin-4-amine, 5k**

***
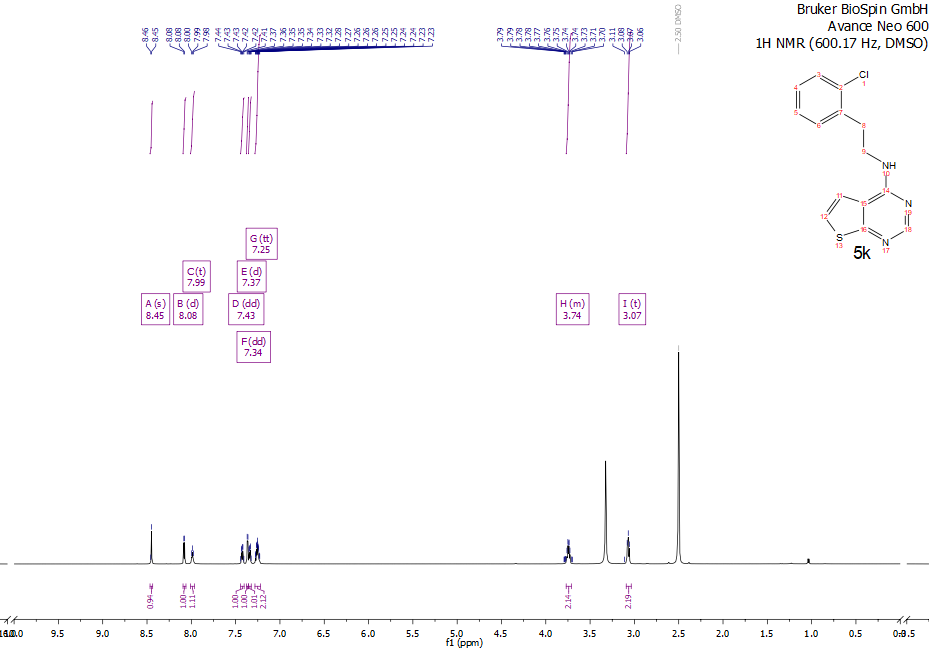
***

***
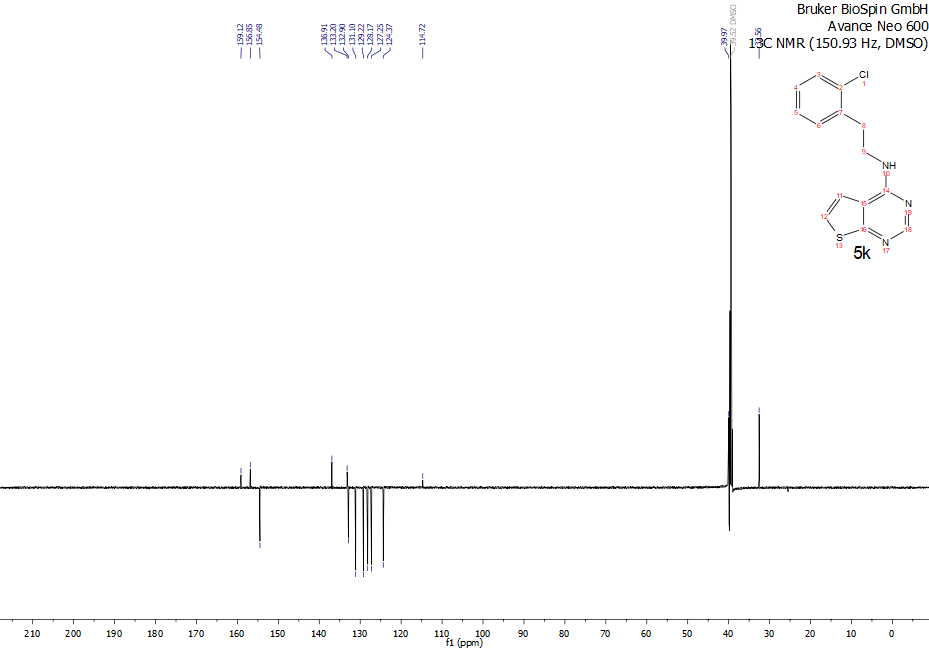
***

***N-*(3-chlorophenethyl)thieno[2,3*-d*]pyrimidin-4-amine, 5l**

***
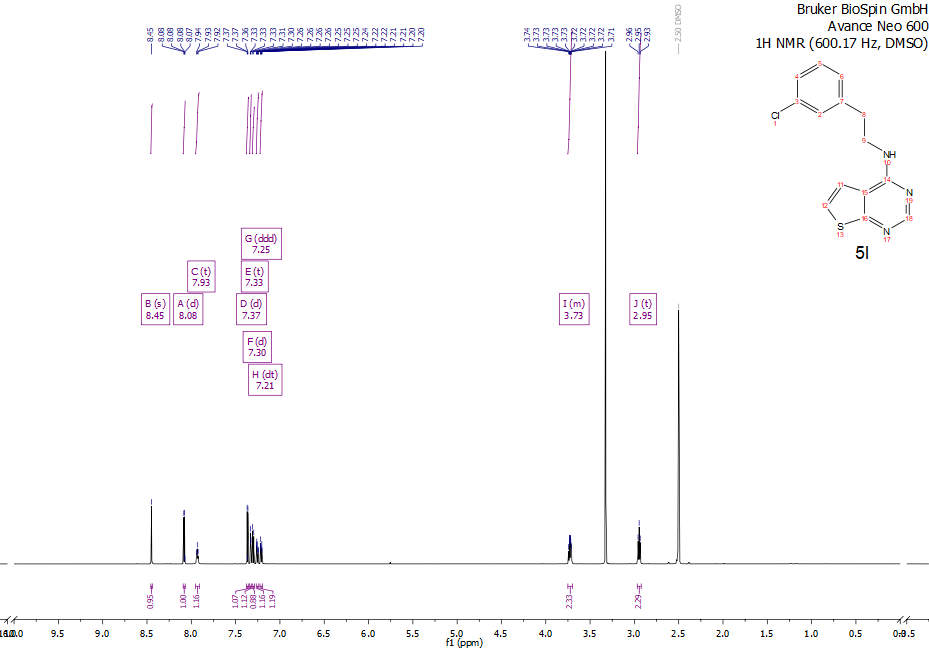
***

***
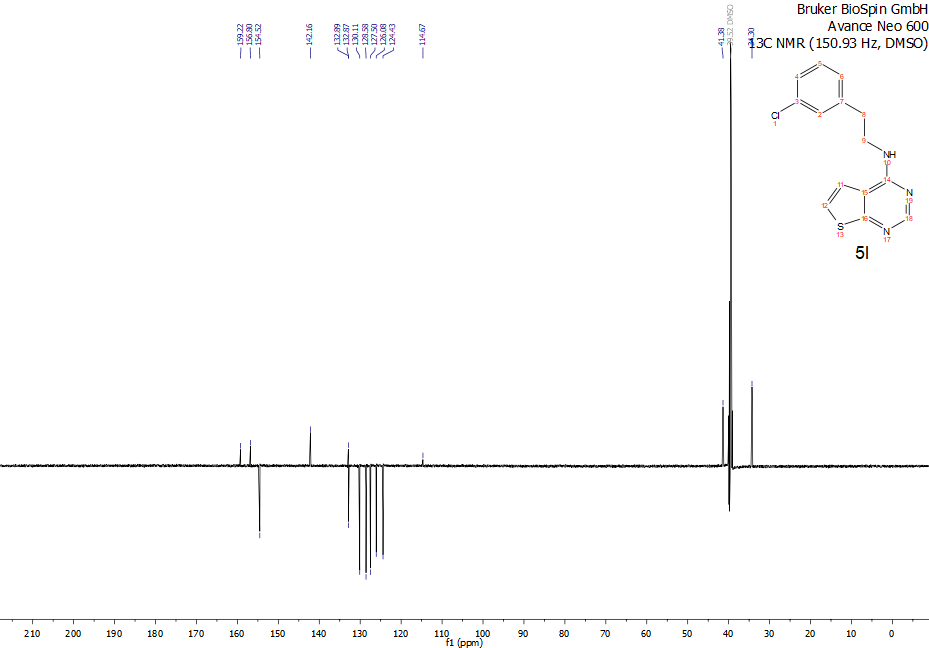
***

***N-*(4-chlorophenethyl)thieno[2,3*-d*]pyrimidin-4-amine, 5m**

***
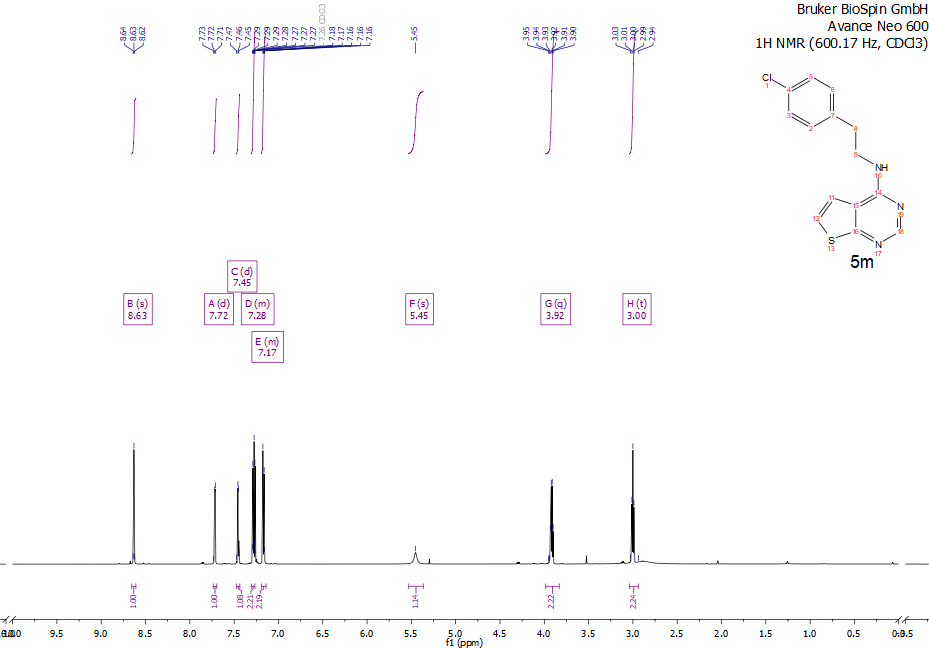
***

***
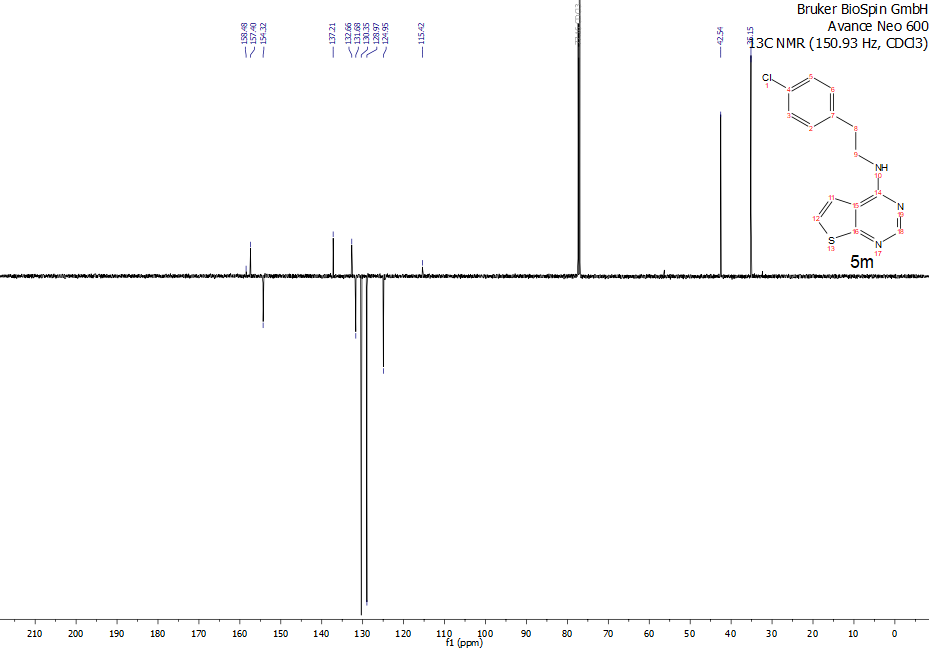
***

***N-*(3-methoxyphenethyl)thieno[2,3*-d*]pyrimidin-4-amine, 5n**

**
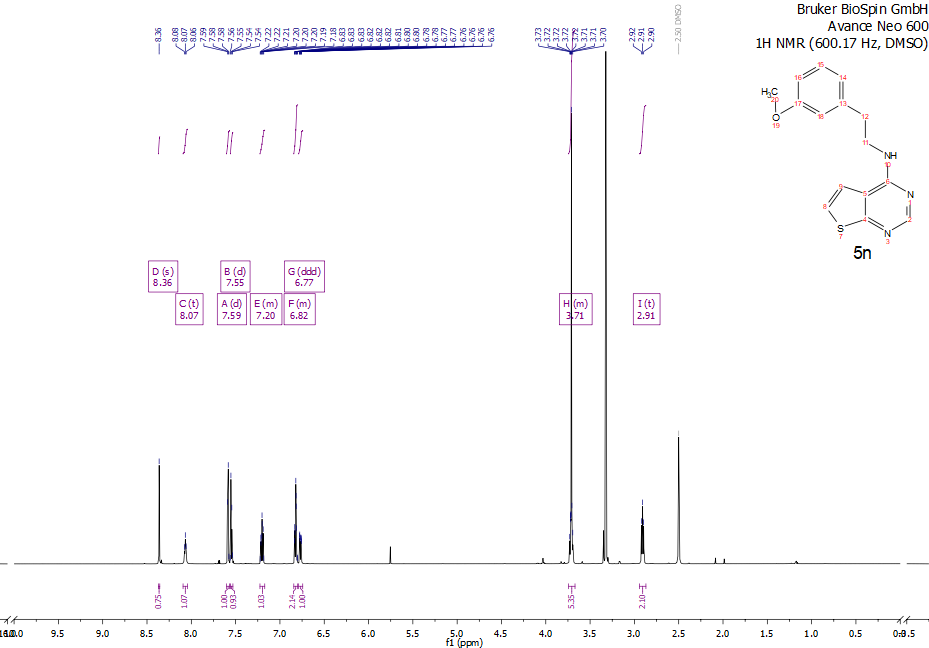
**

**
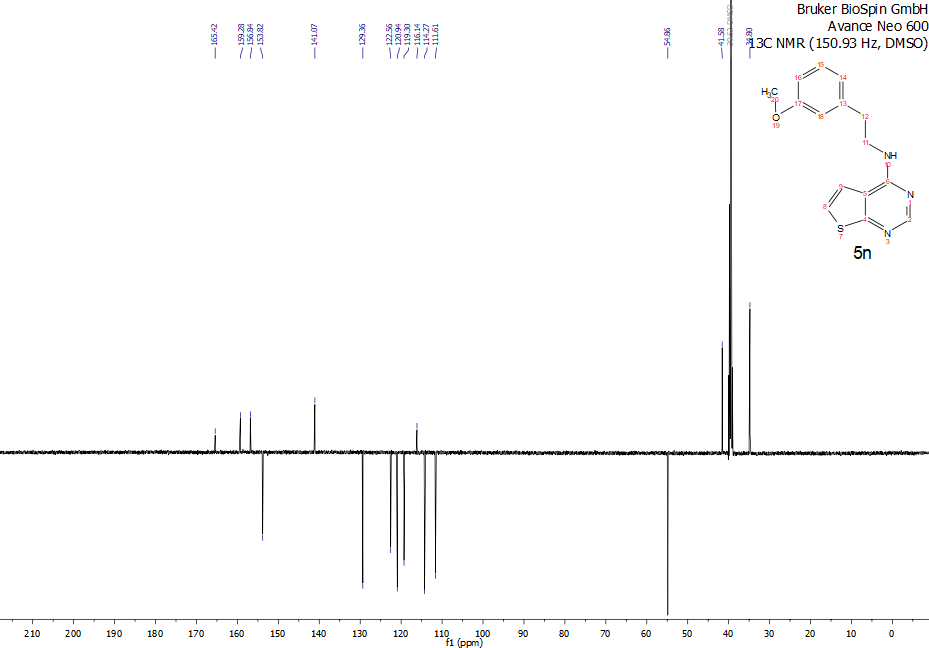
**

**Thieno[2,3*-d*]pyrimidin-4-amine, 6**

**
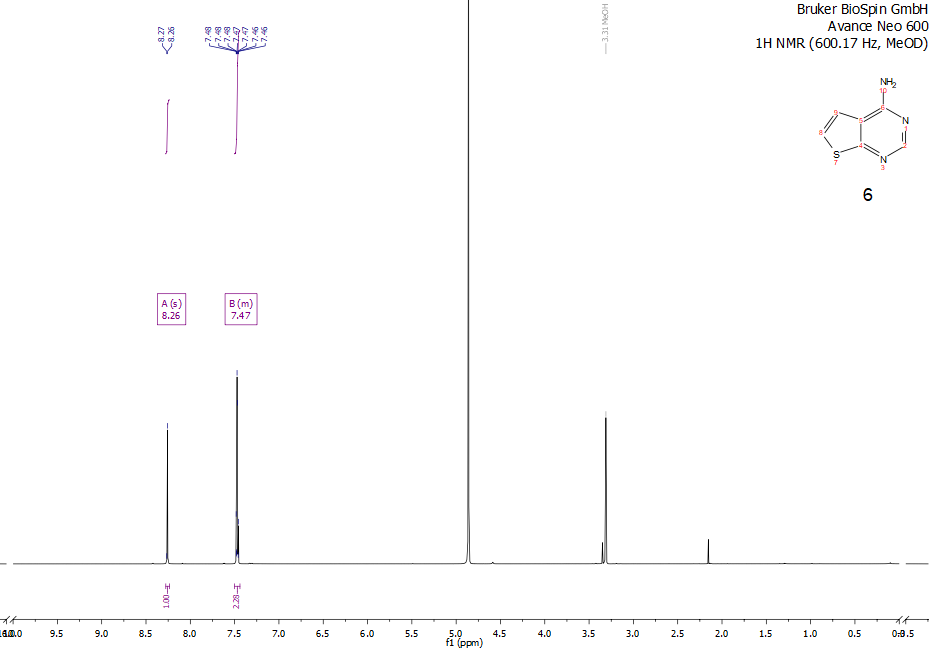
**

**3-methoxy-*N-*(thieno[2,3*-d*]pyrimidin-4-yl)benzamide, 7**

**
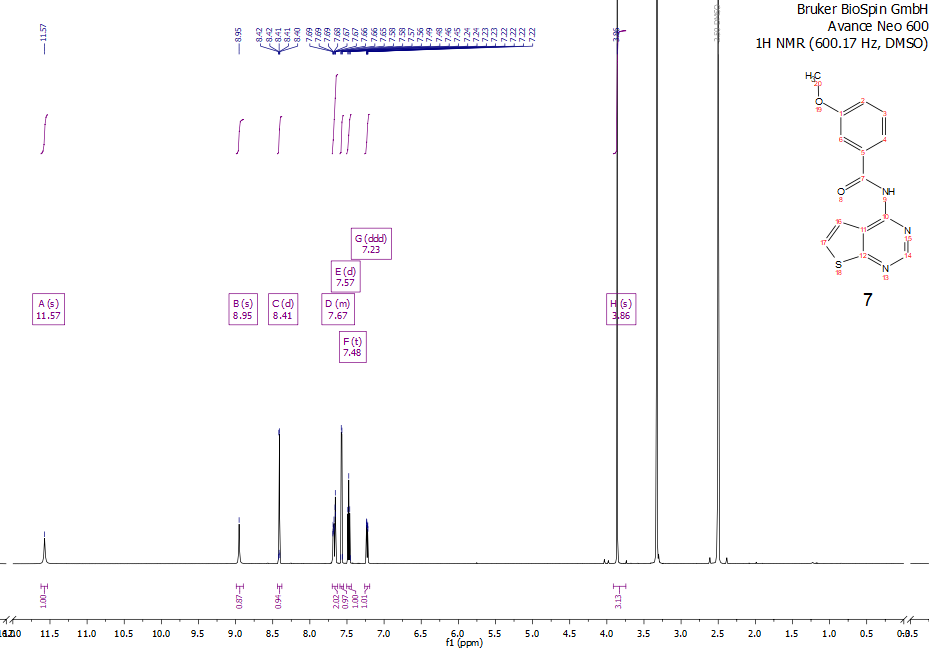
**

**
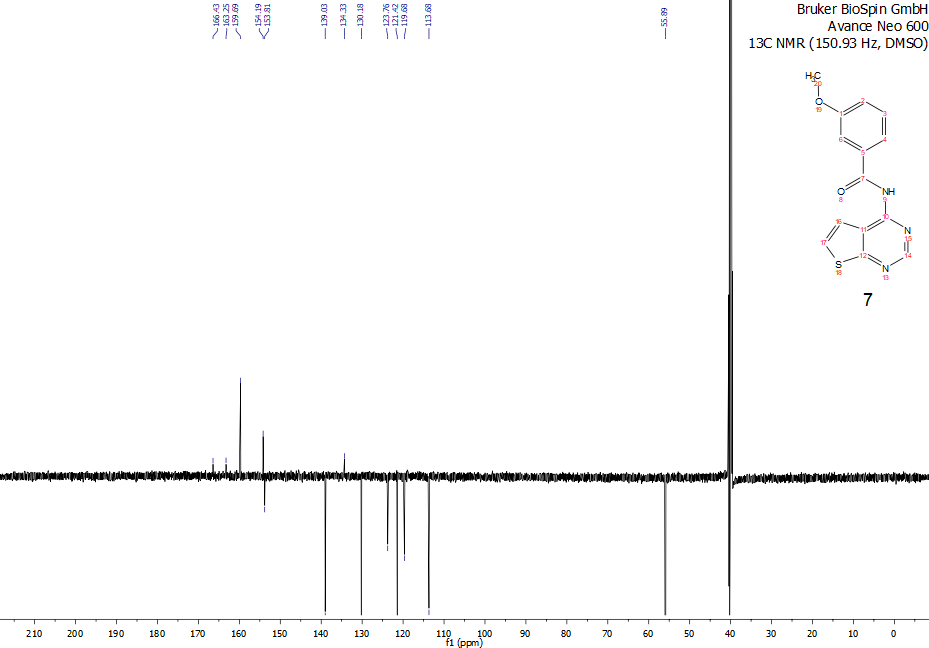
**

**2-chloro-*N-*(2-methoxybenzyl)quinazolin-4-amine, 8a**

**
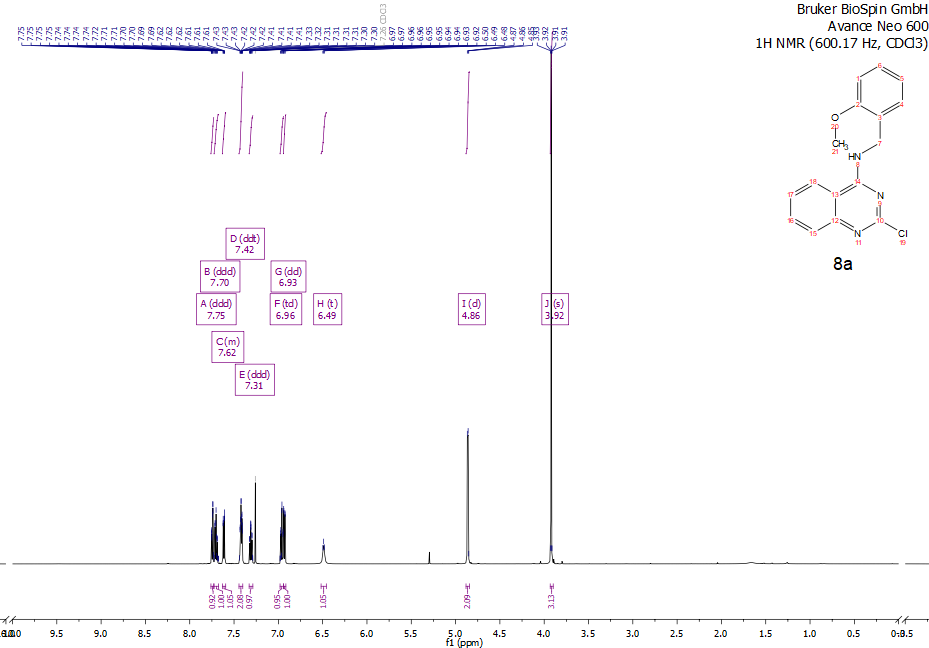
**

**
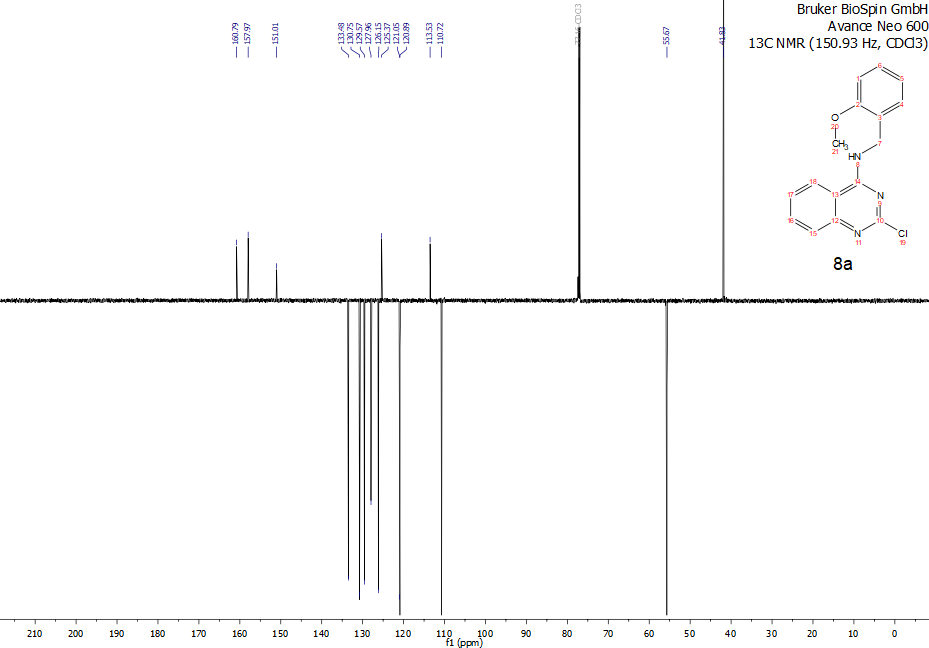
**

**2-chloro-*N-*(3-methoxybenzyl)quinazolin-4-amine, 8b**

**
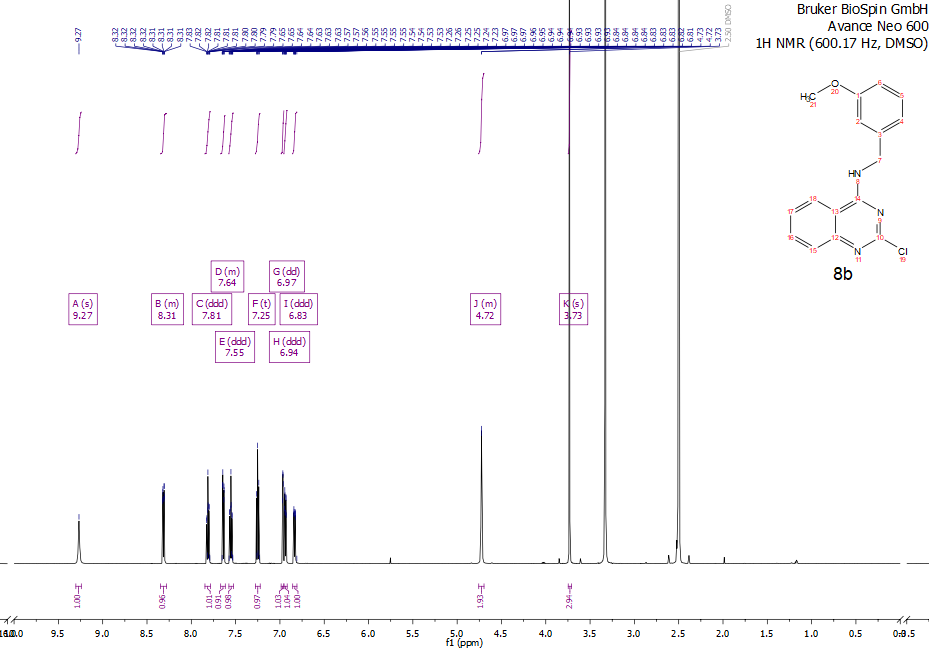
**

**
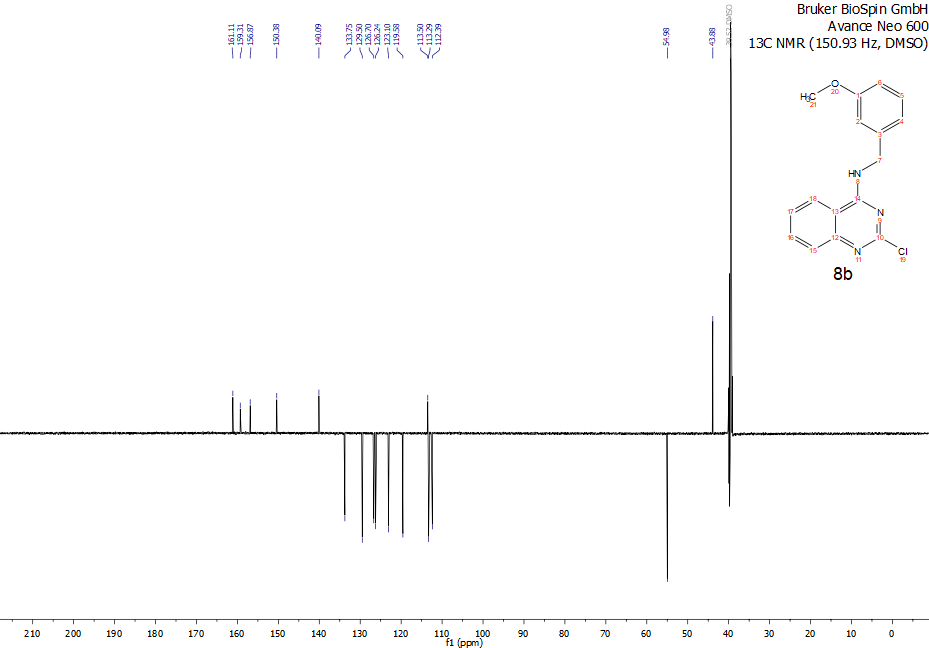
**

**2-chloro-*N-*(4-methoxybenzyl)quinazolin-4-amine, 8c**

**
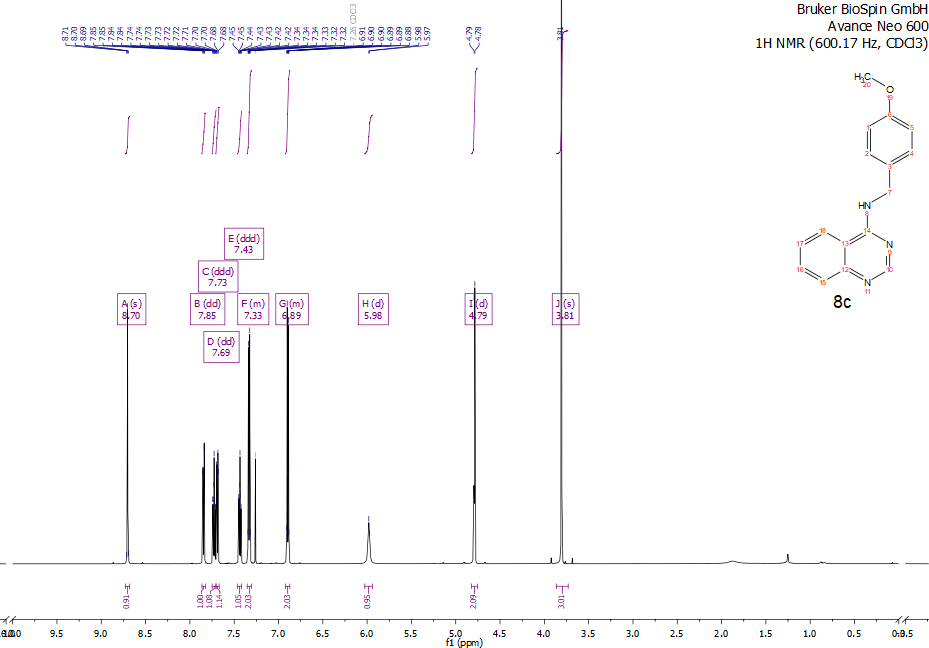
**

**
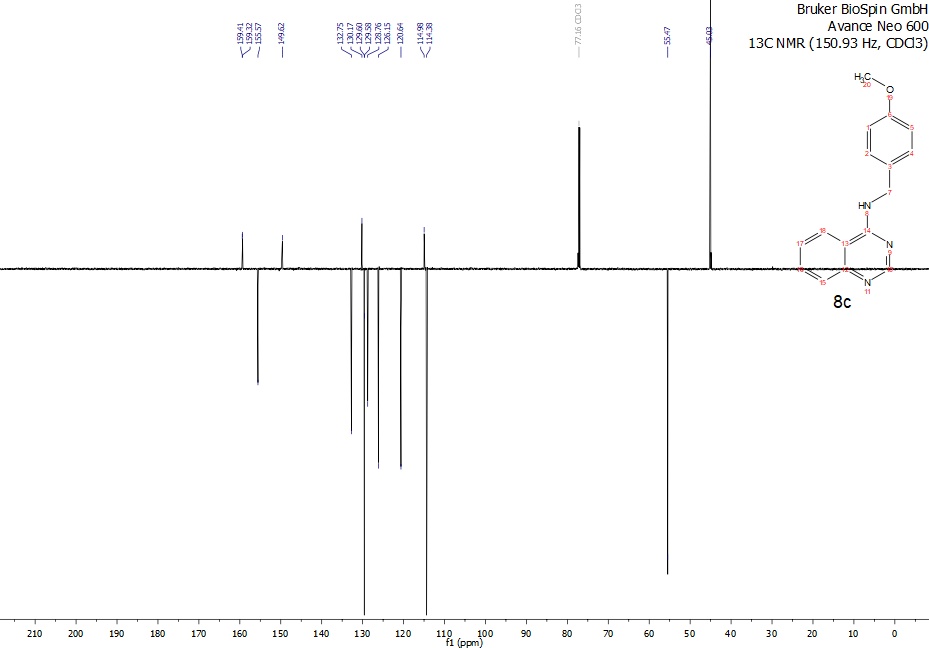
**

**2-chloro-*N-*(4-fluorobenzyl)quinazolin-4-amine, 8d**

**
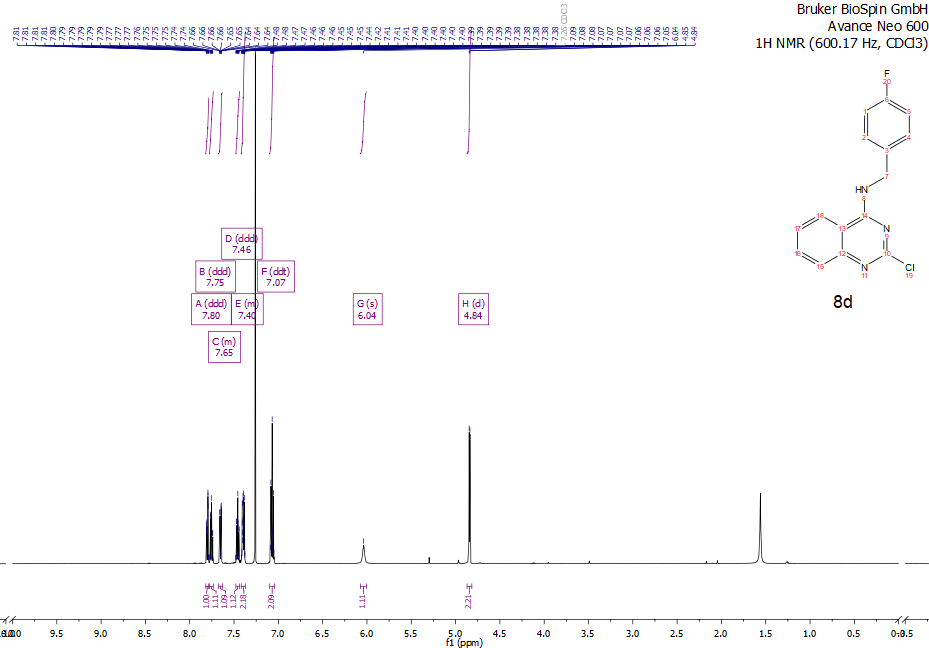
**

**
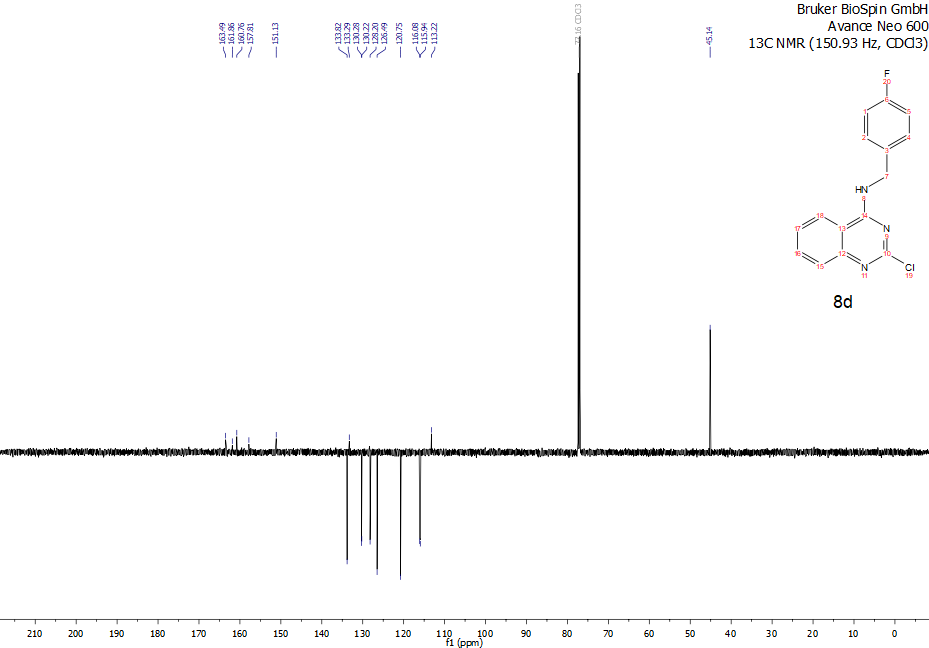
**

**2-chloro-*N-*(4-chlorobenzyl)quinazolin-4-amine, 8e**

**
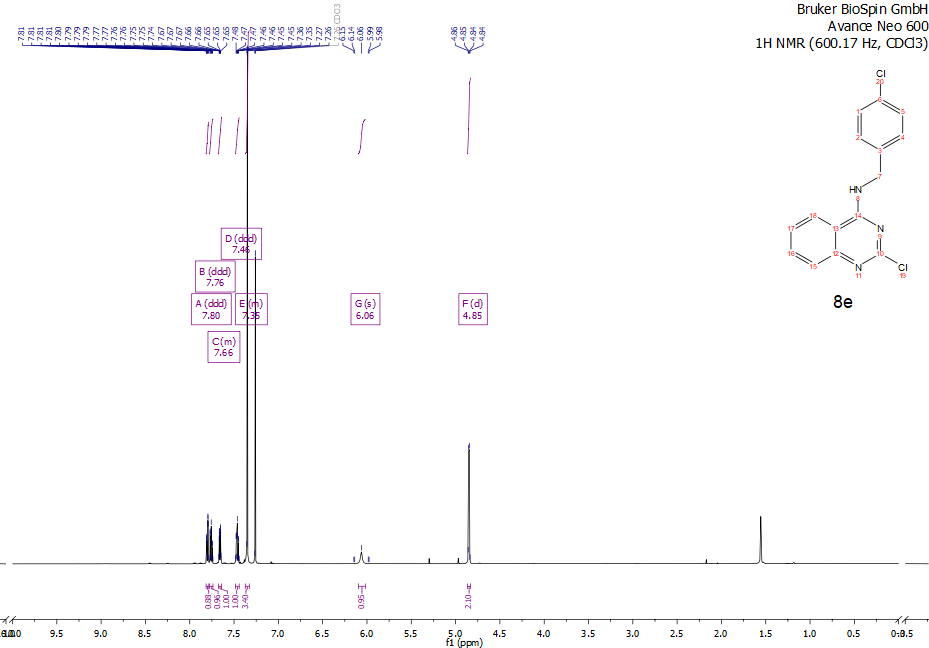
**

**
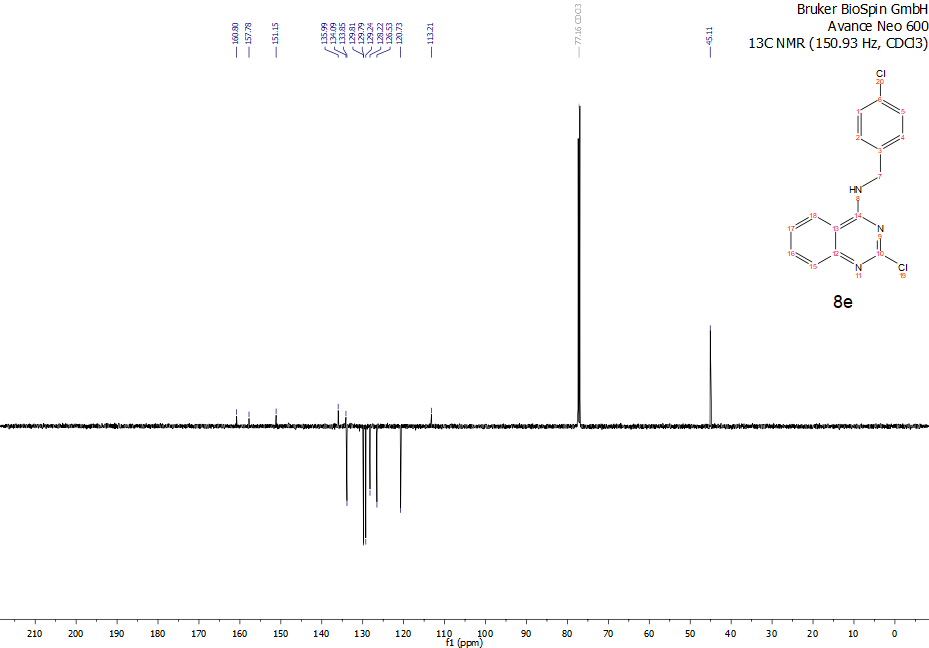
**

**2-chloro-*N-*(4-methylbenzyl)quinazolin-4-amine, 8f**

**
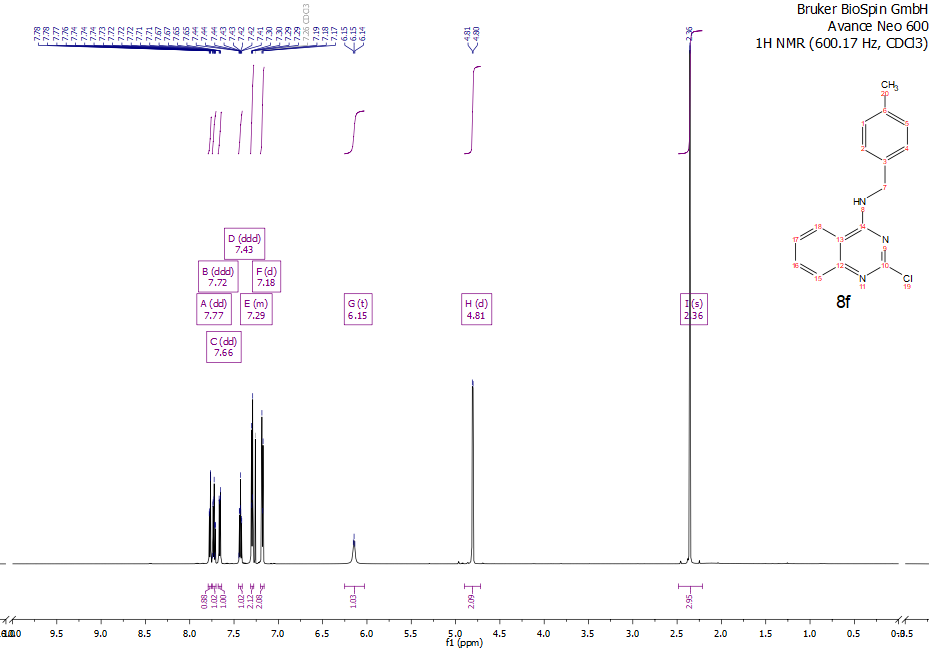
**

**
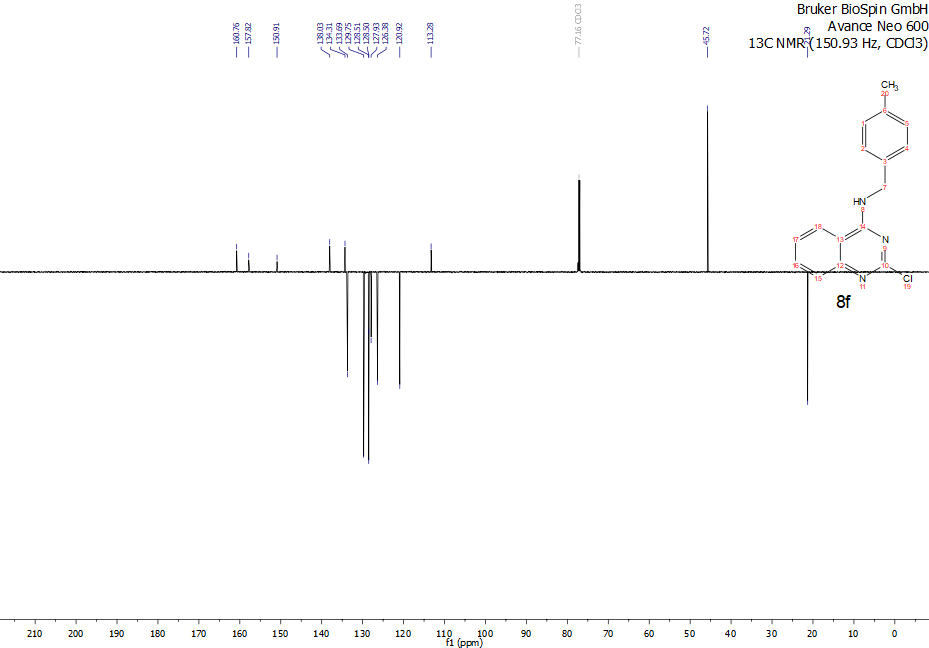
**

**2-chloro-*N-*(4-chlorophenethyl)quinazolin-4-amine, 8g**

**
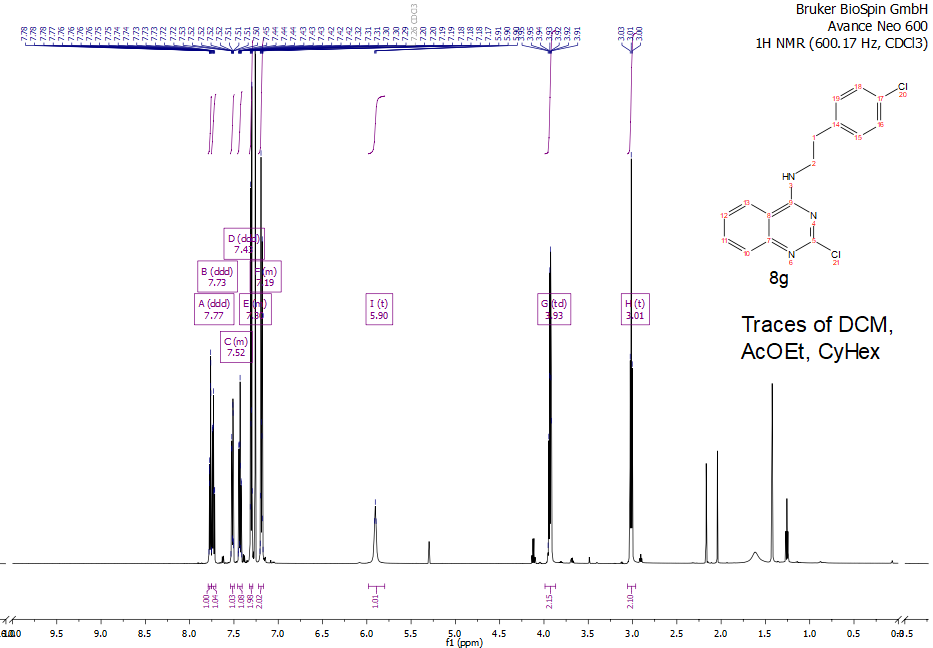
**

**
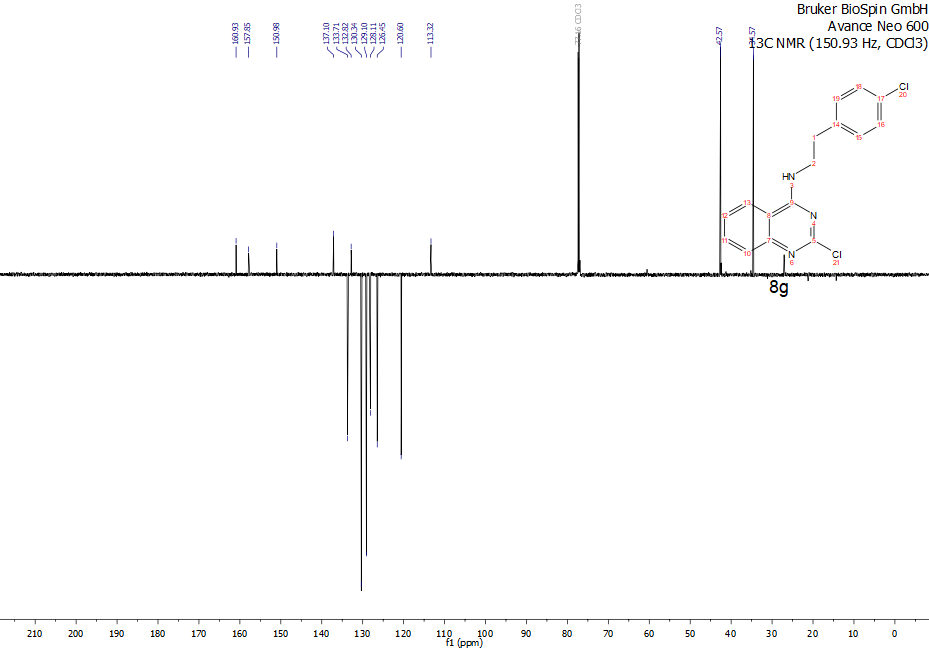
**

**2-chloro-*N-*(1-(4-fluorophenyl)ethyl)quinazolin-4-amine, 8h**

**
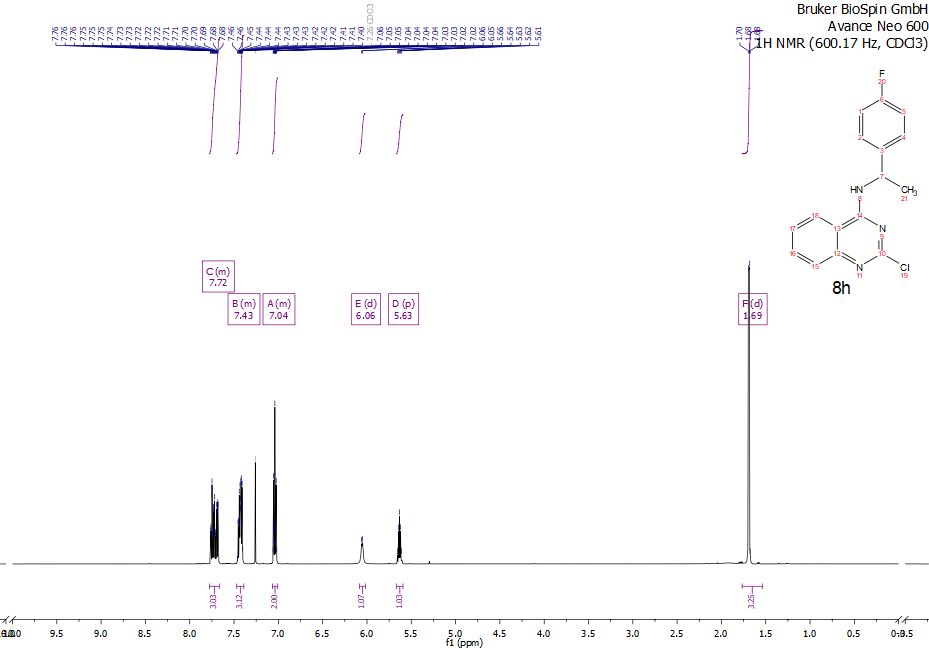
**

**
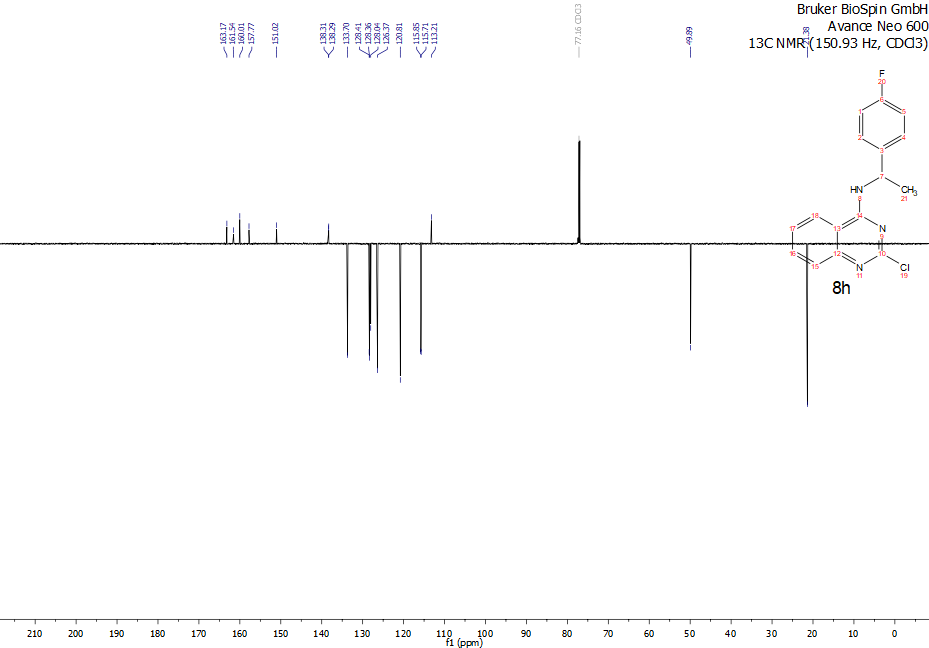
**

**2-chloro-*N-*(1-(p-tolyl)ethyl)quinazolin-4-amine, 8i**

***
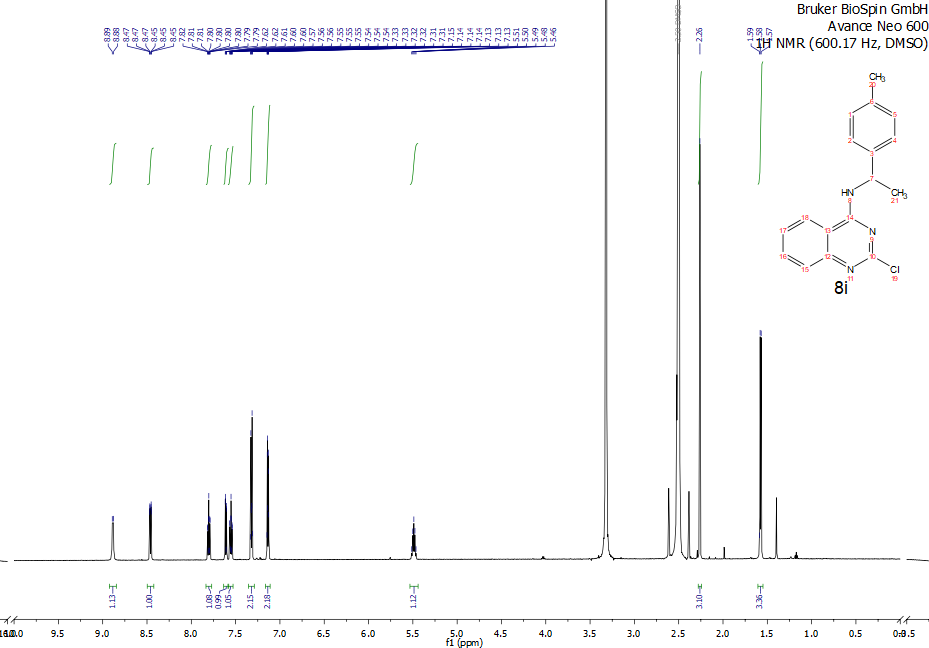
***

***N-*(2-methoxybenzyl)quinazolin-4-amine, 9a**

***
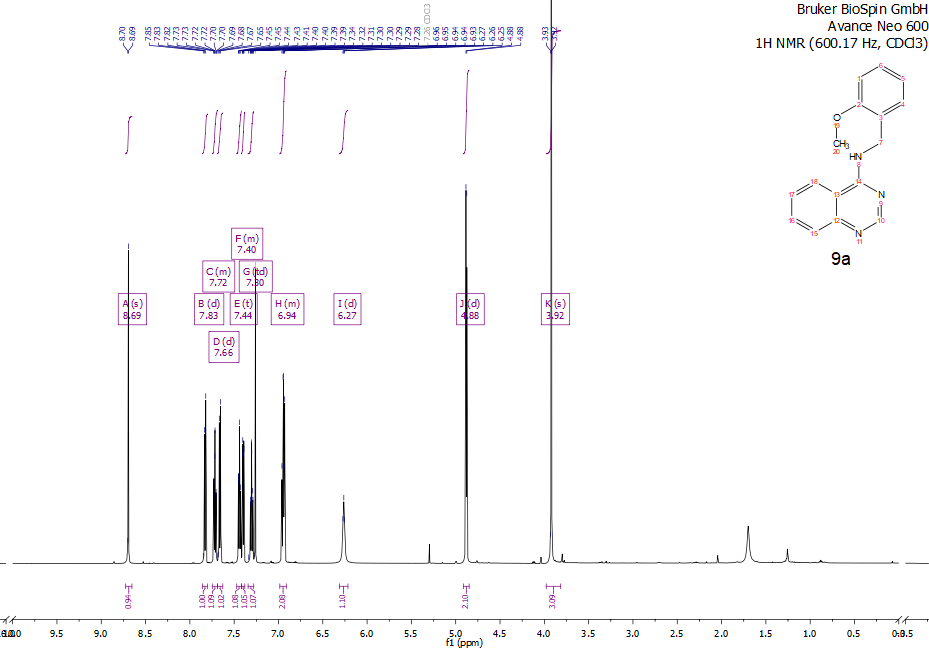
***

***
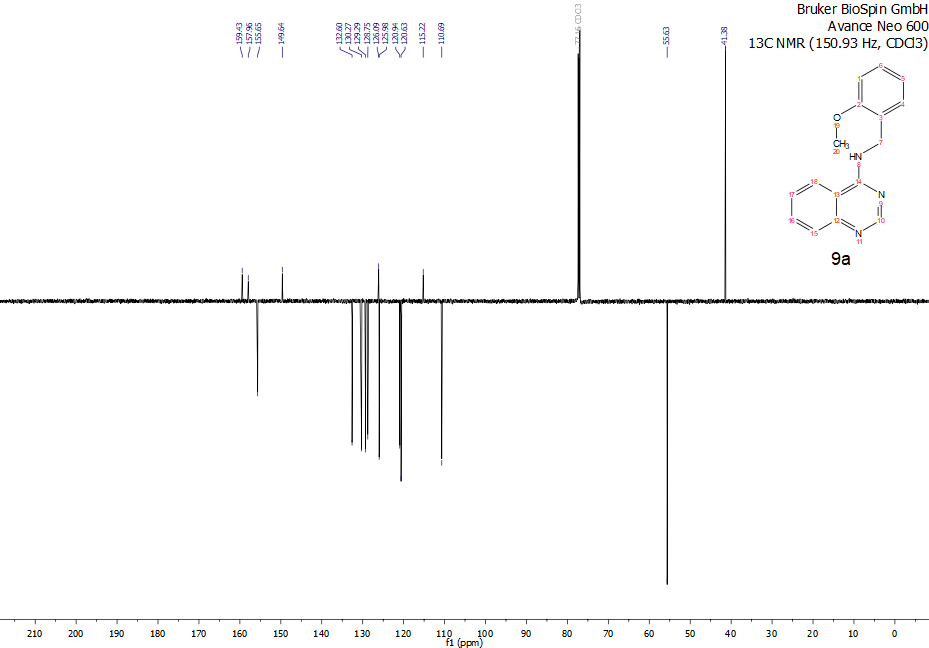
***

***N-*(3-methoxybenzyl)quinazolin-4-amine, 9b**

***
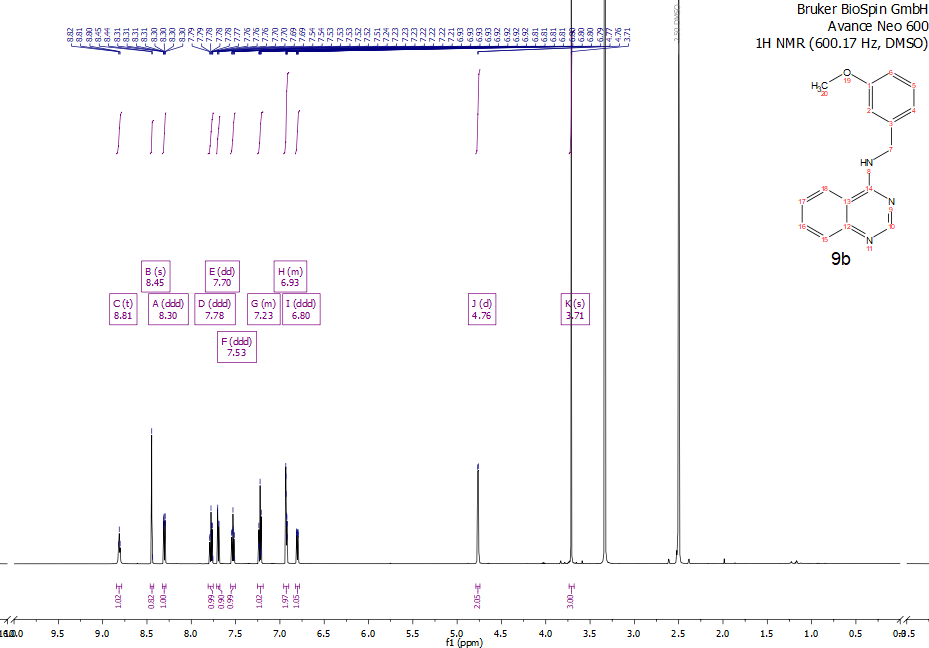
***

***
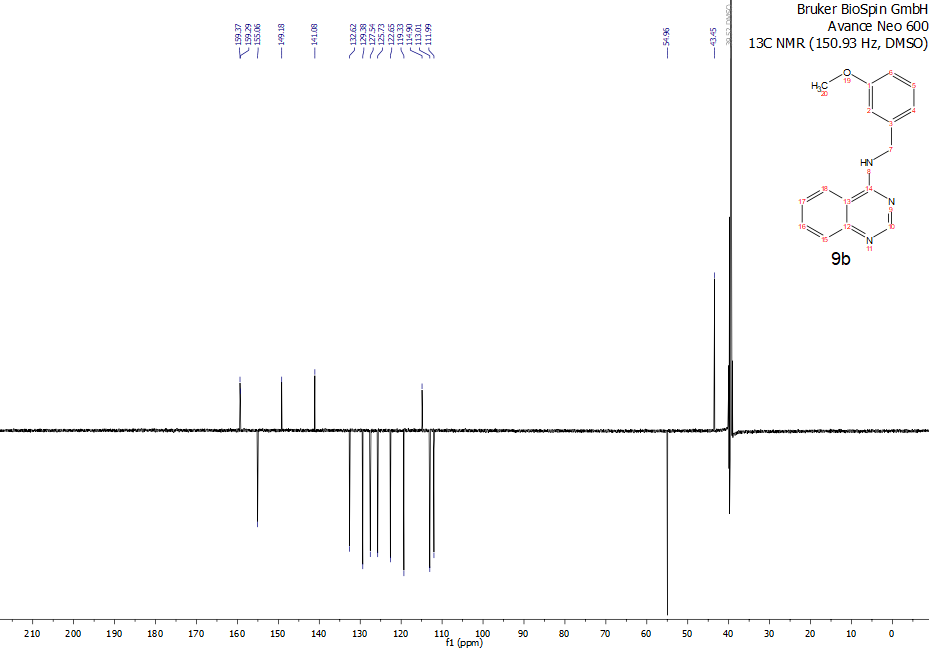
***

***N-*(4-methoxybenzyl)quinazolin-4-amine, 9c**

***
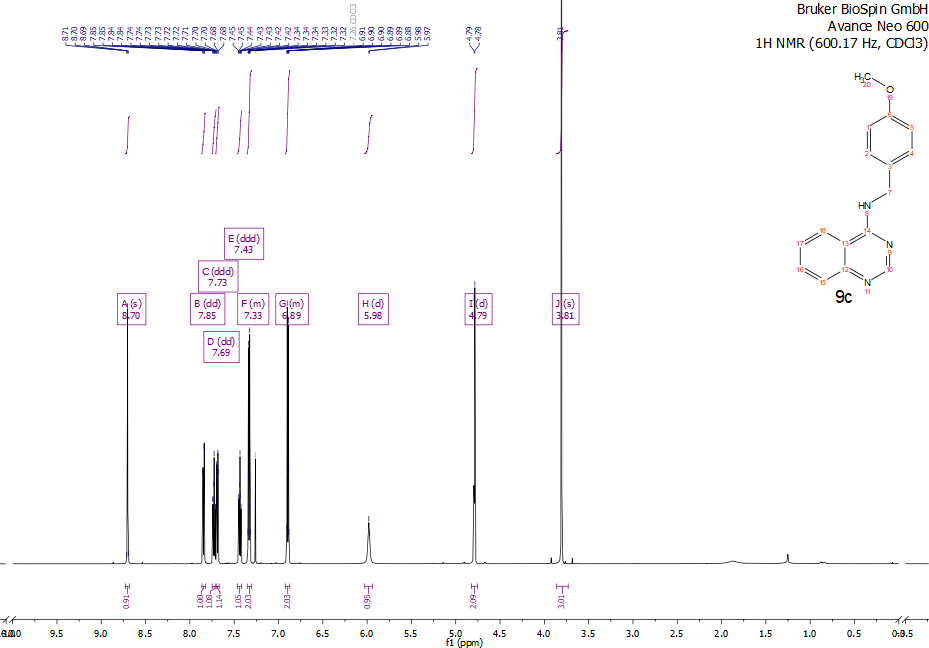
***

***
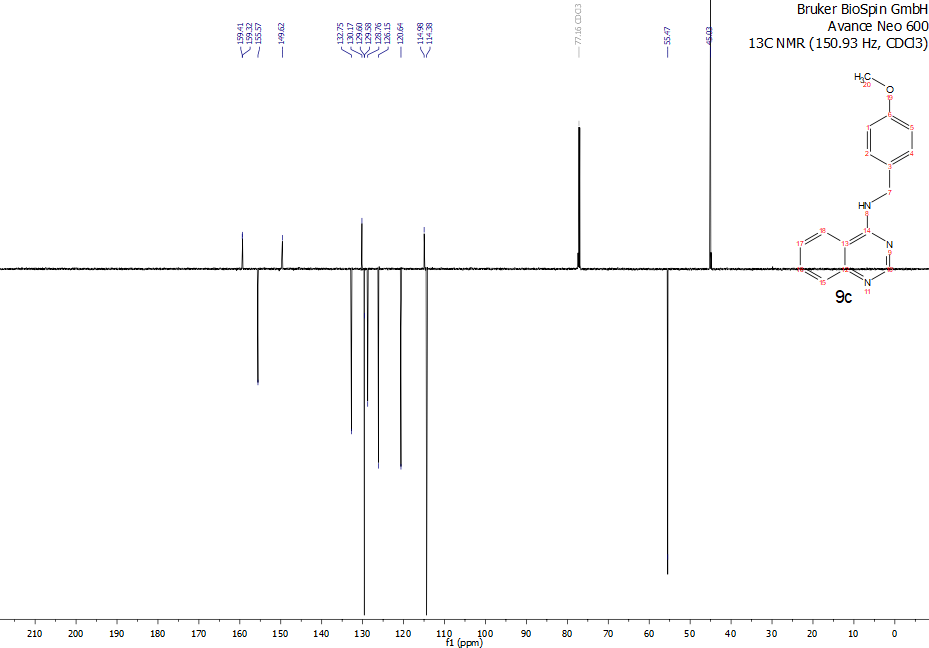
***

***N-*(4-fluoro)quinazolin-4-amine, 9d**

***
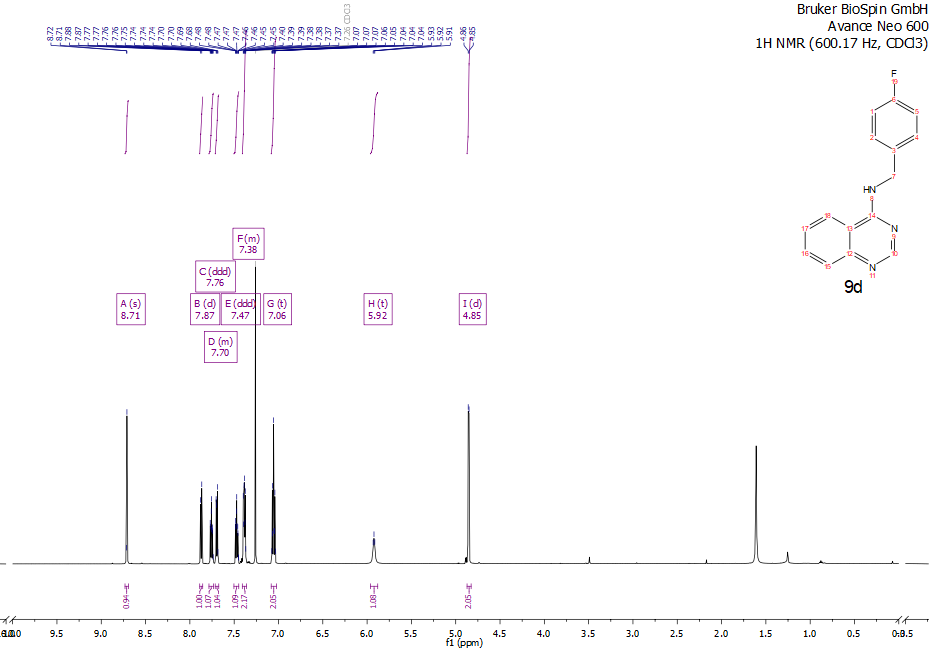
***

***
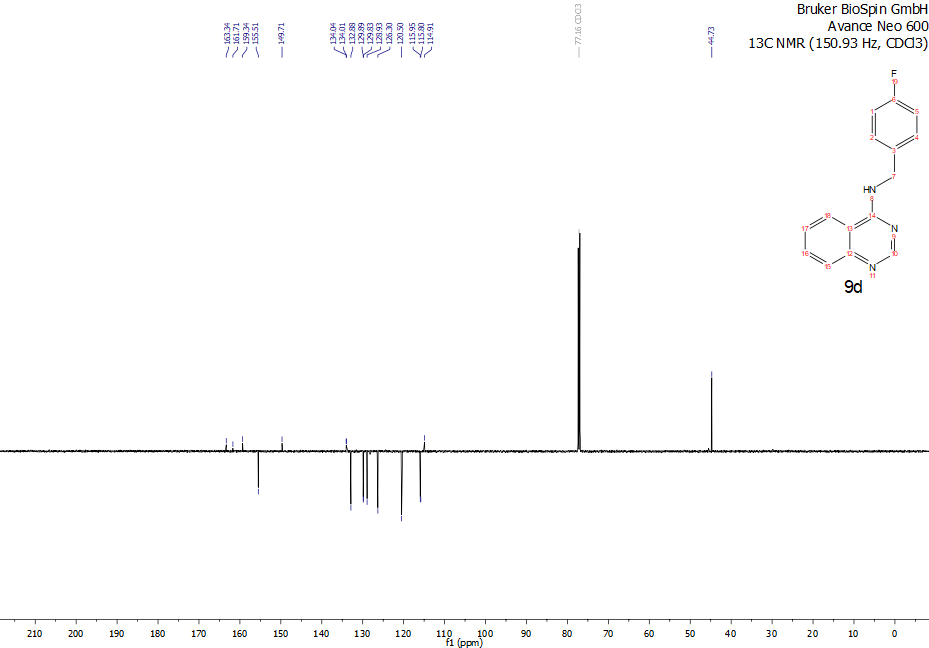
***

***N-*(4-chloro)quinazolin-4-amine, 9e**

***
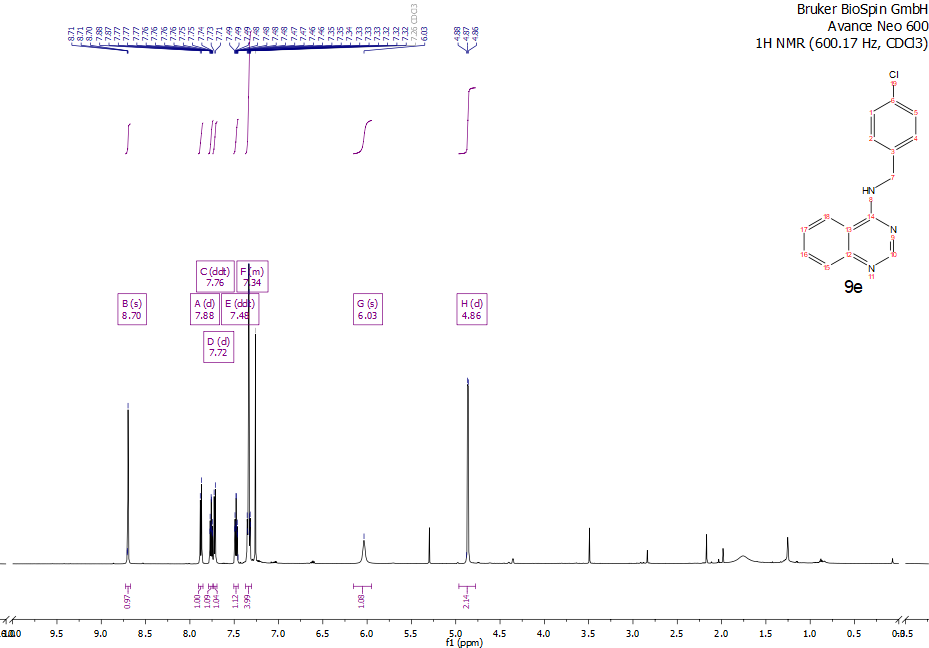
***

***
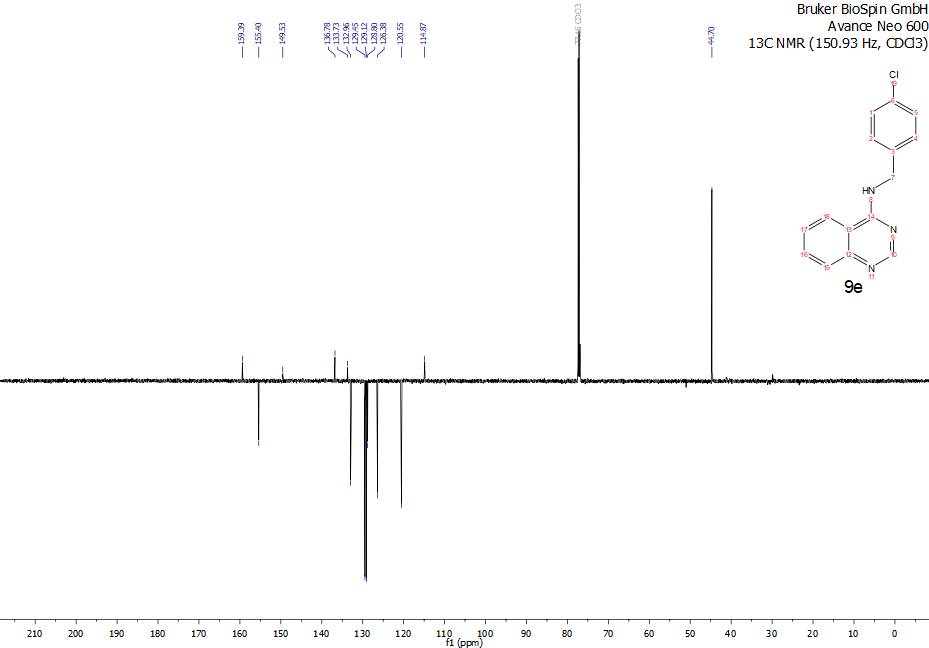
***

***N-*(4-methyl)quinazolin-4-amine, 9f**

***
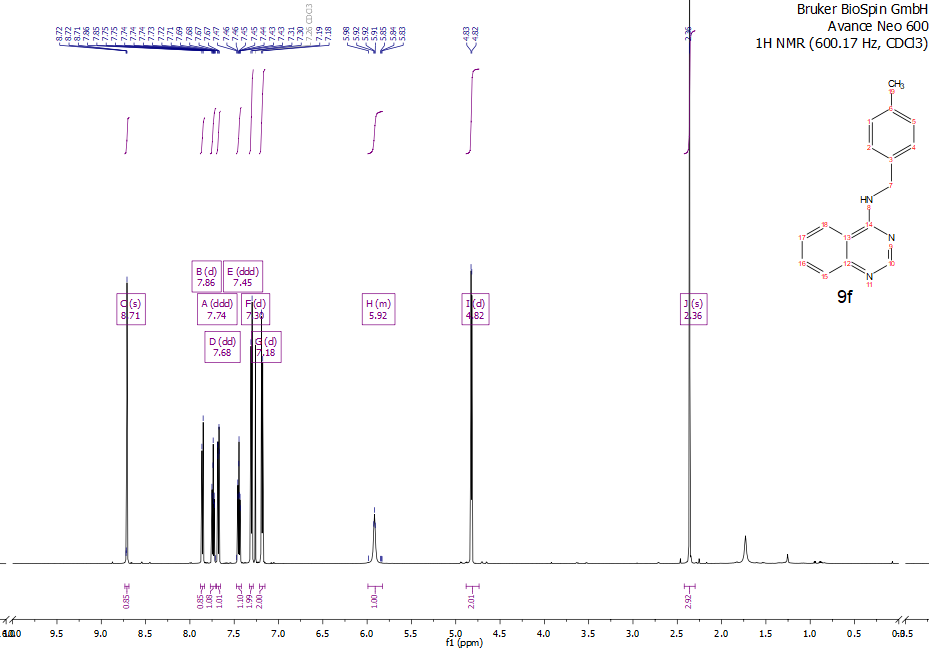
***

***
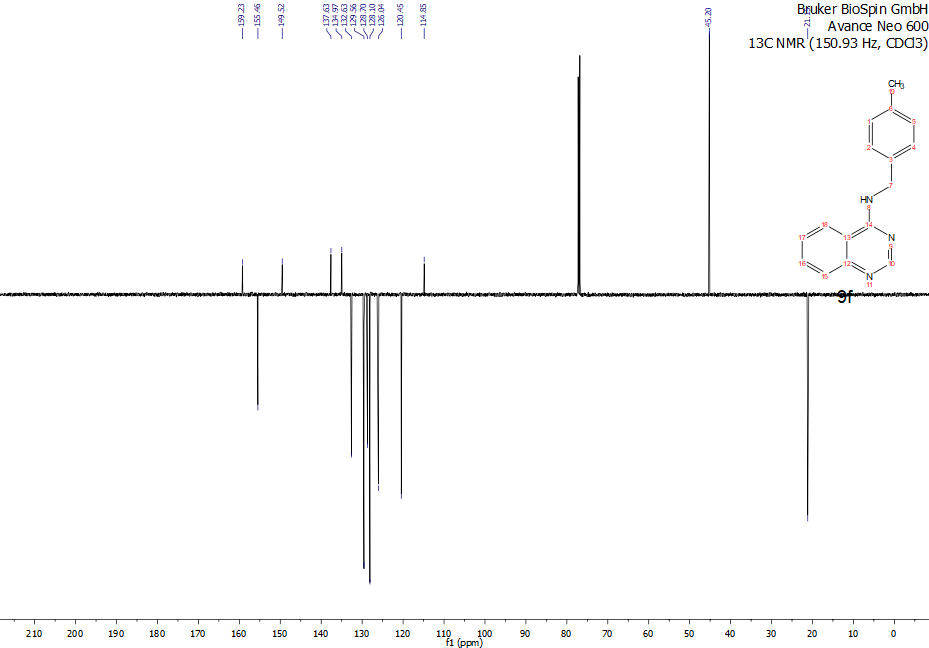
***

***N-*(4-chlorophenethyl)quinazolin-4-amine, 9g**

***
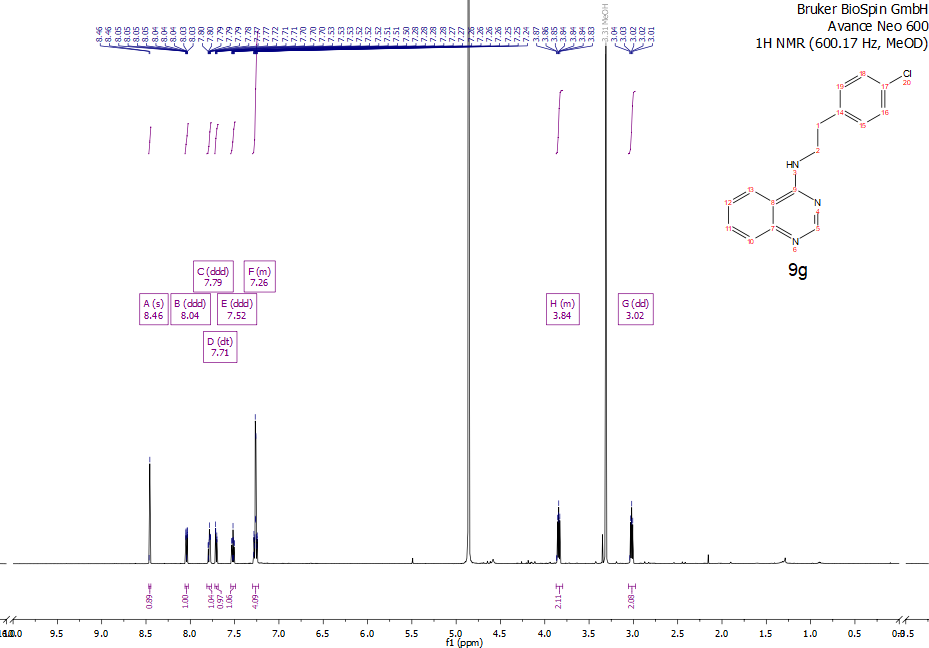
***

***
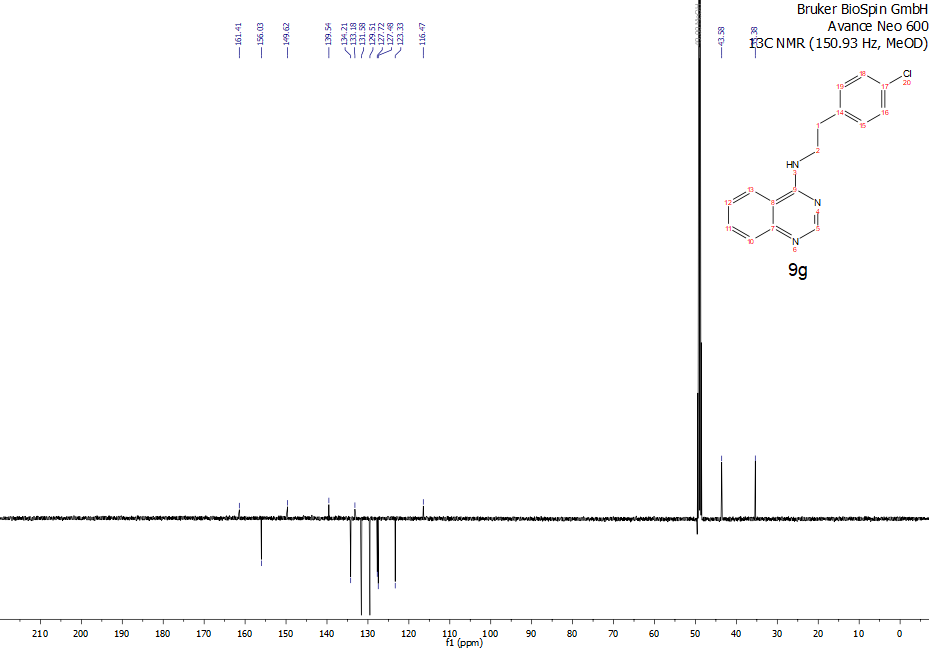
***

***N-*(1-(4-fluorophenyl)ethyl)quinazolin-4-amine, 9h**

***
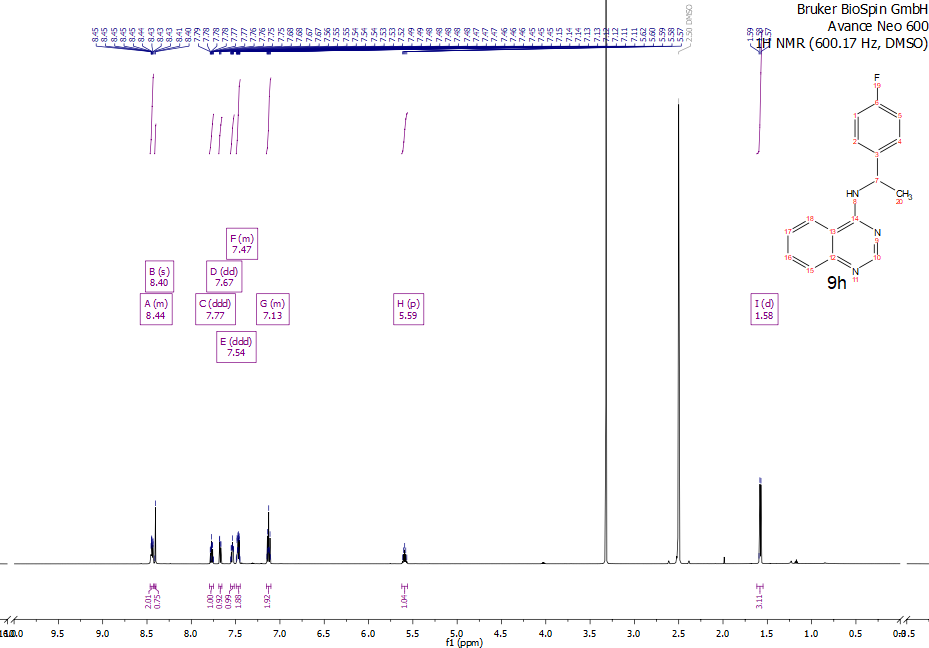
***

***
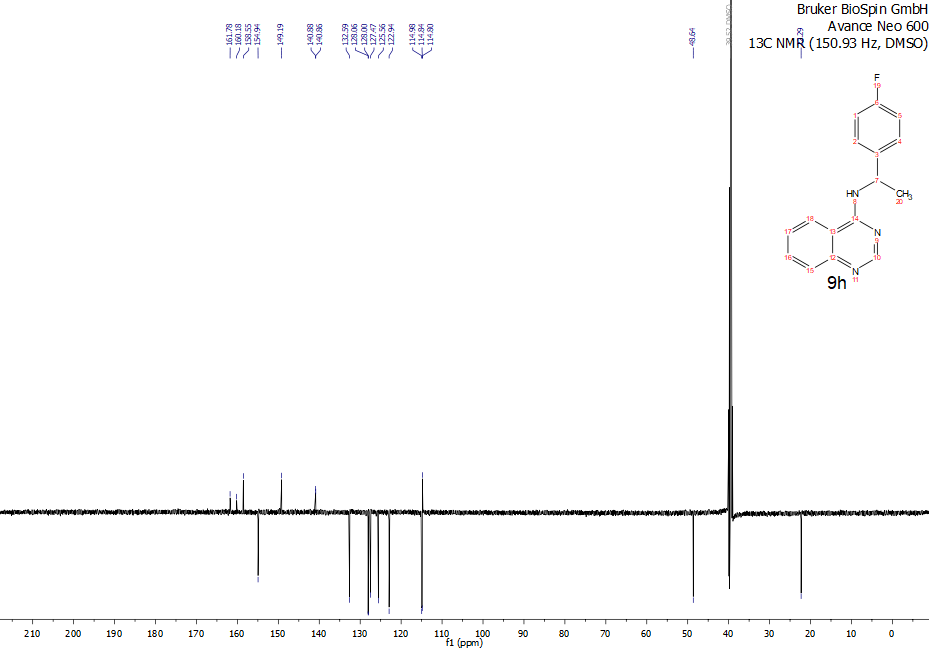
***

***N-*(1-(p-tolyl)ethyl)quinazolin-4-amine, 9i**

***
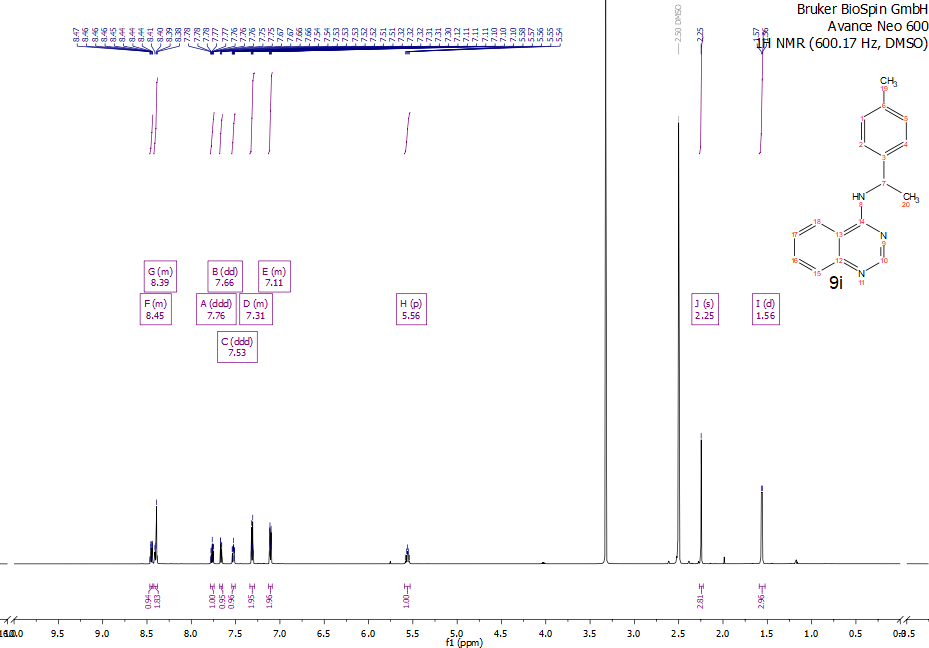
***

***
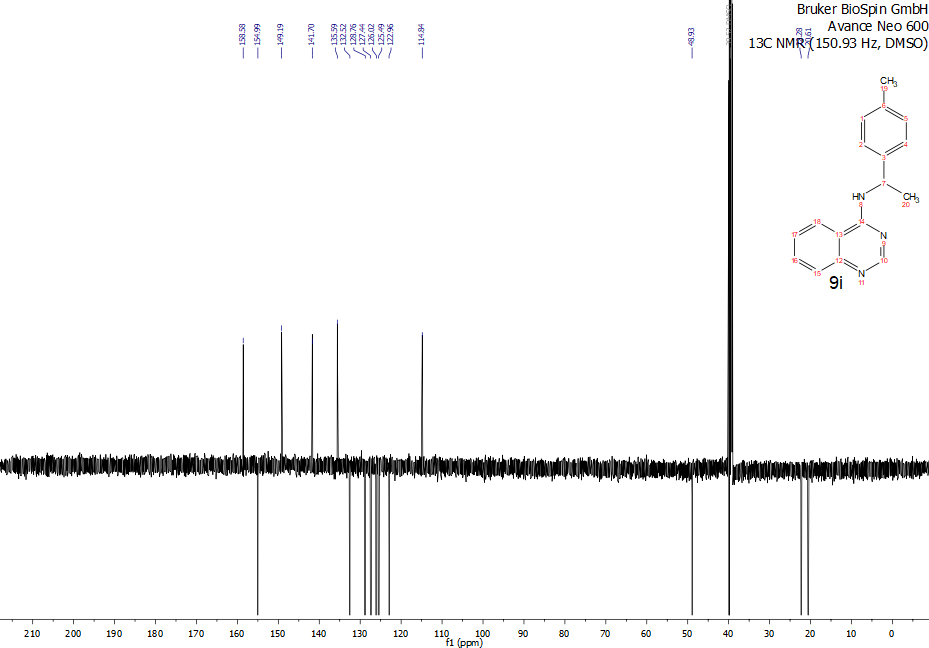
***

***N-*(3-methoxybenzyl)-7-methylthieno[3,2*-d*]pyrimidin-4-amine, 10**

**6-chloro-*N-*(3-methoxybenzyl)pyrimidin-4-amine, 11**

***N-*(3-methoxybenzyl)pyrimidin-4-amine, 12**

***N-*(3-methoxybenzyl)isoquinolin-1-amine, 13**

***tert-*butyl quinolin-4-ylcarbamate, 14**

***tert-*butyl (3-methoxybenzyl)(quinolin-4-yl)carbamate, 15**

***N-*(3-methoxybenzyl)quinolin-4-amine, 16**
